# Traffic-related air pollution significantly aggravates the detrimental effect of infections on the risk of Alzheimer’s disease and other dementias, especially in non-carriers of *APOE4*

**DOI:** 10.3389/frdem.2025.1668381

**Published:** 2026-01-12

**Authors:** Vladimir A. Popov, Svetlana Ukraintseva, Hongzhe Duan, Arseniy Yashkin, Julia Kravchenko, Igor Akushevich, Heather Whitson, Konstantin G. Arbeev, Anatoliy I. Yashin

**Affiliations:** 1 Biodemography of Aging Research Unit, Social Science Research Institute, Duke University, Durham, NC, United States; 2 Department of Surgery, Duke University School of Medicine, Durham, NC, United States; 3 Center for the Study of Aging and Human Development, Duke University School of Medicine, Durham, NC, United States

**Keywords:** aging, air pollution, Alzheimer’s disease, APOE4, dementia, infections, TRAP

## Abstract

Alzheimer’s disease (AD) is a complex neurodegenerative disorder influenced by various factors, including genetic and exposure-related. Certain combinations of these factors may promote AD more substantially than others. APOE4 is the strongest genetic risk factor for AD. Traffic-related air pollution (TRAP) and infections are important exposure-related AD risk factors. Here we investigated how the interplay between a history of infections and chronically high exposure to TRAP (highTRAP) impacts the subsequent risk of AD and other dementias (AD+) in carriers and non-carriers of APOE4 in UK Biobank (UKB) participants aged 60–75 years. HighTRAP was approximated by the proximity (50 meters or less) of a participant’s primary residence to a major road. Chi-square, Wilson score interval, Wald interval, Wald risk ratio, Welch tests, and regression were used to examine statistical significance. We found that UKB participants with a history of various infections (by ICD-10 codes), but without highTRAP, had a 54% increase in AD+ risk. HighTRAP alone did not significantly influence AD+ risk. Individuals with both a history of infections and highTRAP had a 164% higher risk of AD+ compared to those without either factor. That risk was much higher (349%) in non-carriers of APOE4 but became non-significant in APOE4 carriers. We conclude that avoiding high exposure to TRAP may significantly reduce the risk of AD in non-carriers of APOE4 with a history of infections but not in carriers. One potential explanation could be that APOE4 is a stronger AD risk factor, whose AD-promoting effects may outweigh those of other risk factors.

## 1 Introduction

Alzheimer’s disease (AD) is a complex neurodegenerative disorder whose risk is influenced by many factors, including age, genes, exposures, health issues, and others (Yashin et al. 2018; Ukraintseva et al., 2024; Akushevich et al., 2023; Livingston et al., 2024). A ‘multi-hit’ hypothesis of AD implies that some critical amount of risk factors (‘hits’) is required to trigger AD in an individual (Gong et al., 2018; Patrick et al., 2019; Steele et al., 2022). The *APOE4* is the strongest genetic risk factor for AD. Infections and traffic-related air pollution (TRAP) have recently emerged as important exposure-related risk factors that may act together with *APOE4* to promote AD-related pathology (Popov et al., 2024; Rajendrakumar et al., 2025).

Infections have been associated with AD in many studies, though exact mechanism is debated, as multiple pathways may be involved (Vigasova et al., 2021; Fülöp et al., 2020; Lotz et al., 2021; Whitson et al., 2022; Ecarnot et al., 2023; Ukraintseva et al., 2024). The roles of neuroinflammation, inflammasome signaling, microglia, self-reactive T cells, gut-brain axis, and amyloid beta as a potential antimicrobial protein, have been in major focus of recent research (Whitson et al., 2022; Ecarnot et al., 2023). Herpesviruses as drivers of AD have also been broadly discussed (Itzhaki, 2017; Wainberg et al., 2021). Our recent study compared associations of very different infectious diseases (bacterial, viral, and fungal) with AD in a large pseudorandomized sample of older adults, and found that they all are associated with AD, while different vaccines were protective. This indicates that compromised immunity could play a central role in the associations between infections and AD rather than a specific pathogen (Ukraintseva et al., 2024).

A growing body of research also points to a connection between exposure to air pollution and neurodegenerative disorders, including AD, though mechanisms are not fully understood (Yuan et al., 2023; Parra et al., 2022; Tham and Schikowski, 2021; Finch, 2023; Franz et al., 2023). Various pollutants are present in the air, and some may pose risks to human health. For example, inhalable particulate matter (PM) and nitrogen dioxide (NO_2_) have been intensively studied in this regard (Craig et al., 2008; Akimoto, 2003; Dominski et al., 2021). A recent analysis of UK Biobank (UKB) data found that higher exposure to PM2.5 (median particle with diameter ≤ 2.5 μm) and NO_2_ is associated with multimorbidity in a dose-dependent manner (Ronaldson et al., 2022). The PM, NO_2_, and volatile organic compounds (VOCs) are common components of the traffic-related air pollution (TRAP). These and other types of air pollution (such as ozone, sulfur oxides, carbon monoxide, and lead), might be harmful to the central nervous system (CNS) and promote neuroinflammation and neurodegeneration (Costa et al., 2020; Cheng et al., 2016; Calderón-Garcidueñas et al., 2002, 2015; Hogan et al., 2015; Spangenberg and Green, 2017). A review of epidemiological and experimental studies of the role of PM in neurodegeneration emphasized a link between chronic exposure to PM and onsets of cognitive deficits, dementia, and AD (You et al., 2022). A meta-analysis of 14 studies concluded that PM2.5 is a risk factor for dementia, with more limited support for nitrogen oxides, though the authors stressed that these results should be interpreted with caution (Wilker et al., 2023). Higher exposure to NO_2_ itself was associated with lower cortical thickness of brain regions relevant to AD (Crous-Bou et al., 2020). Another study that used the UKB data (Li et al., 2023) reported an association between residential distance to major roads and dementia that was mediated by TRAP, mainly NO_2_. It has been associated with a decline in cognitive function and progression of mild cognitive impairment (MCI) to AD (Jack Jr et al., 2000; Henneman et al., 2009; Qu et al., 2023). Thus, the role of exposure to TRAP in neurodegeneration has been broadly supported by recent research.

Here, we investigate how a history of infections and chronic exposure to TRAP—separately and in combination—may influence the risk of AD and other dementias (AD+) in carriers and non-carriers of *APOE4*, the strongest genetic risk factor for AD. Our goal is to better understand the interplay between these major risk factors within the multifactorial mechanism of AD development.

## 2 Materials and methods

### 2.1 Data and variables

This study was performed using the UKB (UK Biobank, 2023), a population-based study with extensive genetic and phenotypic data for approximately 500,000 individuals from across the UK. Data for the study were obtained (October 2022) from the UKB database. Written informed consent was obtained by the UKB from the participants in accordance with the UK national legislation and the UKB requirements. The latest (at the time of calculations) available information on participants’ withdrawal in UKB was taken into account. All analyses were performed on a subset of the database with individuals recruited starting from 2006 and those having data regarding infectious and parasitic disease. Below, the term ‘infectious’ will be used instead of ‘infectious/parasitic’ for conciseness. The terms ‘infectious disease’ and ‘infection’ were considered interchangeable.

The infectious diseases with the following International Classification of Diseases 10th Revision (ICD10) codes occurring during the period from January 1, 2006 to January 1, 2016 were used for the analysis (UK Biobank, 2023; ICD, 2019): Chapter I: certain infectious and parasitic diseases (A00-B99); Chapter IX: acute pericarditis (I30), acute/subacute endocarditis (I33), acute myocarditis (I40); Chapter X: influenza and pneumonia (J09-J18); Chapter X: other acute lower respiratory infections (J20-J22); Chapter XI: acute appendicitis (K35), acute pancreatitis (K85); Chapter XII: acute lymphadenitis (L04). Since infections were required to occur prior to AD+ diagnosis in our analysis, we excluded cases of infectious diseases that occurred after AD diagnosis and could be a consequence of AD rather than its risk factor.

A chronically high exposure to TRAP was approximated by the participant’s residence distance (in meters) to the nearest major road (DNMR). The DNMR was defined based on the local road network taken from the Ordnance Survey Meridian 2 road network 2009 with scale 1:50000 and 1 meter accuracy (McGarva, 2017; UK Biobank, 2023). The 50-meter cut-off (DNMR<50) was chosen as a reasonable equivalent of a high exposure to TRAP, based on supporting evidence from the literature. For example, a large study published in the Lancet used data from two population-based cohorts, including more than six and a half million adult Canadians, and found that living in close proximity (<50 m) to a major traffic road was associated with significantly elevated incident risk of dementia (Chen et al., 2017).

As for *APOE4*, three groups of participants aged 60–75 were considered: 1) all participants, regardless information about *APOE4* carrier status, 2) *APOE4* carriers, and 3) *APOE4* non-carriers. The *APOE4* status (carrier/non-carrier) was defined by presence/absence of the minor allele (C) of the SNP rs429358, a risk factor for AD. Age was calculated at the baseline date January 1, 2006. For each case, the following groups were defined for our analysis. DNMR (distance to nearest major road; see also McGarva, 2017; UK Biobank, 2023) group included subjects with residential proximity to the nearest major road 50 m or less, noDNMR group included subjects with residential proximity to the nearest major road more than 50 meter. Infs group contained subjects who had one or more ICD10 codes for infectious diseases between January 1, 2006 and January 1, 2016, and noInfs group contained subjects who had not any such code. DNMR_Infs group contained subjects that were presented in both DNMR and Infs; DNMR_noInfs group contained subjects that were presented in both DNMR and noInfs; noDNMR_Infs group contained subjects that were presented in both noDNMR and Infs; noDNMR_noInfs group contained subjects that were presented in both noDNMR and noInfs. AD+ group contained subjects who were diagnosed with AD and/or other dementias after the first occurrence of infection between January 1, 2006 and January 1, 2016, based on ICD10 codes (Supplementary material 1, Alzheimer’s disease and other dementias). Also, we took into account a 5-month latency period between infection and dementia diagnosis.

In order to make difference in age means between groups statistically insignificant, the subjects aged 60–60.5 years were removed from groups DNMR_noInfs and noDNMR_noInfs and updated data is marked by the asterisk in Table 1. These groups are presented in Table 1, which also included those subjects for whom *APOE4* related data was not available. For the cases with *APOE4* carriers and *APOE4* non-carriers, these groups are presented in Supplementary Tables 1, 2, respectively. Note that the number of AD+ cases was small for analyzing men and women separately. Therefore, this study concentrated mostly on data for men and women combined.

**Table 1:** Characteristics of the UK Biobank sample used in this analysis.

Group	Female, age 60–75	Male, age 60–75	Female/male, age 60–75
DNMR Infs	432	542	974
AD+	7	19	26
noAD+	425	523	948
DNMR noInfs*	2,743/2,538*	2,582/2,435*	5,325/4,973*
AD+	17/14*	36/36*	53/50*
noAD+	2,726/2,524*	2,546/2,399*	5,272/4,923*
noDNMR Infs	6,710	7,526	14,236
AD+	91	130	221
noAD+	6,619	7,396	14,015
noDNMR noInfs*	41,184/38,042*	38,323/35,624*	79,507/73,666*
AD+	349/337*	417/408*	766/745*
noAD+	40,835/37,705*	37,906/35,216*	78,741/72,921*
Total	51,069/47,722*	48,973/46,127*	100,042/93,849*
AD+	464/449*	602/593*	1,066/1,042*
noAD+	50,605/47,273*	48,371/45,534*	98,976/92,807*

Shown are the numbers of subjects, who had information about distance to nearest major road (DNMR), and who had history of infections (Infs) or no history of infections (noInfs), between January 1, 2006 and January 1, 2016, and who were diagnosed with AD+ after onset of infection. In this table were also included those subjects for whom *APOE4* related data was not available. Age was calculated at the baseline date January 1, 2006. In order to make difference in age means between DNMR_Infs, DNMR_noInfs, noDNMR_Infs, and noDNMR_noInfs groups statistically insignificant, younger female and male subjects aged 60–60.5 years in DNMR_noInfs and noDNMR_noInfs groups were removed. Numbers after removing are marked by asterisk *.

### 2.2 Analytic approach

Our analytical goal was to evaluate how the interaction between a history of infections and chronically high exposure to TRAP (approximated by DNMR) influences the subsequent risk of AD and other dementias (AD+) in carriers and non-carriers of APOE4 in UKB participants aged 60–75 years. The risk, being defined as the ratio of cases to the number of individuals in the group, and its confidence intervals were estimated using Wilson score interval (Wilson, 1927; Agresti and Coull, 1998). The risk difference and its confidence interval were estimated using Wald interval (Altman et al., 2000; Newcombe, 1998). The risk ratio and its confidence interval were estimated using Wald risk ratio (Jewell, 2004). The risks in groups were compared by employing Fisher’s exact test (Fisher, 1970). The difference in age between groups was estimated using the Welch test (Welch, 1947).

For men and women combined, we utilized the following three data sets: (a) subjects presented in Table 1 (in Table 1 were also included those subjects for whom APOE4 related data was not available), (b) APOE4 carriers (Supplementary Table 1), and (c) APOE4 non-carriers (Supplementary Table 2).

For each data set (a), (b), (c), we considered a set of logistic regression models *risk~Age,dnmr,infs* having linear variables *Age*, *dnmr*, *infs* and their pairwise interactions, having risk of AD+ as a response variable risk and independent variables: *dnmr* = 1 (DNMR<50), *dnmr* = 0 (DNMR> = 50), *infs* = 1 (for subjects with infection history during January 1, 2006 and January 1, 2016), *infs* = 0 (for subjects without infection history during January 1, 2006 and January 1, 2016), and age at the baseline date January 1, 2006 as *Age* variable. The number of all possible logistic regression models equals to 64 for each data set (a), (b), (c) (Supplementary material, Analytic approach 2.1).

The final model selection was based on the Akaike information criterion (AIC) (Akaike, 1973). The optimal, with respect to the minimal AIC criteria, significant results for regression model were found for the regression sets described above. Here, significant regression model means that all regression coefficients were significant (*p*-value<0.05) in a specific model, non-significance means the opposite. R standard software packages (version 4.4.1), along with *glmulti* package (Calcagno, 2022), *brglm2* package (Bias Reduction in Generalized Linear Models, 2025), and *smotefamily* package (Class Imbalance Problem Based on SMOTE, 2025) were utilized. Supplementary material 3 provides additional technical details about these packages and analytic approaches. Supplementary material 4 includes diagnostic plots confirming linearity of Age in the logit and other relevant plots.

## 3 Results

The data was adjusted to align age means in the age distributions in the groups DNMR_Infs, DNMR_noInfs, noDNMR_Infs, and noDNMR_noInfs (Supplementary Tables 5–10). After adjusting data sets, the difference between mean ages in groups was not significant for males, females and both males and females. This ensured that the observed effect in risk of exposure to TRAP and history of infections on AD+ was not due to potentially younger mean age in the groups. Note that only seven female subjects were in the DNMR_Infs group (Table 1). So, we preferred to mostly utilize a data set including both females and males as being more statistically reliable.

**Figure 1:**
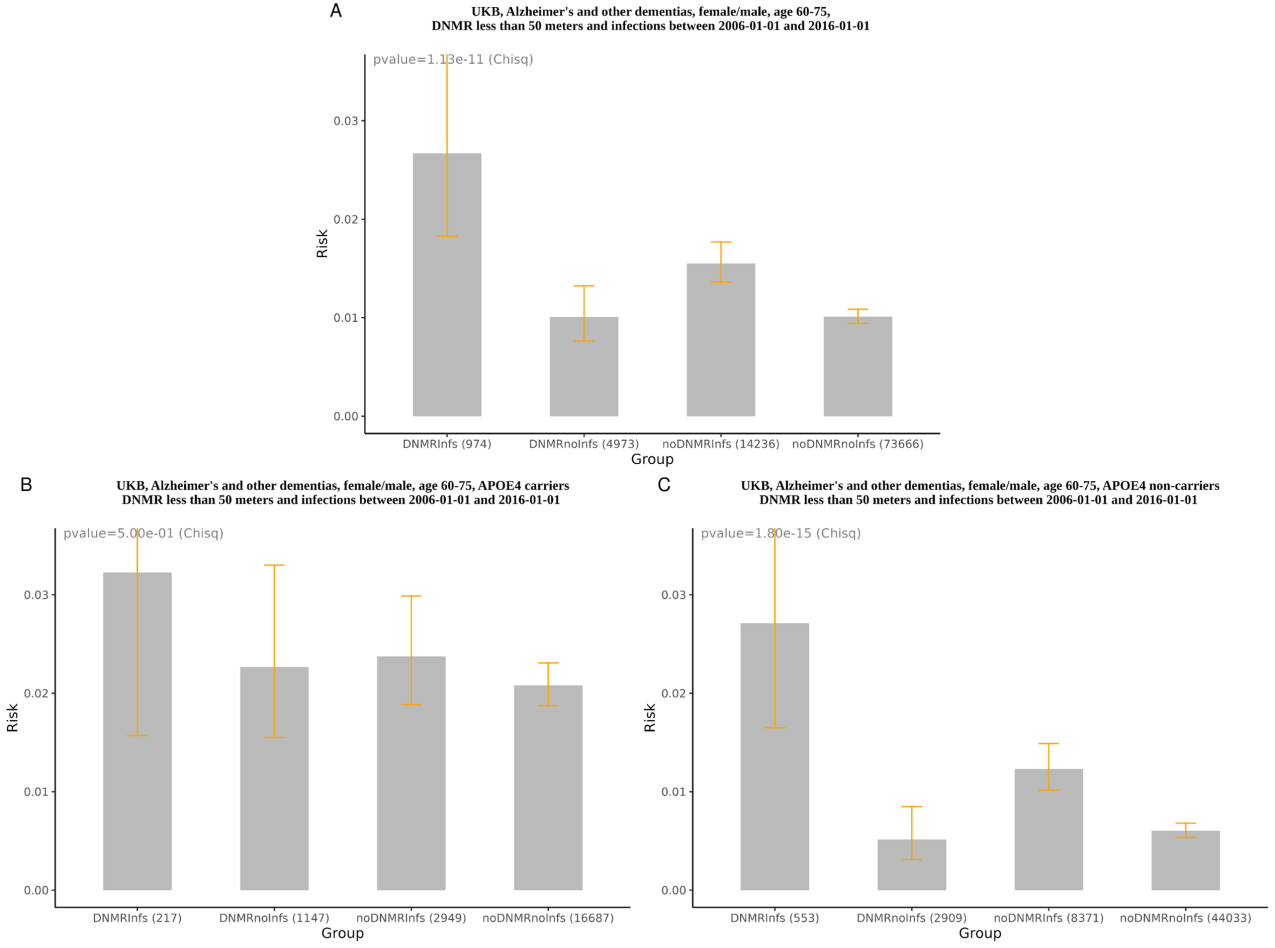
Risk of AD+ (Alzheimer’s disease and other dementias). **(A)** Females/males aged 65–70 years, age—completed age at the time of entry to UKB. DNMR_Infs (risk: mean = 0.0267, 95% CI: 0.0183–0.0388), DNMR_noInfs (risk: mean = 0.0101, 95% CI: 0.0076–0.0132), noDNMR_Infs (risk: mean = 0.0155, 95% CI: 0.0136–0.0177), noDNMR_noInfs (risk: mean = 0.0101, 95% CI: 0.0094–0.0109); **(B)** females/males aged 65–70 years, *APOE4* carriers, age—completed age at the time of entry to UKB. DNMR_Infs (risk: mean = 0.0323, 95% CI: 0.0157–0.0651), DNMR_noInfs (risk: mean = 0.0227, 95% CI: 0.0155–0.0330), noDNMR_Infs (risk: mean = 0.0237, 95% CI: 0.0188–0.0299), noDNMR_noInfs (risk: mean = 0.0208, 95% CI: 0.0187–0.0231); **(C)** females/males aged 65–70 years, *APOE4* non-carriers, age—completed age at the time of entry to UKB. DNMR_Infs (risk: mean = 0.0271, 95% CI: 0.0165–0.0443), DNMR_noInfs (risk: mean = 0.0052, 95% CI: 0.0031–0.0085), noDNMR_Infs (risk: mean = 0.0123, 95% CI: 0.0102–0.0149), noDNMR_noInfs (risk: mean = 0.0060, 95% CI: 0.0054–0.0068). Age was calculated at the baseline date January 1, 2006.

Using pairwise group comparisons (Table 2 and Figure 1A), we found that UKB participants aged 60–75 years with a history of infections and high exposure to TRAP, for men and women combined, had a 164% higher risk of AD+, as compared to individuals of the same age without either risk factor (RR = 2.64 (DNMR_Infs/noDNMR_noInfs), 95% CI [1.79, 3.88]). Separately, infections without TRAP increased the risk of AD+ by 54% (RR = 1.54 (noDNMR_Infs/noDNMR_noInfs), 95% CI [1.32, 1.78]). The impact of TRAP without infections on AD+ was not significant. In non-carriers of *APOE4* (Figure 1C, Supplementary Table 4) with both a history of infections and exposure to TRAP, the relative risk of AD+ was 4.49 (95% CI [2.68, 7.50], risk ratio (DNMR_Infs/noDNMR_noInfs)) compared to subjects without either risk factor. Infections alone (without TRAP) influenced the risk of AD+ less substantially (RR = 2.04 (noDNMR_Infs/noDNMR_noInfs), 95% CI [1.62, 2.55]). The impact of TRAP without infections on AD+ was not significant. In *APOE4* carriers (Figure 1B, Supplementary Table 3), the association between a history of infections and exposure to TRAP with the risk of AD+ was not significant. Table 2 and Supplementary Tables 3, 4 show results of comparisons between all groups.

**Table 2:** Risks of AD+ in different groups of men and women aged 60–75 years.

Test	95% Confidence intervals	Estimate
DNMR_Infs: DNMR_noInfs		
risk DNMR_Infs	[0.0183,0.0388]	0.0267
risk DNMR_noInfs	[0.0076,0.0132]	0.0101
risks difference (DNMR_Infs—DNMR_noInfs)	[0.0061,0.0271]	0.0166
risk ratio (DNMR_Infs/DNMR_noInfs)	[1.6612,4.2433]	2.655
DNMR_Infs: noDNMR_Infs		
risk DNMR_Infs	[0.0183,0.0388]	0.0267
risk noDNMR_Infs	[0.0136,0.0177]	0.0155
risks difference (DNMR_Infs—noDNMR_Infs)	[0.0008,0.0215]	0.0112
risk ratio (DNMR_Infs/noDNMR_Infs)	[1.1513,2.5682]	1.7195
DNMR_Infs: noDNMR_noInfs		
risk DNMR_Infs	[0.0183,0.0388]	0.0267
risk noDNMR_noInfs	[0.0094,0.0109]	0.0101
risks difference (DNMR_Infs—noDNMR_noInfs)	[0.0064,0.0267]	0.0166
risk ratio (DNMR_Infs/noDNMR_noInfs)	[1.7945,3.8825]	2.6395
DNMR_noInfs: noDNMR_Infs		
risk DNMR_noInfs	[0.0076,0.0132]	0.0101
risk noDNMR_Infs	[0.0136,0.0177]	0.0155
risks difference (DNMR_noInfs—noDNMR_Infs)	[−0.0089,-0.0020]	−0.0055
risk ratio (DNMR_noInfs/noDNMR_Infs)	[0.4773,0.8788]	0.6477
DNMR_noInfs: noDNMR_noInfs		
risk DNMR_noInfs	[0.0076,0.0132]	0.0101
risk noDNMR_noInfs	[0.0094,0.0109]	0.0101
risks difference (DNMR_noInfs—noDNMR_noInfs). Therefore, the results of SMOTE analysis showed that the coefficients Age, infs, dnmr for the logistic regression changed about 5% and the coefficient for the term infs*dn	[−0.0029,0.0028]	−0.0001
risk ratio (DNMR_noInfs/noDNMR_noInfs)	[0.7477,1.3219]	0.9942
noDNMR_Infs: noDNMR_noInfs		
risk noDNMR_Infs	[0.0136,0.0177]	0.0155
risk noDNMR_noInfs	[0.0094,0.0109]	0.0101
risks difference (noDNMR_Infs—noDNMR_noInfs)	[0.0033,0.0076]	0.0054
risk ratio (noDNMR_Infs/noDNMR_noInfs)	[1.3225,1.7818]	1.535

For estimating risks, risks differences, and risks ratios, Wilson score interval test, Wald interval test, and Wald risk ratio test were utilized, respectively. For calculations, data in Table 1 marked by the asterisk * were used. ‘Estimate’ column contains the estimates of risk, risk differences, and risk ratio. For example, the estimate of the risk DNMR_Infs (being defined as the ratio of the number of cases to the number of individuals in the group DNMR_Infs) in the first row of ‘DNMR_Infs: DNMR_noInfs’ section equals to 0.0267, that is about 3% of the participants in DNMR_Infs group were diagnosed with AD+. Column ‘95% Confidence Intervals’ contains 95% confidence intervals for the estimates of risk, risk differences, and risk ratio.

The logistic regression analysis confirmed and supplemented the result obtained by means of the groups pairwise comparisons analysis for participants aged 60–75 (Table 3). In particular, going from *dnmr* = 1, *infs* = 1 to *dnmr* = 0, *infs* = 0 corresponds to the comparison between DNMR_Infs and noDNMR_noInfs groups. Let us denote by A the right side of the logistic regression equation using regression coefficients in Table 3: A = −15.489 + 0.171*Age + 0.429*infs + 0.558*dnmr*infs. Then the expression for the risk is as follows: *risk* = 1/(1 + exp (−A)) (Supplementary material, Analytic approach 2.2). Taking into account that the mean age in DNMR_Infs group *meanAge1* = 63.56 years and the mean age in noDNMR_noInfs group *meanAge2* = 63.59 years (Supplementary Tables 5, 6) and setting *Age* = *meanAge1* or *Age* = *meanAge2,* after calculations, we have the risk values in groups DNMR_Infs and noDNMR_noInfs and the relative risk respectively: *risk1* = 0.0257, *risk2* = 0.0098, and *RR* = *risk1*/*risk2* = 2.6265. With a good approximation, these values are close to the corresponding values 0.0267, 0.0101, and 2.6395 presented in Table 2. For *APOE4* carriers and *APOE4* non-carriers cases, the results for the logistic regression analysis are presented in Supplementary Tables 12–15 (more details about regression coefficients are shown in Supplementary material 2 tables).

**Table 3:** Regression coefficients for the best model among all models considered in this study (Supplementary Table 11).

Model/term	Estimate	Std. error	*p*-value
The model, females and males, 60–75			
(Intercept)	−15.489	0.983	<1.00e-20
*Age*	0.171 (1/year)	0.015	<1.00e-20
*infs*	0.429	0.077	2.28e-08
infs*dnmr	0.558	0.210	7.94e-03
The above model with the relevant covariates			
(Intercept)	−15.237	1.030	<1.00e-20
*Age*	0.165 (1/year)	0.016	<1.00e-20
*infs*	0.437	0.079	3.37e-08
infs*dnmr	0.586	0.211	5.52e-03
*education*	−0.157	0.081	5.40e-02
*smoking*	0.017	0.068	8.00e-01
*tsi*1	−0.031	0.108	7.75e-01
*tsi*2	0.029	0.106	7.84e-01
*tsi*3	0.040	0.106	7.02e-01
*tsi*4	0.328	0.099	9.69e-04

Here we presented the result of regression analysis of the set with all possible logistic regression models (64 models) for females/males having linear terms for variables Age, dnmr, infs and their pairwise interactions, having risk of AD+ as a response variable risk and independent variables: dnmr = 1 (DNMR<=50), dnmr = 0 (DNMR>50), infs = 1 (for subjects with history of infections during January 1, 2006 and January 1, 2016), infs = 0 (for subjects without history of infections during January 1, 2006 and January 1, 2016), and age at the baseline date January 1, 2006 as the Age variable. The logistic regression models were evaluated using the Akaike information criterion (AIC). Results for all models that are presented in ascending order by AIC value in Supplementary Table 11. The optimal (best), with respect to the minimal AIC criterion, significant result for regression model was found for the regression set described above and regression coefficients of the best model are presented in this table. For this model, the following covariates were added: education = 1 (for subjects with College or University degree), education = 0 (for subjects without College or University degree), smoker = 1 (if the subject was a smoker), smoker = 0 (if the subject was a non-smoker). For Townsend index, five quintiles were considered: Quintile 1, 0–20%, it represents the least deprived 20% of the population, also known as the most affluent group; Quintile 2, 20–40%, it represents the second-least deprived group; Quintile 3, 40–60%, it represents the middle group in terms of deprivation. Quintile 4, 60–80%, it represents the second-most deprived group; Quintile 5, 80–100%, it represents the most deprived 20% of the population. Four dummy variables tsi1, tsi2, tsi3, and tsi4, with each variable representing one of the Quintiles 2–5 compared to the Quintile 1.

Scientific notation ‘e’ means that the base number is multiplied by 10 raised to the given power. For calculations, data in Table 1 marked by the asterisk * were used.

Note also that the risk difference and the relative risk in groups DNMR_Infs and noDNMR_noInfs increased, respectively, by 92% and by 42% when going from the reference model without interaction (when main factors were independent) to the model with interaction between main factors (Supplementary material, Reference regression model, Supplementary Table 16).

The regression model (Table 3) was assessed for confounding by the covariates related to education, smoking, and the Townsend index (Townsend Deprivation Index). The Townsend index has been the favoured deprivation measure among UK health authorities (Measuring Deprivation, 2002). The following variables were added: *education* = 1 (for subjects with College or University degree), *education* = 0 (for subjects without College or University degree), *smoker =* 1 (if the subject was a smoker)*, smoker =* 0 (if the subject was a non-smoker). For Townsend index, five quintiles were considered: Quintile 1, 0–20%, it represents the least deprived 20% of the population, also known as the most affluent group; Quintile 2, 20–40%, it represents the second-least deprived group; Quintile 3, 40–60%, it represents the middle group in terms of deprivation. Quintile 4, 60–80%, it represents the second-most deprived group; Quintile 5, 80–100%, it represents the most deprived 20% of the population. Four dummy variables *tsi*1, *tsi*2, *tsi*3, and *tsi*4, with each variable representing one of the Quintiles 2–5 compared to the Quintile 1. The regression model was then run using these four dummy variables as predictors, allowing us to analyze the effect of each level on the risk of AD.

The results are presented in Table 3 in ‘Models with relevant covariates’ section. Note that adding *education, smoking, tsi*1, *tsi*2, *tsi*3, and *tsi*4 variables to a regression model, only slightly changed the coefficients of the initial variables of interest. It suggests that the added variables were not significantly related to the terms of our interest *Age*, *infs*, dnmr, infs*dnmr and the regression model (Best model, females and males, 60–75 in Table 3) has been already capturing the essential information.

To further analyze sensitivity and sustainability in our analysis, we applied bias correction technique (Firth, 1993) and the Synthetic Minority Over-sampling Technique (SMOTE) oversampling technique (Chawla et al., 2002). In particular, both techniques are highly effective for handling rare events and balancing data in logistic regression models. The results of the bias correction analysis showed that the coefficients for terms *Age*, *infs*, *dnmr* the logistic regression changed about 5% when applying the bias correction methods (Supplementary material 3, Bias correction for handling rare events in logistic regression). It supported a good level of sensitivity and sustainability in our analysis.

Applying the SMOTE techniques showed how sensitive and sustainable our model and data were to relatively small changes in initial conditions. (Supplementary material 3, Estimate logistic regression coefficients using SMOTE). It allowed to successfully check the absence of potential tangible irregularities in the model and data. Therefore, the SMOTE results also supported a good level of sensitivity and sustainability in our analysis.

Thus, adding covariates, utilizing the bias correction methods, and researching the model behavior due to small perturbations showed that our analysis was robust. Also, the main logistic regression assumptions and the model fit were analyzed and confirmed in Supplementary material 4.

## 4 Discussion

Our study found that a history of infections (regardless of type) is associated with a 54% higher risk of AD+ in UKB participants without high exposure to TRAP. This broadly aligns with our previous findings of the associations between infections and AD risk in the Health and Retirement Study data linked to Medicare records (Ukraintseva et al., 2024). In that research, we demonstrated that infectious diseases of diverse origins (viral, bacterial, fungal) were all associated with increased AD risk (ranging from 16 to 42%). One potential explanation is that weakened immunity may play a more critical role in AD development than any particular microbe, by rendering individuals vulnerable to a broad spectrum of pathogens, which may in turn increase the burden of damage contributing to neurodegeneration. Another possibility is that each infection elicits inflammatory responses that may drive the risk of AD (Whitson et al., 2022).

Exposure to TRAP alone did not significantly impact AD+ risk in our analysis. However, among individuals with both high TRAP exposure and a history of infections, the risk of AD+ was approximately 70% higher than in those with a history of infections alone, suggesting a potential synergy between infections and TRAP. This finding aligns with the ‘multi-hit’ hypothesis of AD, which suggests that the presence of multiple risk factors (‘hits’), especially with a synergy between them, is required for AD to progress to its clinical manifestation (Gong et al., 2018; Patrick et al., 2019; Steele et al., 2022; Lathika Rajendrakumar et al., 2025). Several biological mechanisms could be responsible for the synergy between infections and TRAP: (i) Chronic exposure to TRAP may compromise the integrity of the blood–brain barrier (BBB) and increase the brain’s permeability to pathogens and immune cells activated by them. This can promote neuroinflammation and increase damage burden contributing to neurodegeneration (Perry et al., 2003; Patabendige and Janigro, 2023; Calderón-Garcidueñas et al., 2002, 2015). (ii) Infections can trigger immune response and neuroinflammation via cytokine release (Adamu et al., 2024). Exposure to TRAP could amplify this by activating microglia and astrocytes, prolonging inflammation, and accelerating neuronal damage (Block and Calderón-Garcidueñas, 2009). (iii) Exposure to TRAP, especially to PM2.5 and nitrogen oxides, can generate reactive oxygen species (ROS) that may damage mitochondria (Calderón-Garcidueñas et al., 2002, 2015; Mussalo et al., 2024). This may lead to a deficiency of energy required for a proper response to infections. (iv) Infections may stimulate Aβ production as a part of an antimicrobial defense (Soscia et al., 2010). Exposure to TRAP may further enhance Aβ production, e.g., through increased lipid oxidation (Cacciottolo et al., 2020). These and other possible mechanisms deserve further exploration and confirmation in biomedical research.

A notable finding of our study is that AD+ risk in participants with both a history of infections and high exposure to TRAP, compared to those without either factor, was substantially (349%) higher in *non*-carriers of *APOE4*, but it became non-significant in *APOE4* carriers (Figure 1). One possible explanation could be that *APOE4* is the strongest AD risk factor (besides age), whose AD-promoting effects may mask and outweigh those of the other risk factors. Indeed, the *APOE4* has been linked to numerous AD-promoting features. It can directly contribute to cholesterol transport deficiency in the brain, resulting in poorer myelin synthesis and axon maintenance by oligodendrocytes (Blanchard et al., 2022). This could make neurons more vulnerable to damaging exposures. *APOE4* may compromise BBB integrity (Montagne et al., 2020) and reduce Aβ clearance across the BBB, as well as by microglia, and promote a pro-inflammatory microglial phenotype (Liu et al., 2013; Shi et al., 2017). *APOE4* can also stimulate ROS production and mitochondrial inefficiency, among its many other effects that may facilitate neurodegeneration (Mahley and Huang, 2009; Mahley, 2023).

We recognize several study limitations. In our analysis, we evaluated regression models using the Akaike information criterion. One should note that there is no universal procedure by which one can determine the “best model.” We applied the AIC approach calculating goodness-of-fit and model variability in order to select the most parsimonious regression model (Burnham and Anderson, 2002; Anderson, 2008; Burnham et al., 2011). The AIC approach only gave some rationales behind our analysis but it was neither the main argument, nor the only technical mean for uniquely inferring the shape of regression that we obtained in this study. Another potential limitation could be that the formal statistical association evaluated from regression analysis may not imply actual causality, which should be further studied using causal inference approaches. We also acknowledge that our results reflect association rather than causation, consistent with the observational nature of UKB data. There also remains a possibility that the observed relationship is partially attributable to unmeasured confounders. No occupational exposures (such as industrial pollutants or pathogens) were available for this paper. No primary care data was available too. So, severe infections may be over-represented. An additional limitation is that in the UKB, the participant’s residence distance to the nearest major road (DNMR) was collected only once at the time of entry, so the residential mobility during the follow-up may potentially influence results of the analysis. One more limitation is that the majority of UK Biobank participants (around 94.6%) are white born in the UK. In our study, taking ethnicities into account would result in some groups with only tens of subjects, which would not be enough for statistical analysis. So, ethnicity was not taken into account in our study. Because the number of AD+ events among *APOE4* carriers is relatively limited, we recognize reduced statistical power in this group, which may limit biological interpretation. Due to sample size limitations, additional covariates available in UKB were not incorporated into the analysis. Moreover, while the use of a 50-meter distance to a major road is well supported by previous studies (e.g., Chen et al., 2017), it may still be useful to explore alternative DNMR thresholds (e.g., 75 or 100 meters) in future work. Finally, the UK Biobank is a volunteer-based study and may be prone to a volunteer bias (i.e., participants may be healthier and wealthier than general population), so the results of our analyses might not represent the entire UK population. Generalization of our findings to a more diverse population would require calculating sample weights to correct for the potential healthy volunteer bias. The existing UKB sample weights were, however, not available and applicable to our study because we worked with a selected subsample of the UKB participants. Calculating such weights for this sample is beyond the scope of this short communication.

One should also note that DNMR, which was used as a proxy for a chronically high exposure to TRAP and as an explanatory variable in our analysis, is an indicator of aggregated exposure to all road-related pollutants, not only to those specifically found in car exhaust fumes. Some of the pollutants that are not from car exhaust could be relevant to AD. E.g., a higher intensity traffic has been associated with the higher concentration of airborne fungi in urban air environments. Examples include Alternaria and Cladosporium species which may cause infection and inflammation potentially contributing to neurodegeneration (Muafa et al., 2024; Phuna and Madhavan, 2022; Alonso et al., 2017). The role of exposure to airborne fungi in AD pathology deserves separate investigation, especially in the light of our resent finding suggesting that the impact of recurrent fungal infections on AD risk can be even larger than that of other types of infections, including bacterial and viral (Ukraintseva et al., 2024). Other road-related pollutants, such as noise (The Lancet Regional Health-Europe, 2023), light pollution (Chepesiuk, 2009; Wyse et al., 2011; Aubrecht et al., 2013), and electromagnetic fields (Ahlbom and Feychting, 2003; Kıvrak et al., 2017) may also be relevant to health risks. For instance, noise is currently considered a health problem for citizens of the European Union (European Commission, 2023).

## 5 Conclusion

This study, which involved UK Biobank participants aged 60–75 years, found that chronically high exposure to TRAP significantly exacerbates the detrimental effects of infectious diseases on the risk of AD and other dementias in aging individuals. This finding aligns with the ‘multi-hit’ hypothesis of AD, which implies that the presence of multiple risk factors (‘hits’) is required for AD to progress to clinical onset. The largest relative increase in AD+ risk was seen in participants with a history of infections and exposure to TRAP, who were *non*-carriers of *APOE4* variant. In presence of *APOE4,* the increase in AD+ risk caused by infections and exposure to TRAP became non-significant. One potential explanation for this observation is that *APOE4*, aside from age, is the strongest known risk factor for AD, so its AD-promoting effects outweigh and mask those of other risk factors. Since our results reflect association rather than causation, consistent with the observational nature of UKB data, they require confirmation in future research.

## Data Availability

This study used de-identified data provided by the UK Biobank (https://www.ukbiobank.ac.uk). These data are not freely available to the public but can be accessed upon approval of a data request by the UK Biobank. Specific policies governing the process to access the UK Biobank data can be found online at: https://www.ukbiobank.ac.uk/enable-your-research/apply-for-access.
