# Supplementary material for: Traffic-related air pollution significantly aggravates the detrimental effect of infections on the risk of Alzheimer’s disease and other dementias, especially in non-carriers of APOE4
Source: Front Dement. 2026 Jan 12;4:1668381. doi: 10.3389/frdem.2025.1668381 (PMC12833968; doi:10.3389/frdem.2025.1668381)
Supplement: Supplementary file 1 [file Supplementary_file_1.docx]

Supplementary Material

# Alzheimer’s disease and other dementias, AD+

In this study, Alzheimer’s disease and other dementias (AD+, Alzheimer’s disease plus) group was defined using ICD10 codes as an union of the following groups:

**a) AD (Alzheimer’s disease)**

G30 Alzheimer's disease

G30.0 Alzheimer disease with early onset

G30.1 Alzheimer disease with late onset

G30.8 Other Alzheimer disease

G30.9 Alzheimer disease, unspecified

F00 Dementia in Alzheimer's disease

F00.0 Dementia in Alzheimer's disease with early onset (before age 65)

F00.1 Dementia in Alzheimer's disease with late onset (after age 65)

F00.2 Dementia in Alzheimer's disease, atypical or mixed type

F00.9 Dementia in Alzheimer's disease, unspecified

**b) ADRD (AD-related dementias)**

F01 Vascular dementia

G31.0 Frontotemporal dementia

G31.83 Neurocognitive disorder with Lewy bodies

**c) other dementias and neurodegenerative disorders**

F02 Dementia in other diseases classified elsewhere

F03 unspecified dementia

F05 delirium due to known physiological condition

G31.1 senile degeneration of brain, not elsewhere classified

G31.9 degenerative disease of nervous system, unspecified

Note that a subject belongs to the group AD+ if diagnosed with AD+ condition between January 1, 2006 and January 1, 2016.

# Analytic approach

**2.1**

For men and women combined, we utilized the following three data sets: (a) - subjects presented in Table 1 (in Table 1 were also included those subjects for whom *APOE4* related data was not available), (b) - *APOE4* carriers (Supplementary Table 1), and (c) - *APOE4* non-carriers (Supplementary Table 2). For each data set (a), (b), (c), we considered a set of logistic regression models risk~Age,dnmr,infs having linear variables Age, dnmr, infs and their pairwise interactions) having risk of AD+ as a response variable risk and independent variables: dnmr=1 (DNMR<50), dnmr=0 (DNMR>=50), infs=1 (for subjects with infection history during January 1, 2006 and January 1, 2016), infs=0 (for subjects without infection history during January 1, 2006 and January 1, 2016), and age at the baseline date January 1, 2006 as the Age variable:

ln(*risk*/(1-*risk*)) = Intercept *+ b_1_*Age + b_2_*dnmr + b_3_*infs + b_12_*Age*dnmr + b_13_*Age*infs + b_23_*dnmr*infs*

*…*

ln(*risk*/(1-*risk*)) *=* Intercept*,*

where ln(x) natural logarithm, Intercept is a constant called the bias term (or intercept term), *b_1_, b_2_, b_3_,* *b_12_, b_13_, b_23_* are the regression coefficients corresponding to the *Age*, *dnmr*, *infs*, *Age*dnmr Age*infs, infs*dnmr* terms in the regression model. Only the most complicated model and the simplest are presented in the list above without showing 62 models in between these two models.

Also, expression *risk*/(1-*risk*) and ln(*risk*/(1-*risk*)) are respectively called odds and log odds.

**Note**: the number of all possible such logistic regression models equals to 64 for each data set (a), (b), (c). This is because, this number equals to the number of all possible subsets of a set with six elements (2^6^ = 64 ). Indeed, each regression model uniquely corresponds to some subset of a set with the terms: *Age*, *dnmr*, *infs*, *Age*dnmr Age*infs, infs*dnmr.* The number of the terms equals to 6 (n=6). Therefore, the number of all possible such logistic regression models equals to 2^6^ = 64 for each data set (a), (b), (c). Note that the empty subset, which has 0 terms, corresponds to the regression model with intercept term only.

**2.2**

Let us denote by A the right side of the regression equation. Then the expression for the risk is as follows:

risk = 1/(1+exp(-A))

**2.3**

The regression model (Table 3; Best model, females and males, 60-75) was assessed for confounding by the covariates related to education, smoking, and the Townsend index (Townsend Deprivation Index). The Townsend index has been the favoured deprivation measure among UK health authorities [ ]. The following variables were added: education=1 (for subjects with College or University degree), education=0 (for subjects without College or University degree), smoker=1 (if the subject was a smoker), smoker=0 (if the subject was a non-smoker). For Townsend index, five quintiles were considered: Quintile 1, 0–20%, it represents the least deprived 20% of the population, also known as the most affluent group; Quintile 2 , 20–40%, it represents the second-least deprived group; Quintile 3, 40–60%, it represents the middle group in terms of deprivation. Quintile 4, 60–80%, it represents the second-most deprived group; Quintile 5, 80–100%, it represents the most deprived 20% of the population. Four dummy variables *tsi*1, *tsi*2, *tsi*3, and *tsi*4, with each variable representing one of the Quintiles 2-5 compared to the Quintile 1. The regression model was then run using these four dummy variables as predictors, allowing us to analyze the effect of each level on the risk of AD

Therefore, consider the corresponding model:

ln(*risk*/(1-*risk*)) = Intercept *+ b_1_*Age + b_2_*infs +b_12_*dnmr*infs +*

*c_1_*education + c_2_*smoking +*

*c_3_*tsi*1 *+ c_4_* tsi*2 *+ c_5_* tsi*3 *+ c_6_* tsi*4

where ln(x) natural logarithm, Intercept is a constant called the bias term (or intercept term), *b_1_, b_2_,*  *b_12_, c_1_, c_2_, c_3_, c_4_, c_5_,* and *c_6_* are the regression coefficients corresponding to the *Age*, *infs*, *infs*dnmr, education*, *smoking*, *tsi*1*,*  *tsi*2*,*  *tsi*3*,* and *tsi*4 terms in the regression model.

# Reference regression model

Let us roughly estimate the role of interaction between DNMR and infection by means of introducing a reference regression model (Supplementary Table 16) that didn't include any interactions (when main factors were independent in the model). Denote by B the right side of the logistic regression equation and - using regression coefficients in Supplementary Table 16 - we obtain: B = -15.500 + 0.171**Age* + 0.474**infs* + 0.157**dnmr* and expression for the risk in the reference model *riskref* = 1/(1+exp(-B)) (Supplementary Material, 2.2 Analytic approach). Taking into account that the mean age in DNMR_Infs group *meanAge1*=63.56 years and the mean age in noDNMR_noInfs group *meanAge2*=63.59 years (Supplementary Tables 5, 6) and similar to the calculations in 'Results' section in the main text, we get the risk values in groups DNMR_Infs and noDNMR_noInfs and the relative risk respectively: *riskref1*=0.0180, *riskref2*=0.0097 and RRref= *riskref1*/ *riskref2* = 1.86 for the reference model.

Therefore, the risk effect and relative risk in groups DNMR_Infs and noDNMR_noInfs in the reference model and the model with interaction equal respectively: *riskref1*-*riskref2*= 0.0083, RRref=1.86 and *risk1*-*risk2*=0.0159, RR=2.63.

Thus, the risk difference and the relative risk in groups DNMR_Infs and noDNMR_noInfs increased respectively by 92% = ((*risk1*-*risk2) - (riskref1-riskref2))/(riskref1-riskref2)*  and by 42% = (RR - RRref)/RRref when going from the reference model without interaction (when main factors were independent) to the model with interaction between main factors.

# Supplementary Tables

**Supplementary Table 1**. Characteristics of the UK Biobank sample (*APOE4* carriers) used in this analysis.

| Group | Female, age 60-75 | Male, age 60-75 | Female/Male, age 60-75 |
| --- | --- | --- | --- |
|  |  |  |  |
| DNMR Infs | 98 | 119 | 217 |
| AD+ | 1 | 6 | 7 |
| noAD+ | 97 | 113 | 210 |
|  |  |  |  |
| DNMR noInfs* | 638 / 594* | 580 / 553* | 1218 / 1147* |
| AD+ | 14/12* | 14 | 28 / 26* |
| noAD+ | 624 / 582* | 566 / 539* | 1190 / 1121* |
|  |  |  |  |
| noDNMR Infs | 1382 | 1567 | 2949 |
| AD+ | 27 | 43 | 70 |
| noAD+ | 1355 | 1524 | 2879 |
|  |  |  |  |
| noDNMR noInfs* | 9313 / 8605* | 8684 / 8082* | 17997 / 16687* |
| AD+ | 186 / 178* | 176 / 169* | 362 / 347* |
| noAD+ | 9127 / 8427* | 8508 / 7913* | 17635 / 16340* |
|  |  |  |  |
| Total | 11431 / 10679* | 10950 / 10321* | 22381 / 21000* |
| AD+ | 228 / 218* | 239 / 232* | 467 / 450* |
| noAD+ | 11203 / 10461* | 10711 / 10089* | 21914 / 20550* |

Note: shown are the numbers of subjects, who had DNMR data and who had Infs data with history (Infs) or without history of infections (noInfs) between January 1, 2006 and January 1, 2016 and who were diagnosed with AD+ after onset of infection. Age – age at the baseline date January 1, 2006. In order to make difference in age means between DNMR_Infs, DNMR_noInfs, noDNMR_Infs, and noDNMR_noInfs groups statistically insignificant, younger female and male subjects aged 60-60.5 years in DNMR_noInfs and noDNMR_noInfs groups were removed. Numbers after removing are marked by asterisk *.

**Supplementary Table 2**. Characteristics of the UK Biobank sample (*APOE4* non-carriers) used in this analysis

| Group | Female, age 60-75 | Male, age 60-75 | Female/Male, age 60-75 |
| --- | --- | --- | --- |
|  |  |  |  |
| DNMR Infs | 246 | 307 | 553 |
| AD+ | 6 | 9 | 15 |
| noAD+ | 240 | 298 | 538 |
|  |  |  |  |
| DNMR noInfs* | 1633 / 1508* | 1481 / 1401* | 3114 / 2909* |
| AD+ | 1 | 14 | 15 |
| noAD+ | 1632 / 1507* | 1467 / 1387* | 3099 / 2894* |
|  |  |  |  |
| noDNMR Infs | 4017 | 4354 | 8371 |
| AD+ | 51 | 52 | 103 |
| noAD+ | 3966 | 4302 | 8268 |
|  |  |  |  |
| noDNMR noInfs* | 9313 / 23114* | 22481 / 20919* | 47502 / 44033* |
| AD+ | 110 / 106* | 161 / 160* | 271 / 266* |
| noAD+ | 25021 / 23008* | 22320 / 20759* | 47231 / 43767* |
|  |  |  |  |
| Total | 30917 / 28885* | 28623 / 26981* | 59540 / 55866* |
| AD+ | 168 / 164* | 236 / 235* | 404 / 399* |
| noAD+ | 30749 / 28721* | 28387 / 26746* | 59136 / 55467* |

Note: shown are the numbers of subjects, who had DNMR data and who had Infs data with history (Infs) or without history of infections (noInfs) between January 1, 2006 and January 1, 2016 and who were diagnosed with AD+ after onset of infection . Age – age at the baseline date January 1, 2006. In order to make difference in age means between DNMR_Infs, DNMR_noInfs, noDNMR_Infs, and noDNMR_noInfs groups statistically insignificant, younger female and male subjects aged 60-60.5 years in DNMR_noInfs and noDNMR_noInfs groups were removed. Numbers after removing are marked by asterisk *.

**Supplementary Table 3**. Comparison of the AD+ risks for females/males age 60-75, *APOE4* carriers, between groups.

| Test | 95% Confidence Intervals | Estimate |
| --- | --- | --- |
| **DNMR_Infs : DNMR_noInfs** |  |  |
| risk DNMR_Infs | [0.0157,0.0651] | 0.0323 |
| risk DNMR_noInfs | [0.0155,0.0330] | 0.0227 |
| risks difference (DNMR_Infs - DNMR_noInfs) | [-0.0154,0.0346] | 0.0096 |
| risk ratio (DNMR_Infs / DNMR_noInfs) | [0.6256,3.2372] | 1.4231 |
| **DNMR_Infs : noDNMR_Infs** |  |  |
| risk DNMR_Infs | [0.0157,0.0651] | 0.0323 |
| risk noDNMR_Infs | [0.0188,0.0299] | 0.0237 |
| risks difference (DNMR_Infs - noDNMR_Infs) | [-0.0156,0.0327] | 0.0085 |
| risk ratio (DNMR_Infs / noDNMR_Infs) | [0.6326,2.9194] | 1.359 |
| **DNMR_Infs : noDNMR_noInfs** |  |  |
| risk DNMR_Infs | [0.0157,0.0651] | 0.0323 |
| risk noDNMR_noInfs | [0.0187,0.0231] | 0.0208 |
| risks difference (DNMR_Infs - noDNMR_noInfs) | [-0.0121,0.0351] | 0.0115 |
| risk ratio (DNMR_Infs / noDNMR_noInfs) | [0.7430,3.2389] | 1.5513 |
| **DNMR_noInfs : noDNMR_Infs** |  |  |
| risk DNMR_noInfs | [0.0155,0.0330] | 0.0227 |
| risk noDNMR_Infs | [0.0188,0.0299] | 0.0237 |
| risks difference (DNMR_noInfs - noDNMR_Infs) | [-0.0113,0.0091] | -0.0011 |
| risk ratio (DNMR_noInfs / noDNMR_Infs) | [0.6120,1.4901] | 0.955 |
| **DNMR_noInfs : noDNMR_noInfs** |  |  |
| risk DNMR_noInfs | [0.0155,0.0330] | 0.0227 |
| risk noDNMR_noInfs | [0.0187,0.0231] | 0.0208 |
| risks difference (DNMR_noInfs - noDNMR_noInfs) | [-0.0070,0.0108] | 0.0019 |
| risk ratio (DNMR_noInfs / noDNMR_noInfs) | [0.7351,1.6165] | 1.0901 |
| **noDNMR_Infs : noDNMR_noInfs** |  |  |
| risk noDNMR_Infs | [0.0188,0.0299] | 0.0237 |
| risk noDNMR_noInfs | [0.0187,0.0231] | 0.0208 |
| risks difference (noDNMR_Infs - noDNMR_noInfs) | [-0.0030,0.0088] | 0.0029 |
| risk ratio (noDNMR_Infs / noDNMR_noInfs) | [0.8856,1.4713] | 1.1415 |

Note: for estimating risks, risks differences, and risks ratios, Wilson score interval test, Wald interval test, and Wald risk ratio test were utilized respectively. For calculations, data in Supplementary Table 1 marked by the asterisk * were used. ‘Estimate’ column contains the estimates of risk, risk differences, and risk ratio. For example, the estimate of the risk DNMR_Infs (being defined as the ratio of the number of cases to the number of individuals in the group DNMR_Infs) in the first row of ‘DNMR_Infs : DNMR_noInfs’ section equals to 0.0323, that is about 3.2% of the participants in DNMR_Infs group were diagnosed with AD+. Column ‘95% Confidence Intervals’ contains 95% confidence intervals for the estimates of risk, risk differences, and risk ratio.

**Supplementary Table 4**. Comparison of the AD+ risks for females/males age 60-75, *APOE4* non-carriers, between groups.

| Test | 95% Confidence Intervals | Estimate |
| --- | --- | --- |
| **DNMR_Infs : DNMR_noInfs** |  |  |
| risk DNMR_Infs | [0.0165,0.0443] | 0.0271 |
| risk DNMR_noInfs | [0.0031,0.0085] | 0.0052 |
| risks difference (DNMR_Infs - DNMR_noInfs) | [0.0082,0.0358] | 0.0220 |
| risk ratio (DNMR_Infs / DNMR_noInfs) | [2.5866,10.6983] | 5.2604 |
| **DNMR_Infs : noDNMR_Infs** |  |  |
| risk DNMR_Infs | [0.0165,0.0443] | 0.0271 |
| risk noDNMR_Infs | [0.0102,0.0149] | 0.0123 |
| risks difference (DNMR_Infs - noDNMR_Infs) | [0.0011,0.0286] | 0.0148 |
| risk ratio (DNMR_Infs / noDNMR_Infs) | [1.2914,3.7632] | 2.2045 |
| **DNMR_Infs : noDNMR_noInfs** |  |  |
| risk DNMR_Infs | [0.0165,0.0443] | 0.0271 |
| risk noDNMR_noInfs | [0.0054,0.0068] | 0.0060 |
| risks difference (DNMR_Infs - noDNMR_noInfs) | [0.0075,0.0346] | 0.0211 |
| risk ratio (DNMR_Infs / noDNMR_noInfs) | [2.6874,7.5024] | 4.4902 |
| **DNMR_noInfs : noDNMR_Infs** |  |  |
| risk DNMR_noInfs | [0.0031,0.0085] | 0.0052 |
| risk noDNMR_Infs | [0.0102,0.0149] | 0.0123 |
| risks difference (DNMR_noInfs - noDNMR_Infs) | [-0.0107,-0.0036] | -0.0071 |
| risk ratio (DNMR_noInfs / noDNMR_Infs) | [0.2442,0.7191] | 0.4191 |
| **DNMR_noInfs : noDNMR_noInfs** |  |  |
| risk DNMR_noInfs | [0.0031,0.0085] | 0.0052 |
| risk noDNMR_noInfs | [0.0054,0.0068] | 0.0060 |
| risks difference (DNMR_noInfs - noDNMR_noInfs) | [-0.0036,0.0018] | -0.0009 |
| risk ratio (DNMR_noInfs / noDNMR_noInfs) | [0.5081,1.4340] | 0.8536 |
| **noDNMR_Infs : noDNMR_noInfs** |  |  |
| risk noDNMR_Infs | [0.0102,0.0149] | 0.0123 |
| risk noDNMR_noInfs | [0.0054,0.0068] | 0.0060 |
| risks difference (noDNMR_Infs - noDNMR_noInfs) | [0.0038,0.0087] | 0.0063 |
| risk ratio (noDNMR_Infs / noDNMR_noInfs) | [1.6244,2.5540] | 2.0368 |

Note: for estimating risks, risks differences, and risks ratios, Wilson score interval test, Wald interval test, and Wald risk ratio test were utilized respectively. For calculations, data in Supplementary Table 2 marked by the asterisk * were used. ‘Estimate’ column contains the estimates of risk, risk differences, and risk ratio. For example, the estimate of the risk DNMR_Infs (being defined as the ratio of the number of cases to the number of individuals in the group DNMR_Infs) in the first row of ‘DNMR_Infs : DNMR_noInfs’ section equals to 0.0271, that is about 2.7% of the participants in DNMR_Infs group were diagnosed with AD+. Column ‘95% Confidence Intervals’ contains 95% confidence intervals for the estimates of risk, risk differences, and risk ratio.

**Supplementary Table 5.** Age distributions for females/males aged 60-75 in the groups shown in the Group column. Age was recorded at the baseline date January 1, 2006.

| Group | min | max | mean | sd | number |
| --- | --- | --- | --- | --- | --- |
| **Females/Males, age 60-75** |  |  |  |  |  |
| DNMR_Infs | 60.05 | 68.63 | 63.56 | 2.10 | 974 |
| DNMR_noInfs | 60.55 | 69.55 | 63.56 | 1.96 | 4973 |
| noDNMR_Infs | 60.05 | 71.80 | 63.57 | 2.12 | 14236 |
| noDNMR_noInfs | 60.55 | 69.63 | 63.59 | 1.96 | 73666 |

Note: for calculations in this table, data in Supplementary Table 1 marked by the asterisk were used.

**Supplementary Table 6.** Welch Two Sample t-test for two age distributions between groups for females/males aged 60-75 years. The groups that were compared shown in the Group column. Age was recorded at the time of the visit.

| Test | P-value | 95% Confidence Interval | Estimate | stderr |
| --- | --- | --- | --- | --- |
| **Females/Males, age 60-75** |  |  |  |  |
| DNMR_Infs : DNMR_noInfs | 9.66e-01 | [-0.14,0.15] | 63.56 : 63.56 | 0.07 |
| DNMR_Infs : noDNMR_Infs | 8.47e-01 | [-0.15,0.12] | 63.56 : 63.57 | 0.07 |
| DNMR_Infs : noDNMR_noInfs | 6.84e-01 | [-0.16,0.11] | 63.56 : 63.59 | 0.07 |
| DNMR_noInfs : noDNMR_Infs | 6.20e-01 | [-0.08,0.05] | 63.56 : 63.57 | 0.03 |
| DNMR_noInfs : noDNMR_noInfs | 2.84e-01 | [-0.09,0.03] | 63.56 : 63.59 | 0.03 |
| noDNMR_Infs : noDNMR_noInfs | 4.61e-01 | [-0.05,0.02] | 63.57 : 63.59 | 0.02 |

Note: for calculations in this table, data in Supplementary Table 1 marked by the asterisk were used. In Estimate column, the colon “:” separates the mean ages in the corresponding groups. For example, 63.56 : 63.56 in the second row and Estimate column means that mean age in DNMR_Infs group equals 63.56 years and mean age in noDNMR_Infs group equals 63.57 years.

**Supplementary Table 7.** Age distributions for females/males, *APOE4* carriers, aged 60-75 in the groups shown in the Group column. Age was recorded at the baseline date January 1, 2006.

| Group | min | max | mean | sd | number |
| --- | --- | --- | --- | --- | --- |
| **Females/Males, age 60-75** |  |  |  |  |  |
| DNMR_Infs | 60.05 | 68.38 | 63.41 | 2.07 | 217 |
| DNMR_noInfs | 60.55 | 68.63 | 63.46 | 1.95 | 1147 |
| noDNMR_Infs | 60.05 | 69.80 | 63.50 | 2.13 | 2949 |
| noDNMR_noInfs | 60.55 | 68.96 | 63.57 | 1.95 | 16687 |

Note: for calculations in this table, data in Supplementary Table 1 marked by the asterisk were used.

**Supplementary Table 8.** Welch Two Sample t-test for two age distributions between groups for females/males, *APOE4* carriers, aged 60-75 years. The groups that were compared shown in the Group column. Age was recorded at the time of the visit.

| Test | P-value | 95% Confidence Interval | Estimate | stderr |
| --- | --- | --- | --- | --- |
| **Females/Males, age 60-75** |  |  |  |  |
| DNMR_Infs : DNMR_noInfs | 7.65-01 | [-0.34,0.25] | 63.41 : 63.46 | 0.77 |
| DNMR_Infs : noDNMR_Infs | 5.35e-01 | [-0.38,0.20] | 63.41 : 63.51 | 0.15 |
| DNMR_Infs : noDNMR_noInfs | 2.80e-01 | [-0.43,0.13] | 63.41 : 63.57 | 0.14 |
| DNMR_noInfs : noDNMR_Infs | 5.17e-01 | [-0.18,0.09] | 63.46 : 63.51 | 0.07 |
| DNMR_noInfs : noDNMR_noInfs | 7.04e-02 | [-0.225,0.009] | 63.46 : 63.57 | 0.06 |
| noDNMR_Infs : noDNMR_noInfs | 1.36e-01 | [-0.14,0.02] | 63.51 : 63.57 | 0.04 |

Note: for calculations in this table, data in Supplementary Table 2 marked by the asterisk were used. In Estimate column, the colon “:” separates the mean ages in the corresponding groups. For example, 63.41 : 63.51 in the second row and Estimate column means that mean age in DNMR_Infs group equals 63. 41 years and mean age in noDNMR_Infs group equals 63.51 years.

**Supplementary Table 9.** Age distributions for females/males, *APOE4* non-carriers, aged 60-75 in the groups shown in the Group column. Age was recorded at the baseline date January 1, 2006.

| Group | min | max | mean | sd | number |
| --- | --- | --- | --- | --- | --- |
| **Females/Males, age 60-75** |  |  |  |  |  |
| DNMR_Infs | 60.05 | 68.38 | 63.61 | 2.12 | 553 |
| DNMR_noInfs | 60.55 | 68.71 | 63.60 | 1.96 | 2909 |
| noDNMR_Infs | 60.05 | 71.80 | 63.57 | 2.10 | 8371 |
| noDNMR_noInfs | 60.55 | 69.13 | 63.60 | 1.96 | 44033 |

Note: for calculations in this table, data in Supplementary Table 2 marked by the asterisk were used.

**Supplementary Table 10.** Welch Two Sample t-test for two age distributions between groups for females/males, *APOE4* non-carriers, aged 60-75 years. The groups that were compared shown in the Group column. Age was recorded at the time of the visit.

| Test | P-value | 95% Confidence Interval | Estimate | stderr |
| --- | --- | --- | --- | --- |
| **Females/Males, age 60-75** |  |  |  |  |
| DNMR_Infs : DNMR_noInfs | 8.81-01 | [-0.17,0.21] | 63.61 : 63.60 | 0.10 |
| DNMR_Infs : noDNMR_Infs | 6.73e-01 | [-0.14,0.22] | 63.61 : 63.57 | 0.09 |
| DNMR_Infs : noDNMR_noInfs | 9.02e-01 | [-0.17,0.19] | 63.61 : 63.60 | 0.09 |
| DNMR_noInfs : noDNMR_Infs | 5.65e-01 | [-0.06,0.11] | 63.60 : 63.57 | 0.04 |
| DNMR_noInfs : noDNMR_noInfs | 9.27e-01 | [-0.08,0.07] | 63.60 : 63.60 | 0.04 |
| noDNMR_Infs : noDNMR_noInfs | 2.57e-01 | [-0.08,0.02] | 63.57 : 63.60 | 0.02 |

Note: for calculations in this table, data in Supplementary Table 2 marked by the asterisk used. In Estimate column, the colon “:” separates the mean ages in the corresponding groups. For example, 63.61 : 63.56 in the second row and Estimate column means that mean age in DNMR_Infs group equals 63.61 years and mean age in noDNMR_Infs group equals 63.57 years.

**Supplementary Table 11.** Logistic regression models, females/males aged 60-75 years.

| Regression Model | AIC | Signf |
| --- | --- | --- |
| **females/males, age 60-75** |  |  |
| *risk* ~ 1 + *Age* + *infs* + *infs*:*Age* + *infs*:*dnmr* | 11289.06 | 0 |
| *risk* ~ 1 + *Age* + *infs* + *infs*:*dnmr* | 11289.84 | 1 |
| *risk* ~ 1 + *Age* + *infs*:*Age* + *infs*:*dnmr* | 11290.51 | 1 |
| *risk* ~ 1 + *Age* + *infs* + *dnmr*:*Age* + *infs*:*Age* + *infs*:*dnmr* | 11291.06 | 0 |
| *risk* ~ 1 + *Age* + *dnmr* + *infs* + *infs*:*Age* + *infs*:*dnmr* | 11291.06 | 0 |
| *risk* ~ 1 + *Age* + *infs* + *dnmr*:*Age* + *infs*:*dnmr* | 11291.84 | 0 |
| *risk* ~ 1 + *Age* + *dnmr* + *infs* + *infs*:*dnmr* | 11291.84 | 0 |
| *risk* ~ 1 + *Age* + *dnmr*:*Age* + *infs*:*Age* + *infs*:*dnmr* | 11292.51 | 0 |
| *risk* ~ 1 + *Age* + *dnmr* + *infs*:*Age* + *infs*:*dnmr* | 11292.51 | 0 |
| *risk* ~ 1 + *Age* + *dnmr* + *infs* + *dnmr*:*Age* + *infs*:*Age* + *infs*:*dnmr* | 11292.99 | 0 |
| *risk* ~ 1 + *Age* + *infs* + *infs*:*Age* | 11293.17 | 0 |
| *risk* ~ 1 + *Age* + *dnmr* + *infs* + *infs*:*Age* | 11293.53 | 0 |
| *risk* ~ 1 + *Age* + *infs* + *dnmr*:*Age* + *infs*:*Age* | 11293.56 | 0 |
| *risk* ~ 1 + *Age* + *dnmr* + *infs* + *dnmr*:*Age* + *infs*:*dnmr* | 11293.70 | 0 |
| *risk* ~ 1 + *Age* + *infs* | 11293.98 | 1 |
| *risk* ~ 1 + *Age* + *dnmr* + *infs* | 11294.34 | 0 |
| *risk* ~ 1 + *Age* + *dnmr* + *dnmr*:*Age* + *infs*:*Age* + *infs*:*dnmr* | 11294.36 | 0 |
| *risk* ~ 1 + *Age* + *infs* + *dnmr*:*Age* | 11294.37 | 0 |
| *risk* ~ 1 + *Age* + *infs*:*Age* | 11294.73 | 1 |
| *risk* ~ 1 + *Age* + *dnmr* + *infs*:*Age* | 11295.09 | 0 |
| *risk* ~ 1 + *Age* + *dnmr*:*Age* + *infs*:*Age* | 11295.12 | 0 |
| *risk* ~ 1 + *Age* + *dnmr* + *infs* + *dnmr*:*Age* + *infs*:*Age* | 11295.41 | 0 |
| *risk* ~ 1 + *Age* + *dnmr* + *infs* + *dnmr*:*Age* | 11296.22 | 0 |
| *risk* ~ 1 + *Age* + *dnmr* + *dnmr*:*Age* + *infs*:*Age* | 11296.97 | 0 |
| *risk* ~ 1 + *Age* + *infs*:*dnmr* | 11316.64 | 1 |
| *risk* ~ 1 + *Age* + *dnmr*:*Age* + *infs*:*dnmr* | 11318.29 | 0 |
| *risk* ~ 1 + *Age* + *dnmr* + *infs*:*dnmr* | 11318.30 | 0 |
| *risk* ~ 1 + *Age* + *dnmr* + *dnmr*:*Age* + *infs*:*dnmr* | 11320.15 | 0 |
| *risk* ~ 1 + *Age* | 11330.32 | 1 |
| *risk* ~ 1 + *Age* + *dnmr* | 11330.66 | 0 |
| *risk* ~ 1 + *Age* + *dnmr*:*Age* | 11330.69 | 0 |
| *risk* ~ 1 + *Age* + *dnmr* + *dnmr*:*Age* | 11332.54 | 0 |
| *risk* ~ 1 + *infs* + *infs*:*Age* + *infs*:*dnmr* | 11396.15 | 1 |
| *risk* ~ 1 + *dnmr* + *infs* + *dnmr*:*Age* + *infs*:*Age* + *infs*:*dnmr* | 11396.64 | 0 |
| *risk* ~ 1 + *dnmr* + *infs* + *infs*:*Age* + *infs*:*dnmr* | 11398.15 | 0 |
| *risk* ~ 1 + *infs* + *dnmr*:*Age* + *infs*:*Age* + *infs*:*dnmr* | 11398.15 | 0 |
| *risk* ~ 1 + *dnmr* + *infs* + *dnmr*:*Age* + *infs*:*Age* | 11398.29 | 1 |
| *risk* ~ 1 + *infs* + *infs*:*Age* | 11400.25 | 1 |
| *risk* ~ 1 + *infs* + *dnmr*:*Age* + *infs*:*Age* | 11400.53 | 0 |
| *risk* ~ 1 + *dnmr* + *infs* + *infs*:*Age* | 11400.71 | 0 |
| *risk* ~ 1 + *dnmr* + *dnmr*:*Age* + *infs*:*Age* + *infs*:*dnmr* | 11407.48 | 1 |
| *risk* ~ 1 + *dnmr* + *infs* + *dnmr*:*Age* + *infs*:*dnmr* | 11409.14 | 1 |
| *risk* ~ 1 + *dnmr* + *dnmr*:*Age* + *infs*:*Age* | 11409.68 | 1 |
| *risk* ~ 1 + *infs*:*Age* + *infs*:*dnmr* | 11410.29 | 1 |
| *risk* ~ 1 + *dnmr* + *infs* + *dnmr*:*Age* | 11411.58 | 1 |
| *risk* ~ 1 + *infs* + *infs*:*dnmr* | 11412.20 | 1 |
| *risk* ~ 1 + *dnmr*:*Age* + *infs*:*Age* + *infs*:*dnmr* | 11412.29 | 0 |
| *risk* ~ 1 + *dnmr* + *infs*:*Age* + *infs*:*dnmr* | 11412.29 | 0 |
| *risk* ~ 1 + *infs*:*Age* | 11414.15 | 1 |
| *risk* ~ 1 + *infs* + *dnmr*:*Age* + *infs*:*dnmr* | 11414.19 | 0 |
| *risk* ~ 1 + *dnmr* + *infs* + *infs*:*dnmr* | 11414.20 | 0 |
| *risk* ~ 1 + *dnmr*:*Age* + *infs*:*Age* | 11414.39 | 0 |
| *risk* ~ 1 + *dnmr* + *infs*:*Age* | 11414.62 | 0 |
| *risk* ~ 1 + *infs* | 11416.25 | 1 |
| *risk* ~ 1 + *infs* + *dnmr*:*Age* | 11416.48 | 0 |
| *risk* ~ 1 + *dnmr* + *infs* | 11416.71 | 0 |
| *risk* ~ 1 + *dnmr* + *dnmr*:*Age* + *infs*:*dnmr* | 11436.37 | 1 |
| *risk* ~ 1 + *infs*:*dnmr* | 11439.81 | 1 |
| *risk* ~ 1 + *dnmr* + *infs*:*dnmr* | 11441.42 | 0 |
| *risk* ~ 1 + *dnmr*:*Age* + *infs*:*dnmr* | 11441.54 | 0 |
| *risk* ~ 1 + *dnmr* + *dnmr*:*Age* | 11448.77 | 1 |
| *risk* ~ 1 | 11453.52 | 1 |
| *risk* ~ 1 + *dnmr*:*Age* | 11453.74 | 0 |
| *risk* ~ 1 + *dnmr* | 11453.97 | 0 |

**Note:** Response variable *risk* is risk of AD+. Independent variables: *dnmr*=1 (DNMR<50), *dnmr*=0 (DNMR>=50), *infs*=1 (for subjects with prior infection history during January 1, 2006 and January 1, 2016), *infs*=0 (for subjects without prior infection history during January 1, 2006 and January 1, 2016), and age at the baseline date January 1, 2006 as the *Age* variable. A logistic regression set, having 64 models with linear terms and their pairwise interactions and corresponding to females/ males aged 60-75 were analyzed and presented in ascending order by AIC value. Signf=1 means that all regression coefficient were significant (P-value<0.05) in a specific model, Signf=0 means the opposite. For regression model a short notation used. For instance, *risk* ~ 1 + *Age* + *dnmr* + *infs* + *dnmr***Age* + *infs***dnmr* denotes a standard logistic regression equation ln(*risk*/(1-*risk*)) = Intercept + b_1_**Age* + b_2_**dnmr* + b_3_**infs* + b_12_**Age***dnmr* + b_31_**infs***dnmr* where ln(x) natural logarithm, log, Intercept is a constant called the bias term (or intercept term), b_1_, b_2_, b_3_, b_12_, b_31_ are the regression coefficients corresponding to the *Age*, *dnmr*, *infs*, *Age***dnmr*, *infs***dnmr* terms in the regression model.

**Supplementary Table 12.** Logistic regression models, females/males aged 60-75 years, *APOE4* carriers.

| Regression Model | AIC | Signf |
| --- | --- | --- |
| **females/males,** *APOE4* **carriers, age 60-75** |  |  |
| *risk* ~ 1 + *Age* + *infs* + *infs*:*Age* + *infs*:*dnmr* | 11289.50 | 0 |
| *risk* ~ 1 + *Age* + *infs* + *infs*:*dnmr* | 11290.28 | 1 |
| *risk* ~ 1 + *Age* + *infs*:*Age* + *infs*:*dnmr* | 11290.94 | 1 |
| *risk* ~ 1 + *Age* + *infs* + *dnmr*:*Age* + *infs*:*Age* + *infs*:*dnmr* | 11291.50 | 0 |
| *risk* ~ 1 + *Age* + *dnmr* + *infs* + *infs*:*Age* + *infs*:*dnmr* | 11291.50 | 0 |
| *risk* ~ 1 + *Age* + *infs* + *dnmr*:*Age* + *infs*:*dnmr* | 11292.27 | 0 |
| *risk* ~ 1 + *Age* + *dnmr* + *infs* + *infs*:*dnmr* | 11292.27 | 0 |
| *risk* ~ 1 + *Age* + *dnmr*:*Age* + *infs*:*Age* + *infs*:*dnmr* | 11292.94 | 0 |
| *risk* ~ 1 + *Age* + *dnmr* + *infs*:*Age* + *infs*:*dnmr* | 11292.94 | 0 |
| *risk* ~ 1 + *Age* + *dnmr* + *infs* + *dnmr*:*Age* + *infs*:*Age* + *infs*:*dnmr* | 11293.43 | 0 |
| *risk* ~ 1 + *Age* + *infs* + *infs*:*Age* | 11293.61 | 0 |
| *risk* ~ 1 + *Age* + *dnmr* + *infs* + *infs*:*Age* | 11293.98 | 0 |
| *risk* ~ 1 + *Age* + *infs* + *dnmr*:*Age* + *infs*:*Age* | 11294.01 | 0 |
| *risk* ~ 1 + *Age* + *dnmr* + *infs* + *dnmr*:*Age* + *infs*:*dnmr* | 11294.13 | 0 |
| *risk* ~ 1 + *Age* + *infs* | 11294.42 | 1 |
| *risk* ~ 1 + *Age* + *dnmr* + *infs* | 11294.78 | 0 |
| *risk* ~ 1 + *Age* + *dnmr* + *dnmr*:*Age* + *infs*:*Age* + *infs*:*dnmr* | 11294.79 | 0 |
| *risk* ~ 1 + *Age* + *infs* + *dnmr*:*Age* | 11294.81 | 0 |
| *risk* ~ 1 + *Age* + *infs*:*Age* | 11295.16 | 1 |
| *risk* ~ 1 + *Age* + *dnmr* + *infs*:*Age* | 11295.53 | 0 |
| *risk* ~ 1 + *Age* + *dnmr*:*Age* + *infs*:*Age* | 11295.56 | 0 |
| *risk* ~ 1 + *Age* + *dnmr* + *infs* + *dnmr*:*Age* + *infs*:*Age* | 11295.86 | 0 |
| *risk* ~ 1 + *Age* + *dnmr* + *infs* + *dnmr*:*Age* | 11296.66 | 0 |
| *risk* ~ 1 + *Age* + *dnmr* + *dnmr*:*Age* + *infs*:*Age* | 11297.41 | 0 |
| *risk* ~ 1 + *Age* + *infs*:*dnmr* | 11317.07 | 1 |
| *risk* ~ 1 + *Age* + *dnmr*:*Age* + *infs*:*dnmr* | 11318.71 | 0 |
| *risk* ~ 1 + *Age* + *dnmr* + *infs*:*dnmr* | 11318.73 | 0 |
| *risk* ~ 1 + *Age* + *dnmr* + *dnmr*:*Age* + *infs*:*dnmr* | 11320.57 | 0 |
| *risk* ~ 1 + *Age* | 11330.76 | 1 |
| *risk* ~ 1 + *Age* + *dnmr* | 11331.10 | 0 |
| *risk* ~ 1 + *Age* + *dnmr*:*Age* | 11331.13 | 0 |
| *risk* ~ 1 + *Age* + *dnmr* + *dnmr*:*Age* | 11332.98 | 0 |
| *risk* ~ 1 + *infs* + *infs*:*Age* + *infs*:*dnmr* | 11396.59 | 1 |
| *risk* ~ 1 + *dnmr* + *infs* + *dnmr*:*Age* + *infs*:*Age* + *infs*:*dnmr* | 11397.08 | 0 |
| *risk* ~ 1 + *dnmr* + *infs* + *infs*:*Age* + *infs*:*dnmr* | 11398.59 | 0 |
| *risk* ~ 1 + *infs* + *dnmr*:*Age* + *infs*:*Age* + *infs*:*dnmr* | 11398.59 | 0 |
| *risk* ~ 1 + *dnmr* + *infs* + *dnmr*:*Age* + *infs*:*Age* | 11398.74 | 1 |
| *risk* ~ 1 + *infs* + *infs*:*Age* | 11400.70 | 1 |
| *risk* ~ 1 + *infs* + *dnmr*:*Age* + *infs*:*Age* | 11400.98 | 0 |
| *risk* ~ 1 + *dnmr* + *infs* + *infs*:*Age* | 11401.16 | 0 |
| *risk* ~ 1 + *dnmr* + *dnmr*:*Age* + *infs*:*Age* + *infs*:*dnmr* | 11407.93 | 1 |
| *risk* ~ 1 + *dnmr* + *infs* + *dnmr*:*Age* + *infs*:*dnmr* | 11409.60 | 1 |
| *risk* ~ 1 + *dnmr* + *dnmr*:*Age* + *infs*:*Age* | 11410.14 | 1 |
| *risk* ~ 1 + *infs*:*Age* + *infs*:*dnmr* | 11410.74 | 1 |
| *risk* ~ 1 + *dnmr* + *infs* + *dnmr*:*Age* | 11412.04 | 1 |
| *risk* ~ 1 + *infs* + *infs*:*dnmr* | 11412.65 | 1 |
| *risk* ~ 1 + *dnmr*:*Age* + *infs*:*Age* + *infs*:*dnmr* | 11412.74 | 0 |
| *risk* ~ 1 + *dnmr* + *infs*:*Age* + *infs*:*dnmr* | 11412.74 | 0 |
| *risk* ~ 1 + *infs*:*Age* | 11414.61 | 1 |
| *risk* ~ 1 + *infs* + *dnmr*:*Age* + *infs*:*dnmr* | 11414.64 | 0 |
| *risk* ~ 1 + *dnmr* + *infs* + *infs*:*dnmr* | 11414.65 | 0 |
| *risk* ~ 1 + *dnmr*:*Age* + *infs*:*Age* | 11414.85 | 0 |
| *risk* ~ 1 + *dnmr* + *infs*:*Age* | 11415.08 | 0 |
| *risk* ~ 1 + *infs* | 11416.70 | 1 |
| *risk* ~ 1 + *infs* + *dnmr*:*Age* | 11416.94 | 0 |
| *risk* ~ 1 + *dnmr* + *infs* | 11417.17 | 0 |
| *risk* ~ 1 + *dnmr* + *dnmr*:*Age* + *infs*:*dnmr* | 11436.81 | 1 |
| *risk* ~ 1 + *infs*:*dnmr* | 11440.25 | 1 |
| *risk* ~ 1 + *dnmr* + *infs*:*dnmr* | 11441.86 | 0 |
| *risk* ~ 1 + *dnmr*:*Age* + *infs*:*dnmr* | 11441.98 | 0 |
| *risk* ~ 1 + *dnmr* + *dnmr*:*Age* | 11449.22 | 1 |
| *risk* ~ 1 | 11453.97 | 1 |
| *risk* ~ 1 + *dnmr*:*Age* | 11454.19 | 0 |
| *risk* ~ 1 + *dnmr* | 11454.42 | 0 |

**Note:** Response variable *risk* is risk of AD+. Independent variables: *dnmr*=1 (DNMR<50), *dnmr*=0 (DNMR>=50), *infs*=1 (for subjects with prior infection history during January 1, 2006 and January 1, 2016), *infs*=0 (for subjects without prior infection history during January 1, 2006 and January 1, 2016), and age at the baseline date January 1, 2006 as the *Age* variable. A logistic regression set, having 64 models with linear terms and their pairwise interactions and corresponding to females/males aged 60-75, *APOE4* carriers, were analyzed and presented in ascending order by AIC value. Signf=1 means that all regression coefficient were significant (P-value<0.05) in a specific model, Signf=0 means the opposite. For regression model a short notation used. For instance, *risk* ~ 1 + *Age* + *dnmr* + *infs* + *dnmr***Age* + *infs***dnmr* denotes a standard logistic regression equation ln(*risk*/(1-*risk*)) = Intercept + b_1_**Age* + b_2_**dnmr* + b_3_**infs* + b_12_**Age***dnmr* + b_31_**infs***dnmr* where ln(x) natural logarithm, logarithm with base e = 2.718281828 , Intercept is a constant called the bias term (or intercept term), b_1_, b_2_, b_3_, b_12_, b_31_ are the regression coefficients corresponding to the *Age*, *dnmr*, *infs*, *Age***dnmr*, *infs***dnmr* terms in the regression model.

**Supplementary Table 13.** Logistic regression models, females/males aged 60-75 years, *APOE4* non-carriers.

| Regression Model | AIC | Signf |
| --- | --- | --- |
| **females/males,** *APOE4* **non-carriers, age 60-75** |  |  |
| *risk* ~ 1 + *Age* + *infs* + *infs*:*Age* + *infs*:*dnmr* | 4644.31 | 0 |
| *risk* ~ 1 + *Age* + *infs* + *infs*:*dnmr* | 4644.85 | 1 |
| *risk* ~ 1 + *Age* + *infs*:*Age* + *infs*:*dnmr* | 4645.56 | 1 |
| *risk* ~ 1 + *Age* + *infs* + *dnmr*:*Age* + *infs*:*Age* + *infs*:*dnmr* | 4645.88 | 0 |
| *risk* ~ 1 + *Age* + *dnmr* + *infs* + *infs*:*Age* + *infs*:*dnmr* | 4645.94 | 0 |
| *risk* ~ 1 + *Age* + *infs* + *dnmr*:*Age* + *infs*:*dnmr* | 4646.41 | 0 |
| *risk* ~ 1 + *Age* + *dnmr* + *infs* + *infs*:*dnmr* | 4646.48 | 0 |
| *risk* ~ 1 + *Age* + *dnmr* + *infs* + *dnmr*:*Age* + *infs*:*Age* + *infs*:*dnmr* | 4646.76 | 0 |
| *risk* ~ 1 + *Age* + *dnmr* + *infs* + *dnmr*:*Age* + *infs*:*dnmr* | 4646.83 | 0 |
| *risk* ~ 1 + *Age* + *dnmr*:*Age* + *infs*:*Age* + *infs*:*dnmr* | 4647.11 | 0 |
| *risk* ~ 1 + *Age* + *dnmr* + *infs*:*Age* + *infs*:*dnmr* | 4647.18 | 0 |
| *risk* ~ 1 + *Age* + *dnmr* + *dnmr*:*Age* + *infs*:*Age* + *infs*:*dnmr* | 4647.46 | 0 |
| *risk* ~ 1 + *Age* + *infs* + *infs*:*Age* | 4649.08 | 0 |
| *risk* ~ 1 + *Age* + *infs* | 4649.58 | 1 |
| *risk* ~ 1 + *Age* + *dnmr* + *infs* + *infs*:*Age* | 4649.95 | 0 |
| *risk* ~ 1 + *Age* + *infs* + *dnmr*:*Age* + *infs*:*Age* | 4650.04 | 0 |
| *risk* ~ 1 + *Age* + *infs*:*Age* | 4650.37 | 1 |
| *risk* ~ 1 + *Age* + *dnmr* + *infs* + *dnmr*:*Age* + *infs*:*Age* | 4650.42 | 0 |
| *risk* ~ 1 + *Age* + *dnmr* + *infs* | 4650.46 | 0 |
| *risk* ~ 1 + *Age* + *infs* + *dnmr*:*Age* | 4650.55 | 0 |
| *risk* ~ 1 + *Age* + *dnmr* + *infs* + *dnmr*:*Age* | 4650.91 | 0 |
| *risk* ~ 1 + *Age* + *dnmr* + *infs*:*Age* | 4651.25 | 0 |
| *risk* ~ 1 + *Age* + *dnmr*:*Age* + *infs*:*Age* | 4651.34 | 0 |
| *risk* ~ 1 + *Age* + *dnmr* + *dnmr*:*Age* + *infs*:*Age* | 4651.69 | 0 |
| *risk* ~ 1 + *Age* + *infs*:*dnmr* | 4677.56 | 1 |
| *risk* ~ 1 + *Age* + *dnmr*:*Age* + *infs*:*dnmr* | 4677.89 | 0 |
| *risk* ~ 1 + *Age* + *dnmr* + *infs*:*dnmr* | 4678.02 | 0 |
| *risk* ~ 1 + *Age* + *dnmr* + *dnmr*:*Age* + *infs*:*dnmr* | 4678.33 | 0 |
| *risk* ~ 1 + *infs* + *infs*:*Age* + *infs*:*dnmr* | 4686.52 | 1 |
| *risk* ~ 1 + *dnmr* + *infs* + *infs*:*Age* + *infs*:*dnmr* | 4688.14 | 0 |
| *risk* ~ 1 + *infs* + *dnmr*:*Age* + *infs*:*Age* + *infs*:*dnmr* | 4688.14 | 0 |
| *risk* ~ 1 + *infs*:*Age* + *infs*:*dnmr* | 4689.99 | 1 |
| *risk* ~ 1 + *dnmr* + *infs* + *dnmr*:*Age* + *infs*:*Age* + *infs*:*dnmr* | 4690.14 | 0 |
| *risk* ~ 1 + *infs* + *infs*:*Age* | 4691.29 | 1 |
| *risk* ~ 1 + *infs* + *infs*:*dnmr* | 4691.29 | 1 |
| *risk* ~ 1 + *dnmr* + *infs*:*Age* + *infs*:*dnmr* | 4691.63 | 0 |
| *risk* ~ 1 + *dnmr*:*Age* + *infs*:*Age* + *infs*:*dnmr* | 4691.67 | 0 |
| *risk* ~ 1 + *infs* + *dnmr*:*Age* + *infs*:*Age* | 4692.15 | 0 |
| *risk* ~ 1 + *dnmr* + *infs* + *infs*:*Age* | 4692.17 | 0 |
| *risk* ~ 1 + *dnmr* + *infs* + *infs*:*dnmr* | 4692.91 | 0 |
| *risk* ~ 1 + *infs* + *dnmr*:*Age* + *infs*:*dnmr* | 4692.95 | 0 |
| *risk* ~ 1 + *dnmr* + *dnmr*:*Age* + *infs*:*Age* + *infs*:*dnmr* | 4693.24 | 0 |
| *risk* ~ 1 + *Age* | 4693.83 | 1 |
| *risk* ~ 1 + *dnmr* + *infs* + *dnmr*:*Age* + *infs*:*Age* | 4694.08 | 0 |
| *risk* ~ 1 + *dnmr* + *infs* + *dnmr*:*Age* + *infs*:*dnmr* | 4694.44 | 0 |
| *risk* ~ 1 + *Age* + *dnmr* | 4694.70 | 0 |
| *risk* ~ 1 + *infs*:*Age* | 4694.71 | 1 |
| *risk* ~ 1 + *Age* + *dnmr*:*Age* | 4694.79 | 0 |
| *risk* ~ 1 + *Age* + *dnmr* + *dnmr*:*Age* | 4695.18 | 0 |
| *risk* ~ 1 + *dnmr*:*Age* + *infs*:*Age* | 4695.52 | 0 |
| *risk* ~ 1 + *dnmr* + *infs*:*Age* | 4695.57 | 0 |
| *risk* ~ 1 + *infs* | 4696.13 | 1 |
| *risk* ~ 1 + *infs* + *dnmr*:*Age* | 4696.94 | 0 |
| *risk* ~ 1 + *dnmr* + *infs* | 4696.99 | 0 |
| *risk* ~ 1 + *dnmr* + *dnmr*:*Age* + *infs*:*Age* | 4697.13 | 0 |
| *risk* ~ 1 + *dnmr* + *infs* + *dnmr*:*Age* | 4698.49 | 0 |
| *risk* ~ 1 + *infs*:*dnmr* | 4724.19 | 1 |
| *risk* ~ 1 + *dnmr* + *infs*:*dnmr* | 4724.64 | 0 |
| *risk* ~ 1 + *dnmr*:*Age* + *infs*:*dnmr* | 4724.71 | 0 |
| *risk* ~ 1 + *dnmr* + *dnmr*:*Age* + *infs*:*dnmr* | 4726.16 | 0 |
| *risk* ~ 1 | 4740.66 | 1 |
| *risk* ~ 1 + *dnmr*:*Age* | 4741.47 | 0 |
| *risk* ~ 1 + *dnmr* | 4741.52 | 0 |
| *risk* ~ 1 + *dnmr* + *dnmr*:*Age* | 4743.01 | 0 |

Note: Response variable *risk* is risk of AD+. Independent variables: *dnmr*=1 (DNMR<50), *dnmr*=0 (DNMR>=50), *infs*=1 (for subjects with prior infection history during January 1, 2006 and January 1, 2016), *infs*=0 (for subjects without prior infection history during January 1, 2006 and January 1, 2016), and age at the baseline date January 1, 2006 as the *Age* variable. A logistic regression set, having 64 models with linear terms and their pairwise interactions and corresponding to females/males aged 60-75, *APOE4* non-carriers, were analyzed and presented in ascending order by AIC value. Signf=1 means that all regression coefficient were significant (P-value<0.05) in a specific model, Signf=0 means the opposite. For regression model a short notation used. For instance, *risk* ~ 1 + *Age* + *dnmr* + *infs* + *dnmr***Age* + *infs***dnmr* denotes a standard logistic regression equation ln(*risk*/(1-*risk*)) = Intercept + b_1_**Age* + b_2_**dnmr* + b_3_**infs* + b_12_**Age***dnmr* + b_31_**infs***dnmr* where ln(x) natural logarithm, logarithm with base e = 2.718281828, Intercept is a constant called the bias term (or intercept term), b_1_, b_2_, b_3_, b_12_, b_31_ are the regression coefficients corresponding to the *Age*, *dnmr*, *infs*, *Age***dnmr*, *infs***dnmr* terms in the regression model.

Supplementary Table 14. Regression coefficients for the best model among all models considered in this study (*APOE4* carriers)

| Model/Term | Estimate | Std.Error | P-Value |
| --- | --- | --- | --- |
| **Best model, females/males,** *APOE4* **carriers, 60-75** |  |  |  |
| (Intercept) | -16.230 | 1.525 | <1.00e-20 |
| *Age* | 0.194 (1/year) | 0.024 | 2.69e-16 |

**Note:** here, we presented the result of regression analysis of the set with all possible logistic regression models (64 models) for females/males, *APOE4* non-carriers, aged 60-75 years having linear variables *Age, dnmr, infs* and their pairwise interactions, having risk of AD+ as a response variable *risk* and independent variables: *dnmr*=1 (DNMR<=50), *dnmr*=0 (DNMR>50), *infs*=1 (for subjects with history of infections during January 1, 2006 and January 1, 2016), *infs*=0 (for subjects without history of infections during January 1, 2006 and January 1, 2016), and age at the baseline date January 1, 2006 as the *Age* variable. The logistic regression models were evaluated using the Akaike information criterion (AIC). Results for all models that are presented in ascending order by AIC value in Supplementary Table 12. The optimal (best), with respect to the minimal AIC criteria, significant result for regression model was found for the regression set described above and regression coefficients of the best model are presented in this table. Scientific notation ‘e’ means that the base number is multiplied by 10 raised to the given power.

Supplementary Table 15. Regression coefficients for the best model among all models considered in this study (*APOE4* non-carriers)

| Model/Term | Estimate | Std.Error | P-Value |
| --- | --- | --- | --- |
| **Best model, females/males,** *APOE4* **non-carriers, 60-75** |  |  |  |
| (Intercept) | -16.112 | 1.592 | <1.00e-20 |
| *Age* | 0.172 (1/year) | 0.025 | 3.67e-12 |
| infs | 0.725 | 0.116 | 3.89e-10 |
| *infs***dnmr* | 0.800 | 0.280 | 4.35e-03 |

**Note:** here, we presented the result of regression analysis of the set with all possible logistic regression models (64 models) for females/males, *APOE4* non-carriers, aged 60-75 years having linear variables *Age, dnmr, infs* and their pairwise interactions, having risk of AD+ as a response variable *risk* and independent variables: *dnmr*=1 (DNMR<=50), *dnmr*=0 (DNMR>50), *infs*=1 (for subjects with history of infections during January 1, 2006 and January 1, 2016), *infs*=0 (for subjects without history of infections during January 1, 2006 and January 1, 2016), and age at the baseline date January 1, 2006 as the *Age* variable. The logistic regression models were evaluated using the Akaike information criterion (AIC). Results for all models that are presented in ascending order by AIC value in Supplementary Table 13. The optimal (best), with respect to the minimal AIC criteria, significant result for regression model was found for the regression set described above and regression coefficients of the best model are presented in this table. Scientific notation ‘e’ means that the base number is multiplied by 10 raised to the given power.

Supplementary Table 16. Regression coefficients for model with main factors (reference regression model)

| Model/Term | Estimate | Std.Error | P-Value |
| --- | --- | --- | --- |
| **females/males, 60-75** |  |  |  |
| (Intercept) | -15.500 | 0.983 | <1.00e-20 |
| *Age* | 0.171 (1/year) | 0.015 | <1.00e-20 |
| *dnmr* | 0.157 | 0.120 | 1.92e-01 |
| infs | 0.474 | 0.073 | 1.06e-10 |

**Note:** here we presented the result of regression analysis of the logistic regression model with only main effects of age, DNMR, and infections for females/males aged 60-75 years, having linear variables *Age, dnmr , infs* and without pairwise interactions **(**reference regression model)**,** having risk of AD+ as a response variable *risk* and independent variables: *dnmr*=1 (DNMR<=50), *dnmr*=0 (DNMR>50), *infs*=1 (for subjects with history of infections during January 1, 2006 and January 1, 2016), *infs*=0 (for subjects without history of infections during January 1, 2006 and January 1, 2016), and age at the baseline date January 1, 2006 as the *Age* variable. The regression coefficients of the model are presented in this table. Scientific notation ‘e’ means that the base number is multiplied by 10 raised to the given power.

References

International Statistical Classification of Diseases and Related Health Problems 10th Revision (2019). Available online at: https://icd.who.int/browse10/2019/en (accessed February 28, 2024).
