# Supplementary material for: Traffic-related air pollution significantly aggravates the detrimental effect of infections on the risk of Alzheimer’s disease and other dementias, especially in non-carriers of APOE4
Source: Front Dement. 2026 Jan 12;4:1668381. doi: 10.3389/frdem.2025.1668381 (PMC12833968; doi:10.3389/frdem.2025.1668381)
Supplement: Supplementary file 2 [file Supplementary_file_2.docx]

Supplementary Material 2

# Supplementary Tables

**Supplementary Table 2.1** Regression coefficients (all having P-value<0.05) for the logistic regression models, females/males *age*d 60-75 years.

| Model | Num | Model/Term | Estimate | Std.err | P-Value |
| --- | --- | --- | --- | --- | --- |
|  |  |  |  |  |  |
| *risk* ~ 1 + *Age* + *infs* + *infs***dnmr* | 2 | (Intercept) | -15.489 | 0.983 | 6.16e-56 |
|  |  | *Age* | 0.171 | 0.015 | 6.47e-29 |
|  |  | *infs* | 0.429 | 0.077 | 2.28e-08 |
|  |  | *infs***dnmr* | 0.558 | 0.210 | 7.94e-03 |
| *risk* ~ 1 + *Age* + *infs***Age* + *infs***dnmr* | 3 | (Intercept) | -15.381 | 0.983 | 3.85e-55 |
|  |  | *Age* | 0.169 | 0.015 | 2.43e-28 |
|  |  | *Age***infs* | 0.007 | 0.001 | 3.37e-08 |
|  |  | *infs***dnmr* | 0.563 | 0.210 | 7.51e-03 |
| *risk* ~ 1 + *Age* + *infs* | 15 | (Intercept) | -15.483 | 0.983 | 6.46e-56 |
|  |  | *Age* | 0.171 | 0.015 | 6.75e-29 |
|  |  | *infs* | 0.475 | 0.073 | 1.04e-10 |
| *risk* ~ 1 + *Age* + *infs***Age* | 19 | (Intercept) | -15.363 | 0.983 | 4.88e-55 |
|  |  | *Age* | 0.169 | 0.015 | 2.91e-28 |
|  |  | *Age***infs* | 0.007 | 0.001 | 1.61e-10 |
| *risk* ~ 1 + *Age* + *infs***dnmr* | 25 | (Intercept) | -15.489 | 0.986 | 1.48e-55 |
|  |  | *Age* | 0.172 | 0.015 | 4.04e-29 |
|  |  | *infs***dnmr* | 0.908 | 0.202 | 6.66e-06 |
| *risk* ~ 1 + *Age* | 29 | (Intercept) | -15.481 | 0.987 | 1.87e-55 |
|  |  | *Age* | 0.172 | 0.015 | 3.93e-29 |
| *risk* ~ 1 + *infs* + *infs***Age* + *infs***dnmr* | 33 | (Intercept) | -4.584 | 0.036 | 0.00e+00 |
|  |  | *infs* | -7.704 | 1.927 | 6.38e-05 |
|  |  | *infs***Age* | 0.127 | 0.030 | 2.18e-05 |
|  |  | *infs***dnmr* | 0.557 | 0.210 | 8.10e-03 |
| *risk* ~ 1 + *dnmr* + *infs* + *dnmr***Age* + *infs***Age* | 37 | (Intercept) | -4.593 | 0.037 | 0.00e+00 |
|  |  | *dnmr* | -7.541 | 3.671 | 4.00e-02 |
|  |  | *infs* | -7.079 | 1.944 | 2.71e-04 |
|  |  | *dnmr***Age* | 0.120 | 0.057 | 3.54e-02 |
|  |  | *infs***Age* | 0.118 | 0.030 | 9.38e-05 |
| *risk* ~ 1 + *infs* + *infs***Age* | 38 | (Intercept) | -4.584 | 0.036 | 0.00e+00 |
|  |  | *infs* | -7.639 | 1.925 | 7.24e-05 |
|  |  | *infs***Age* | 0.127 | 0.030 | 2.24e-05 |
| *risk* ~ 1 + *dnmr* + *dnmr***Age* + *infs***Age* + *infs***dnmr* | 41 | (Intercept) | -4.586 | 0.037 | 0.00e+00 |
|  |  | *dnmr* | -9.430 | 3.623 | 9.25e-03 |
|  |  | *dnmr***Age* | 0.148 | 0.056 | 8.90e-03 |
|  |  | *Age***infs* | 0.007 | 0.001 | 7.06e-09 |
|  |  | *dnmr***infs* | 0.540 | 0.257 | 3.55e-02 |
| *risk* ~ 1 + *dnmr* + *infs* + *dnmr***Age* + *infs***dnmr* | 42 | (Intercept) | -4.584 | 0.037 | 0.00e+00 |
|  |  | *dnmr* | -9.595 | 3.623 | 8.09e-03 |
|  |  | *infs* | 0.434 | 0.077 | 1.85e-08 |
|  |  | *dnmr***Age* | 0.150 | 0.056 | 7.79e-03 |
|  |  | *dnmr***infs* | 0.555 | 0.257 | 3.06e-02 |
| *risk* ~ 1 + *dnmr* + *dnmr***Age* + *infs***Age* | 43 | (Intercept) | -4.597 | 0.037 | 0.00e+00 |
|  |  | *dnmr* | -9.405 | 3.636 | 9.70e-03 |
|  |  | *dnmr***Age* | 0.150 | 0.057 | 8.24e-03 |
|  |  | *Age***infs* | 0.008 | 0.001 | 2.12e-11 |
| *risk* ~ 1 + *infs***Age* + *infs***dnmr* | 44 | (Intercept) | -4.587 | 0.036 | 0.00e+00 |
|  |  | *infs***Age* | 0.007 | 0.001 | 4.70e-09 |
|  |  | *infs***dnmr* | 0.544 | 0.210 | 9.56e-03 |
| *risk* ~ 1 + *dnmr* + *infs* + *dnmr***Age* | 45 | (Intercept) | -4.594 | 0.037 | 0.00e+00 |
|  |  | *dnmr* | -9.534 | 3.636 | 8.73e-03 |
|  |  | *infs* | 0.480 | 0.073 | 6.37e-11 |
|  |  | *dnmr***Age* | 0.152 | 0.057 | 7.40e-03 |
| *risk* ~ 1 + *infs* + *infs***dnmr* | 46 | (Intercept) | -4.584 | 0.036 | 0.00e+00 |
|  |  | *infs* | 0.434 | 0.077 | 1.42e-08 |
|  |  | *infs***dnmr* | 0.553 | 0.210 | 8.41e-03 |
| *risk* ~ 1 + *infs***Age* | 49 | (Intercept) | -4.587 | 0.036 | 0.00e+00 |
|  |  | *infs***Age* | 0.008 | 0.001 | 1.80e-11 |
| *risk* ~ 1 + *infs* | 54 | (Intercept) | -4.584 | 0.036 | 0.00e+00 |
|  |  | *infs* | 0.480 | 0.073 | 6.04e-11 |
| *risk* ~ 1 + *dnmr* + *dnmr***Age* + *infs***dnmr* | 57 | (Intercept) | -4.500 | 0.032 | 0.00e+00 |
|  |  | *dnmr* | -9.679 | 3.623 | 7.55e-03 |
|  |  | *dnmr***Age* | 0.150 | 0.056 | 7.79e-03 |
|  |  | *dnmr***infs* | 0.989 | 0.245 | 5.31e-05 |
| *risk* ~ 1 + *infs***dnmr* | 58 | (Intercept) | -4.504 | 0.032 | 0.00e+00 |
|  |  | *infs***dnmr* | 0.908 | 0.201 | 6.43e-06 |
| *risk* ~ 1 + *dnmr* + *dnmr***Age* | 61 | (Intercept) | -4.500 | 0.032 | 0.00e+00 |
|  |  | *dnmr* | -9.619 | 3.649 | 8.38e-03 |
|  |  | *dnmr***Age* | 0.153 | 0.057 | 7.09e-03 |
| 1 | 62 | (Intercept) | -4.489 | 0.031 | 0.00e+00 |

**Note:** here, we presented the regressions coefficients obtained from the regression analysis for the subset (21 models with Signf=1; Supplementary Table 11) of all possible logistic regression (64 models; Supplementary Table 11) for females/males, aged 60-75 years. Response variable *risk* is risk of AD+. Independent variables* *dnmr*=1 (*dnmr*<50), *dnmr*=0 (*dnmr*>=50), *infs*=1 (for subjects with prior infection history during January 1, 2006 and January 1, 2016), *infs*=0 (for subjects without prior infection history during January 1, 2006 and January 1, 2016), and *age* at the baseline date January 1, 2006 as the *Age* variable. In Supplementary Table 11, a logistic regression set, having 64 models with linear terms and their pairwise interactions and corresponding to females/ males *age*d 60-75 were analyzed and presented in ascending order by AIC value. Signf=1 means that all regression coefficient were significant (P-value<0.05) in a specific model, Signf=0 means the opposite. Here and in the Supplementary Table 11, for regression model a short notation for logistic regression used. For instance, *risk* ~ 1 + *Age* + *dnmr* + *infs* + *dnmr***Age* + *infs***dnmr* denotes a standard logistic regression equation ln(*risk*/(1-*risk*)) = Intercept + b_1_**Age* + b_2_**dnmr* + b_3_**infs* + b_12_**Age***dnmr* + b_31_**infs***dnmr* where ln(x) natural logarithm, Intercept is a constant called the bias term (or intercept term), b_1_, b_2_, b_3_, b_12_, b_31_ are the regression coefficients corresponding to the *Age*, *dnmr*, *infs*, *Age***dnmr*, *infs***dnmr* terms in the regression model. The 'Model’ column shows the models presented in the short notation. The columns ‘Num’ gives the number of the model in the list arranged from smallest to largest AIC value (Supplementary Table 11). The numbers in the ‘Estimate‘ column correspond to the coefficients for the regression terms shown in the 'Model/Term’ column. The ‘Std.err’ and ‘P-Value’ presents respectively the standard error and the P-value. For all terms containing variable *Age* a unit of their measurement is 1/year; all other terms are the dimensionless quantities. Scientific notation ‘e’ means that the base number is multiplied by 10 raised to the given power.

**Supplementary Table 2.2** Regression coefficients (not all having P-value<0.05) for the logistic regression models, females/males *age*d 60-75 years.

| Model | Num | Model/Term | Estimate | Std.err | P-Value |
| --- | --- | --- | --- | --- | --- |
|  |  |  |  |  |  |
| *risk* ~ 1 + *Age* + *infs* + *infs***Age* + *infs***dnmr* | 1 | (Intercept) | -16.453 | 1.142 | 4.53e-47 |
|  |  | *Age* | 0.186 | 0.018 | 1.33e-25 |
|  |  | *infs* | 4.166 | 2.239 | 6.28e-02 |
|  |  | *Age***infs* | -0.058 | 0.035 | 9.53e-02 |
|  |  | *infs***dnmr* | 0.557 | 0.210 | 8.10e-03 |
| *risk* ~ 1 + *Age* + *infs* + *dnmr***Age* + *infs***Age* + *infs***dnmr* | 4 | (Intercept) | -16.453 | 1.142 | 4.54e-47 |
|  |  | *Age* | 0.186 | 0.018 | 1.33e-25 |
|  |  | *infs* | 4.166 | 2.239 | 6.29e-02 |
|  |  | *Age***dnmr* | 0.000 | 0.002 | 9.91e-01 |
|  |  | *Age***infs* | -0.058 | 0.035 | 9.53e-02 |
|  |  | *infs***dnmr* | 0.558 | 0.256 | 2.93e-02 |
| *risk* ~ 1 + *Age* + *dnmr* + *infs* + *infs***Age* + *infs***dnmr* | 5 | (Intercept) | -16.453 | 1.142 | 4.59e-47 |
|  |  | *Age* | 0.186 | 0.018 | 1.33e-25 |
|  |  | *dnmr* | 0.000 | 0.147 | 9.99e-01 |
|  |  | *infs* | 4.166 | 2.239 | 6.28e-02 |
|  |  | *Age***infs* | -0.058 | 0.035 | 9.53e-02 |
|  |  | *dnmr***infs* | 0.557 | 0.256 | 2.99e-02 |
| *risk* ~ 1 + *Age* + *infs* + *dnmr***Age* + *infs***dnmr* | 6 | (Intercept) | -15.489 | 0.983 | 6.16e-56 |
|  |  | *Age* | 0.171 | 0.015 | 6.48e-29 |
|  |  | *infs* | 0.428 | 0.077 | 2.91e-08 |
|  |  | *Age***dnmr* | 0.000 | 0.002 | 9.85e-01 |
|  |  | *infs***dnmr* | 0.561 | 0.257 | 2.88e-02 |
| *risk* ~ 1 + *Age* + *dnmr* + *infs* + *infs***dnmr* | 7 | (Intercept) | -15.489 | 0.983 | 6.29e-56 |
|  |  | *Age* | 0.171 | 0.015 | 6.47e-29 |
|  |  | *dnmr* | -0.001 | 0.147 | 9.96e-01 |
|  |  | *infs* | 0.429 | 0.077 | 2.88e-08 |
|  |  | *dnmr***infs* | 0.559 | 0.257 | 2.93e-02 |
| *risk* ~ 1 + *Age* + *dnmr***Age* + *infs***Age* + *infs***dnmr* | 8 | (Intercept) | -15.381 | 0.983 | 3.85e-55 |
|  |  | *Age* | 0.169 | 0.015 | 2.44e-28 |
|  |  | *Age***dnmr* | 0.000 | 0.002 | 9.78e-01 |
|  |  | *Age***infs* | 0.007 | 0.001 | 4.28e-08 |
|  |  | *dnmr***infs* | 0.567 | 0.257 | 2.73e-02 |
| *risk* ~ 1 + *Age* + *dnmr* + *infs***Age* + *infs***dnmr* | 9 | (Intercept) | -15.381 | 0.983 | 3.91e-55 |
|  |  | *Age* | 0.169 | 0.015 | 2.43e-28 |
|  |  | *dnmr* | -0.002 | 0.147 | 9.90e-01 |
|  |  | *Age***infs* | 0.007 | 0.001 | 4.24e-08 |
|  |  | *dnmr***infs* | 0.564 | 0.257 | 2.79e-02 |
| *risk* ~ 1 + *Age* + *dnmr* + *infs* + *dnmr***Age* + *infs***Age* + *infs***dnmr* | 10 | (Intercept) | -16.517 | 1.167 | 1.71e-45 |
|  |  | *Age* | 0.187 | 0.018 | 7.69e-25 |
|  |  | *dnmr* | 1.005 | 3.772 | 7.90e-01 |
|  |  | *infs* | 4.126 | 2.244 | 6.60e-02 |
|  |  | *Age***dnmr* | -0.016 | 0.059 | 7.90e-01 |
|  |  | *Age***infs* | -0.058 | 0.035 | 9.96e-02 |
|  |  | *dnmr***infs* | 0.554 | 0.257 | 3.09e-02 |
| *risk* ~ 1 + *Age* + *infs* + *infs***Age* | 11 | (Intercept) | -16.453 | 1.142 | 4.53e-47 |
|  |  | *Age* | 0.186 | 0.018 | 1.33e-25 |
|  |  | *infs* | 4.230 | 2.238 | 5.88e-02 |
|  |  | *Age***infs* | -0.058 | 0.035 | 9.35e-02 |
| *risk* ~ 1 + *Age* + *dnmr* + *infs* + *infs***Age* | 12 | (Intercept) | -16.469 | 1.142 | 3.76e-47 |
|  |  | *Age* | 0.186 | 0.018 | 1.27e-25 |
|  |  | *dnmr* | 0.157 | 0.120 | 1.91e-01 |
|  |  | *infs* | 4.231 | 2.238 | 5.87e-02 |
|  |  | *Age***infs* | -0.058 | 0.035 | 9.34e-02 |
| *risk* ~ 1 + *Age* + *infs* + *dnmr***Age* + *infs***Age* | 13 | (Intercept) | -16.457 | 1.142 | 4.30e-47 |
|  |  | *Age* | 0.186 | 0.018 | 1.41e-25 |
|  |  | *infs* | 4.231 | 2.238 | 5.87e-02 |
|  |  | *Age***dnmr* | 0.002 | 0.002 | 1.95e-01 |
|  |  | *Age***infs* | -0.058 | 0.035 | 9.34e-02 |
| *risk* ~ 1 + *Age* + *dnmr* + *infs* + *dnmr***Age* + *infs***dnmr* | 14 | (Intercept) | -15.593 | 1.021 | 1.27e-52 |
|  |  | *Age* | 0.172 | 0.016 | 2.15e-27 |
|  |  | *dnmr* | 1.415 | 3.764 | 7.07e-01 |
|  |  | *infs* | 0.428 | 0.077 | 2.91e-08 |
|  |  | *Age***dnmr* | -0.022 | 0.059 | 7.07e-01 |
|  |  | *dnmr***infs* | 0.560 | 0.257 | 2.89e-02 |
| *risk* ~ 1 + *Age* + *dnmr* + *infs* | 16 | (Intercept) | -15.499 | 0.983 | 5.18e-56 |
|  |  | *Age* | 0.171 | 0.015 | 6.42e-29 |
|  |  | *dnmr* | 0.157 | 0.120 | 1.91e-01 |
|  |  | *infs* | 0.475 | 0.073 | 1.06e-10 |
| *risk* ~ 1 + *Age* + *dnmr* + *dnmr***Age* + *infs***Age* + *infs***dnmr* | 17 | (Intercept) | -15.488 | 1.022 | 6.63e-52 |
|  |  | *Age* | 0.171 | 0.016 | 7.12e-27 |
|  |  | *dnmr* | 1.462 | 3.764 | 6.98e-01 |
|  |  | *Age***dnmr* | -0.023 | 0.059 | 6.97e-01 |
|  |  | *Age***infs* | 0.007 | 0.001 | 4.26e-08 |
|  |  | *dnmr***infs* | 0.566 | 0.257 | 2.73e-02 |
| *risk* ~ 1 + *Age* + *infs* + *dnmr***Age* | 18 | (Intercept) | -15.487 | 0.983 | 6.11e-56 |
|  |  | *Age* | 0.170 | 0.015 | 7.32e-29 |
|  |  | *infs* | 0.475 | 0.073 | 1.06e-10 |
|  |  | *Age***dnmr* | 0.002 | 0.002 | 1.96e-01 |
| *risk* ~ 1 + *Age* + *dnmr* + *infs***Age* | 20 | (Intercept) | -15.379 | 0.983 | 3.92e-55 |
|  |  | *Age* | 0.169 | 0.015 | 2.76e-28 |
|  |  | *dnmr* | 0.157 | 0.120 | 1.91e-01 |
|  |  | *Age***infs* | 0.007 | 0.001 | 1.63e-10 |
| *risk* ~ 1 + *Age* + *dnmr***Age* + *infs***Age* | 21 | (Intercept) | -15.367 | 0.983 | 4.61e-55 |
|  |  | *Age* | 0.169 | 0.015 | 3.15e-28 |
|  |  | *Age***dnmr* | 0.002 | 0.002 | 1.96e-01 |
|  |  | *Age***infs* | 0.007 | 0.001 | 1.63e-10 |
| *risk* ~ 1 + *Age* + *dnmr* + *infs* + *dnmr***Age* + *infs***Age* | 22 | (Intercept) | -16.565 | 1.175 | 3.73e-45 |
|  |  | *Age* | 0.187 | 0.018 | 1.15e-24 |
|  |  | *dnmr* | 1.468 | 3.770 | 6.97e-01 |
|  |  | *infs* | 4.231 | 2.238 | 5.87e-02 |
|  |  | *Age***dnmr* | -0.020 | 0.059 | 7.28e-01 |
|  |  | *Age***infs* | -0.058 | 0.035 | 9.34e-02 |
| *risk* ~ 1 + *Age* + *dnmr* + *infs* + *dnmr***Age* | 23 | (Intercept) | -15.594 | 1.021 | 1.13e-52 |
|  |  | *Age* | 0.172 | 0.016 | 2.26e-27 |
|  |  | *dnmr* | 1.466 | 3.776 | 6.98e-01 |
|  |  | *infs* | 0.475 | 0.073 | 1.06e-10 |
|  |  | *Age***dnmr* | -0.020 | 0.059 | 7.29e-01 |
| *risk* ~ 1 + *Age* + *dnmr* + *dnmr***Age* + *infs***Age* | 24 | (Intercept) | -15.474 | 1.021 | 7.43e-52 |
|  |  | *Age* | 0.170 | 0.016 | 8.81e-27 |
|  |  | *dnmr* | 1.465 | 3.777 | 6.98e-01 |
|  |  | *Age***dnmr* | -0.020 | 0.059 | 7.29e-01 |
|  |  | *Age***infs* | 0.007 | 0.001 | 1.63e-10 |
| *risk* ~ 1 + *Age* + *dnmr***Age* + *infs***dnmr* | 26 | (Intercept) | -15.488 | 0.986 | 1.49e-55 |
|  |  | *Age* | 0.172 | 0.015 | 3.87e-29 |
|  |  | *Age***dnmr* | -0.001 | 0.002 | 5.56e-01 |
|  |  | *dnmr***infs* | 0.990 | 0.245 | 5.28e-05 |
| *risk* ~ 1 + *Age* + *dnmr* + *infs***dnmr* | 27 | (Intercept) | -15.482 | 0.986 | 1.67e-55 |
|  |  | *Age* | 0.172 | 0.015 | 4.15e-29 |
|  |  | *dnmr* | -0.084 | 0.146 | 5.66e-01 |
|  |  | *dnmr***infs* | 0.988 | 0.245 | 5.44e-05 |
| *risk* ~ 1 + *Age* + *dnmr* + *dnmr***Age* + *infs***dnmr* | 28 | (Intercept) | -15.592 | 1.025 | 3.06e-52 |
|  |  | *Age* | 0.174 | 0.016 | 1.37e-27 |
|  |  | *dnmr* | 1.414 | 3.765 | 7.07e-01 |
|  |  | *Age***dnmr* | -0.023 | 0.059 | 6.91e-01 |
|  |  | *dnmr***infs* | 0.989 | 0.245 | 5.31e-05 |
| *risk* ~ 1 + *Age* + *dnmr* | 30 | (Intercept) | -15.496 | 0.987 | 1.49e-55 |
|  |  | *Age* | 0.172 | 0.015 | 3.73e-29 |
|  |  | *dnmr* | 0.158 | 0.120 | 1.88e-01 |
| *risk* ~ 1 + *Age* + *dnmr***Age* | 31 | (Intercept) | -15.485 | 0.987 | 1.76e-55 |
|  |  | *Age* | 0.172 | 0.015 | 4.26e-29 |
|  |  | *Age***dnmr* | 0.002 | 0.002 | 1.92e-01 |
| *risk* ~ 1 + *Age* + *dnmr* + *dnmr***Age* | 32 | (Intercept) | -15.592 | 1.025 | 3.06e-52 |
|  |  | *Age* | 0.174 | 0.016 | 1.37e-27 |
|  |  | *dnmr* | 1.473 | 3.790 | 6.97e-01 |
|  |  | *Age***dnmr* | -0.020 | 0.059 | 7.29e-01 |
| *risk* ~ 1 + *dnmr* + *infs* + *dnmr***Age* + *infs***Age* + *infs***dnmr* | 34 | (Intercept) | -4.584 | 0.037 | 0.00e+00 |
|  |  | *dnmr* | -6.929 | 3.706 | 6.16e-02 |
|  |  | *infs* | -7.002 | 1.962 | 3.59e-04 |
|  |  | *dnmr***Age* | 0.109 | 0.058 | 6.07e-02 |
|  |  | *infs***Age* | 0.116 | 0.031 | 1.40e-04 |
|  |  | *dnmr***infs* | 0.506 | 0.259 | 5.03e-02 |
| *risk* ~ 1 + *dnmr* + *infs* + *infs***Age* + *infs***dnmr* | 35 | (Intercept) | -4.584 | 0.037 | 0.00e+00 |
|  |  | *dnmr* | -0.006 | 0.147 | 9.68e-01 |
|  |  | *infs* | -7.704 | 1.927 | 6.37e-05 |
|  |  | *infs***Age* | 0.127 | 0.030 | 2.18e-05 |
|  |  | *dnmr***infs* | 0.563 | 0.256 | 2.82e-02 |
| *risk* ~ 1 + *infs* + *dnmr***Age* + *infs***Age* + *infs***dnmr* | 36 | (Intercept) | -4.584 | 0.037 | 0.00e+00 |
|  |  | *infs* | -7.703 | 1.927 | 6.39e-05 |
|  |  | *dnmr***Age* | 0.000 | 0.002 | 9.73e-01 |
|  |  | *infs***Age* | 0.127 | 0.030 | 2.18e-05 |
|  |  | *infs***dnmr* | 0.552 | 0.257 | 3.16e-02 |
| *risk* ~ 1 + *infs* + *dnmr***Age* + *infs***Age* | 39 | (Intercept) | -4.595 | 0.037 | 0.00e+00 |
|  |  | *infs* | -7.632 | 1.925 | 7.38e-05 |
|  |  | *dnmr***Age* | 0.003 | 0.002 | 1.79e-01 |
|  |  | *infs***Age* | 0.127 | 0.030 | 2.29e-05 |
| *risk* ~ 1 + *dnmr* + *infs* + *infs***Age* | 40 | (Intercept) | -4.594 | 0.037 | 0.00e+00 |
|  |  | *dnmr* | 0.152 | 0.120 | 2.04e-01 |
|  |  | *infs* | -7.643 | 1.925 | 7.20e-05 |
|  |  | *infs***Age* | 0.127 | 0.030 | 2.23e-05 |
| *risk* ~ 1 + *dnmr***Age* + *infs***Age* + *infs***dnmr* | 47 | (Intercept) | -4.588 | 0.037 | 0.00e+00 |
|  |  | *dnmr***Age* | 0.000 | 0.002 | 9.32e-01 |
|  |  | *Age***infs* | 0.007 | 0.001 | 5.72e-09 |
|  |  | *dnmr***infs* | 0.532 | 0.256 | 3.75e-02 |
| *risk* ~ 1 + *dnmr* + *infs***Age* + *infs***dnmr* | 48 | (Intercept) | -4.587 | 0.037 | 0.00e+00 |
|  |  | *dnmr* | -0.003 | 0.147 | 9.83e-01 |
|  |  | *infs***Age* | 0.007 | 0.001 | 6.16e-09 |
|  |  | *dnmr***infs* | 0.547 | 0.256 | 3.27e-02 |
| *risk* ~ 1 + *infs* + *dnmr***Age* + *infs***dnmr* | 50 | (Intercept) | -4.585 | 0.037 | 0.00e+00 |
|  |  | *infs* | 0.435 | 0.077 | 1.72e-08 |
|  |  | *dnmr***Age* | 0.000 | 0.002 | 9.46e-01 |
|  |  | *infs***dnmr* | 0.544 | 0.256 | 3.34e-02 |
| *risk* ~ 1 + *dnmr* + *infs* + *infs***dnmr* | 51 | (Intercept) | -4.584 | 0.037 | 0.00e+00 |
|  |  | *dnmr* | -0.006 | 0.147 | 9.68e-01 |
|  |  | *infs* | 0.434 | 0.077 | 1.85e-08 |
|  |  | *dnmr***infs* | 0.559 | 0.256 | 2.91e-02 |
| *risk* ~ 1 + *dnmr***Age* + *infs***Age* | 52 | (Intercept) | -4.598 | 0.037 | 0.00e+00 |
|  |  | *dnmr***Age* | 0.003 | 0.002 | 1.75e-01 |
|  |  | *Age***infs* | 0.008 | 0.001 | 1.83e-11 |
| *risk* ~ 1 + *dnmr* + *infs***Age* | 53 | (Intercept) | -4.597 | 0.037 | 0.00e+00 |
|  |  | *dnmr* | 0.152 | 0.120 | 2.06e-01 |
|  |  | *infs***Age* | 0.008 | 0.001 | 1.82e-11 |
| *risk* ~ 1 + *infs* + *dnmr***Age* | 55 | (Intercept) | -4.595 | 0.037 | 0.00e+00 |
|  |  | *infs* | 0.480 | 0.073 | 6.11e-11 |
|  |  | *dnmr***Age* | 0.003 | 0.002 | 1.74e-01 |
| *risk* ~ 1 + *dnmr* + *infs* | 56 | (Intercept) | -4.594 | 0.037 | 0.00e+00 |
|  |  | *dnmr* | 0.152 | 0.120 | 2.06e-01 |
|  |  | *infs* | 0.480 | 0.073 | 6.11e-11 |
| *risk* ~ 1 + *dnmr* + *infs***dnmr* | 59 | (Intercept) | -4.500 | 0.032 | 0.00e+00 |
|  |  | *dnmr* | -0.090 | 0.146 | 5.37e-01 |
|  |  | *dnmr***infs* | 0.993 | 0.244 | 4.80e-05 |
| *risk* ~ 1 + *dnmr***Age* + *infs***dnmr* | 60 | (Intercept) | -4.501 | 0.032 | 0.00e+00 |
|  |  | *dnmr***Age* | -0.001 | 0.002 | 6.08e-01 |
|  |  | *dnmr***infs* | 0.979 | 0.244 | 5.94e-05 |
| *risk* ~ 1 + *dnmr***Age* | 63 | (Intercept) | -4.501 | 0.032 | 0.00e+00 |
|  |  | *dnmr***Age* | 0.003 | 0.002 | 1.72e-01 |
| *risk* ~ 1 + *dnmr* | 64 | (Intercept) | -4.500 | 0.032 | 0.00e+00 |
|  |  | *dnmr* | 0.153 | 0.120 | 2.03e-01 |

**Note:** here, we presented the regressions coefficients obtained from the regression analysis for the subset (43 models with Signf=0; Supplementary Table 11) of all possible logistic regression (64 models; Supplementary Table 11) for females/males, aged 60-75 years. Response variable *risk* is risk of AD+. Independent variables* *dnmr*=1 (*dnmr*<50), *dnmr*=0 (*dnmr*>=50), *infs*=1 (for subjects with prior infection history during January 1, 2006 and January 1, 2016), *infs*=0 (for subjects without prior infection history during January 1, 2006 and January 1, 2016), and *age* at the baseline date January 1, 2006 as the *Age* variable. In Supplementary Table 11, a logistic regression set, having 64 models with linear terms and their pairwise interactions and corresponding to females/ males *age*d 60-75 were analyzed and presented in ascending order by AIC value. Signf=1 means that all regression coefficient were significant (P-value<0.05) in a specific model, Signf=0 means the opposite. Here and in the Supplementary Table 11, for regression model a short notation for logistic regression used. For instance, *risk* ~ 1 + *Age* + *dnmr* + *infs* + *dnmr***Age* + *infs***dnmr* denotes a standard logistic regression equation ln(*risk*/(1-*risk*)) = Intercept + b_1_**Age* + b_2_**dnmr* + b_3_**infs* + b_12_**Age***dnmr* + b_31_**infs***dnmr* where ln(x) natural logarithm, Intercept is a constant called the bias term (or intercept term), b_1_, b_2_, b_3_, b_12_, b_31_ are the regression coefficients corresponding to the *Age*, *dnmr*, *infs*, *Age***dnmr*, *infs***dnmr* terms in the regression model. The 'Model’ column shows the models presented in the short notation. The columns ‘Num’ gives the number of the model in the list arranged from smallest to largest AIC value (Supplementary Table 11). The numbers in the ‘Estimate‘ column correspond to the coefficients for the regression terms shown in the 'Model/Term’ column. The ‘Std.err’ and ‘P-Value’ presents respectively the standard error and the P-value. For all terms containing variable *Age* a unit of their measurement is 1/year; all other terms are the dimensionless quantities. Scientific notation ‘e’ means that the base number is multiplied by 10 raised to the given power.

**Supplementary Table 2.3** Regression coefficients (all having P-value<0.05) for the logistic regression models, females/males *age*d 60-75 years, *APOE4* carriers.

| Model | Num | Model/Term | Estimate | Std.err | P-Value |
| --- | --- | --- | --- | --- | --- |
|  |  |  |  |  |  |
| risk ~ 1 + *Age* | 1 | (Intercept) | -16.230 | 1.525 | 1.95e-26 |
|  |  | *Age* | 0.194 | 0.024 | 2.69e-16 |
| risk ~ 1 + *infs* + *infs*:*Age* | 33 | (Intercept) | -3.846 | 0.052 | 0.00e+00 |
|  |  | *infs* | -15.198 | 3.557 | 1.93e-05 |
|  |  | *infs*:*Age* | 0.240 | 0.055 | 1.34e-05 |
| risk ~ 1 + *dnmr* + *dnmr*:*Age* | 41 | (Intercept) | -3.831 | 0.049 | 0.00e+00 |
|  |  | *dnmr* | -12.375 | 5.577 | 2.65e-02 |
|  |  | *dnmr*:*Age* | 0.196 | 0.087 | 2.40e-02 |
| risk ~ 1 | 45 | (Intercept) | -3.821 | 0.048 | 0.00e+00 |

**Note:** here, we presented the regressions coefficients obtained from the regression analysis for the subset (21 models with Signf=1; Supplementary Table 11) of all possible logistic regression (64 models; Supplementary Table 11) for females/males, aged 60-75 years, *APOE4* carriers. Response variable *risk* is risk of AD+. Independent variables* *dnmr*=1 (*dnmr*<50), *dnmr*=0 (*dnmr*>=50), *infs*=1 (for subjects with prior infection history during January 1, 2006 and January 1, 2016), *infs*=0 (for subjects without prior infection history during January 1, 2006 and January 1, 2016), and *age* at the baseline date January 1, 2006 as the *Age* variable. In Supplementary Table 11, a logistic regression set, having 64 models with linear terms and their pairwise interactions and corresponding to females/ males *age*d 60-75 were analyzed and presented in ascending order by AIC value. Signf=1 means that all regression coefficient were significant (P-value<0.05) in a specific model, Signf=0 means the opposite. Here and in the Supplementary Table 11, for regression model a short notation for logistic regression used. For instance, *risk* ~ 1 + *Age* + *dnmr* + *infs* + *dnmr***Age* + *infs***dnmr* denotes a standard logistic regression equation ln(*risk*/(1-*risk*)) = Intercept + b_1_**Age* + b_2_**dnmr* + b_3_**infs* + b_12_**Age***dnmr* + b_31_**infs***dnmr* where ln(x) natural logarithm, Intercept is a constant called the bias term (or intercept term), b_1_, b_2_, b_3_, b_12_, b_31_ are the regression coefficients corresponding to the *Age*, *dnmr*, *infs*, *Age***dnmr*, *infs***dnmr* terms in the regression model. The 'Model’ column shows the models presented in the short notation. The columns ‘Num’ gives the number of the model in the list arranged from smallest to largest AIC value (Supplementary Table 11). The numbers in the ‘Estimate‘ column correspond to the coefficients for the regression terms shown in the 'Model/Term’ column. The ‘Std.err’ and ‘P-Value’ presents respectively the standard error and the P-value. For all terms containing variable *Age* a unit of their measurement is 1/year; all other terms are the dimensionless quantities. Scientific notation ‘e’ means that the base number is multiplied by 10 raised to the given power.

**Supplementary Table 2.4** Regression coefficients (not all having P-value<0.05) for the logistic regression models, females/males *age*d 60-75 years, *APOE4* carriers.

| Model | Num | Model/Term | Estimate | Std.err | P-Value |
| --- | --- | --- | --- | --- | --- |
|  |  |  |  |  |  |
| *risk* ~ 1 + *Age* + *infs*:*Age* | 2 | (Intercept) | -16.207 | 1.524 | 1.99e-26 |
|  |  | *Age* | 0.193 | 0.024 | 3.30e-16 |
|  |  | *Age*:*infs* | 0.002 | 0.002 | 2.11e-01 |
| *risk* ~ 1 + *Age* + *infs* | 3 | (Intercept) | -16.237 | 1.523 | 1.60e-26 |
|  |  | *Age* | 0.194 | 0.024 | 2.73e-16 |
|  |  | *infs* | 0.155 | 0.127 | 2.23e-01 |
| *risk* ~ 1 + *Age* + *infs*:*dnmr* | 4 | (Intercept) | -16.243 | 1.525 | 1.75e-26 |
|  |  | *Age* | 0.194 | 0.024 | 2.56e-16 |
|  |  | *infs*:*dnmr* | 0.448 | 0.388 | 2.49e-01 |
| *risk* ~ 1 + *Age* + *dnmr*:*Age* | 5 | (Intercept) | -16.246 | 1.525 | 1.75e-26 |
|  |  | *Age* | 0.194 | 0.024 | 2.62e-16 |
|  |  | *Age*:*dnmr* | 0.002 | 0.003 | 4.00e-01 |
| *risk* ~ 1 + *Age* + *dnmr* | 6 | (Intercept) | -16.257 | 1.526 | 1.65e-26 |
|  |  | *Age* | 0.194 | 0.024 | 2.47e-16 |
|  |  | *dnmr* | 0.154 | 0.183 | 4.00e-01 |
| *risk* ~ 1 + *Age* + *infs* + *infs*:*Age* | 7 | (Intercept) | -15.554 | 1.690 | 3.40e-20 |
|  |  | *Age* | 0.183 | 0.026 | 3.17e-12 |
|  |  | *infs* | -3.490 | 3.938 | 3.75e-01 |
|  |  | *Age*:*infs* | 0.057 | 0.061 | 3.54e-01 |
| *risk* ~ 1 + *Age* + *dnmr*:*Age* + *infs*:*Age* | 8 | (Intercept) | -16.222 | 1.524 | 1.80e-26 |
|  |  | *Age* | 0.193 | 0.024 | 3.20e-16 |
|  |  | *Age*:*dnmr* | 0.002 | 0.003 | 4.05e-01 |
|  |  | *Age*:*infs* | 0.002 | 0.002 | 2.13e-01 |
| *risk* ~ 1 + *Age* + *dnmr* + *infs*:*Age* | 9 | (Intercept) | -16.233 | 1.524 | 1.69e-26 |
|  |  | *Age* | 0.194 | 0.024 | 3.02e-16 |
|  |  | *dnmr* | 0.153 | 0.183 | 4.05e-01 |
|  |  | *Age*:*infs* | 0.002 | 0.002 | 2.13e-01 |
| *risk* ~ 1 + *Age* + *infs*:*Age* + *infs*:*dnmr* | 10 | (Intercept) | -16.220 | 1.524 | 1.84e-26 |
|  |  | *Age* | 0.194 | 0.024 | 3.09e-16 |
|  |  | *Age*:*infs* | 0.002 | 0.002 | 3.16e-01 |
|  |  | *infs*:*dnmr* | 0.335 | 0.404 | 4.06e-01 |
| *risk* ~ 1 + *Age* + *infs* + *dnmr*:*Age* | 11 | (Intercept) | -16.252 | 1.523 | 1.44e-26 |
|  |  | *Age* | 0.194 | 0.024 | 2.65e-16 |
|  |  | *infs* | 0.154 | 0.127 | 2.25e-01 |
|  |  | *Age*:*dnmr* | 0.002 | 0.003 | 4.04e-01 |
| *risk* ~ 1 + *Age* + *dnmr* + *infs* | 12 | (Intercept) | -16.263 | 1.524 | 1.36e-26 |
|  |  | *Age* | 0.194 | 0.024 | 2.51e-16 |
|  |  | *dnmr* | 0.153 | 0.183 | 4.05e-01 |
|  |  | *infs* | 0.154 | 0.127 | 2.25e-01 |
| *risk* ~ 1 + *Age* + *infs* + *infs*:*dnmr* | 13 | (Intercept) | -16.245 | 1.524 | 1.52e-26 |
|  |  | *Age* | 0.194 | 0.024 | 2.62e-16 |
|  |  | *infs* | 0.128 | 0.132 | 3.32e-01 |
|  |  | *infs*:*dnmr* | 0.339 | 0.404 | 4.02e-01 |
| *risk* ~ 1 + *Age* + *dnmr*:*Age* + *infs*:*dnmr* | 14 | (Intercept) | -16.249 | 1.525 | 1.68e-26 |
|  |  | *Age* | 0.194 | 0.024 | 2.54e-16 |
|  |  | *Age*:*dnmr* | 0.001 | 0.003 | 6.70e-01 |
|  |  | *dnmr*:*infs* | 0.365 | 0.433 | 3.99e-01 |
| *risk* ~ 1 + *Age* + *dnmr* + *infs*:*dnmr* | 15 | (Intercept) | -16.256 | 1.526 | 1.64e-26 |
|  |  | *Age* | 0.194 | 0.024 | 2.47e-16 |
|  |  | *dnmr* | 0.087 | 0.205 | 6.70e-01 |
|  |  | *dnmr*:*infs* | 0.365 | 0.434 | 3.99e-01 |
| *risk* ~ 1 + *Age* + *infs* + *dnmr*:*Age* + *infs*:*Age* | 16 | (Intercept) | -15.569 | 1.690 | 3.13e-20 |
|  |  | *Age* | 0.183 | 0.026 | 3.10e-12 |
|  |  | *infs* | -3.495 | 3.939 | 3.75e-01 |
|  |  | *Age*:*dnmr* | 0.002 | 0.003 | 4.04e-01 |
|  |  | *Age*:*infs* | 0.057 | 0.061 | 3.53e-01 |
| *risk* ~ 1 + *Age* + *dnmr* + *infs* + *infs*:*Age* | 17 | (Intercept) | -15.580 | 1.690 | 2.97e-20 |
|  |  | *Age* | 0.183 | 0.026 | 2.96e-12 |
|  |  | *dnmr* | 0.153 | 0.183 | 4.04e-01 |
|  |  | *infs* | -3.495 | 3.939 | 3.75e-01 |
|  |  | *Age*:*infs* | 0.057 | 0.061 | 3.53e-01 |
| *risk* ~ 1 + *Age* + *infs* + *infs*:*Age* + *infs*:*dnmr* | 18 | (Intercept) | -15.554 | 1.690 | 3.40e-20 |
|  |  | *Age* | 0.183 | 0.026 | 3.17e-12 |
|  |  | *infs* | -3.568 | 3.941 | 3.65e-01 |
|  |  | *Age*:*infs* | 0.057 | 0.061 | 3.48e-01 |
|  |  | *infs*:*dnmr* | 0.345 | 0.405 | 3.93e-01 |
| *risk* ~ 1 + *Age* + *dnmr* + *dnmr*:*Age* | 19 | (Intercept) | -16.249 | 1.586 | 1.24e-24 |
|  |  | *Age* | 0.194 | 0.025 | 3.28e-15 |
|  |  | *dnmr* | 0.043 | 5.798 | 9.94e-01 |
|  |  | *Age*:*dnmr* | 0.002 | 0.090 | 9.85e-01 |
| *risk* ~ 1 + *Age* + *dnmr*:*Age* + *infs*:*Age* + *infs*:*dnmr* | 20 | (Intercept) | -16.227 | 1.524 | 1.75e-26 |
|  |  | *Age* | 0.194 | 0.024 | 3.09e-16 |
|  |  | *Age*:*dnmr* | 0.002 | 0.003 | 5.95e-01 |
|  |  | *Age*:*infs* | 0.002 | 0.002 | 2.93e-01 |
|  |  | *dnmr*:*infs* | 0.226 | 0.453 | 6.18e-01 |
| *risk* ~ 1 + *Age* + *dnmr* + *infs*:*Age* + *infs*:*dnmr* | 21 | (Intercept) | -16.235 | 1.524 | 1.68e-26 |
|  |  | *Age* | 0.194 | 0.024 | 2.96e-16 |
|  |  | *dnmr* | 0.109 | 0.206 | 5.96e-01 |
|  |  | *Age*:*infs* | 0.002 | 0.002 | 2.93e-01 |
|  |  | *dnmr*:*infs* | 0.226 | 0.453 | 6.18e-01 |
| *risk* ~ 1 + *Age* + *infs* + *dnmr*:*Age* + *infs*:*dnmr* | 22 | (Intercept) | -16.253 | 1.524 | 1.44e-26 |
|  |  | *Age* | 0.194 | 0.024 | 2.60e-16 |
|  |  | *infs* | 0.135 | 0.133 | 3.08e-01 |
|  |  | *Age*:*dnmr* | 0.002 | 0.003 | 5.98e-01 |
|  |  | *infs*:*dnmr* | 0.230 | 0.453 | 6.12e-01 |
| *risk* ~ 1 + *Age* + *dnmr* + *infs* + *infs*:*dnmr* | 23 | (Intercept) | -16.261 | 1.524 | 1.39e-26 |
|  |  | *Age* | 0.194 | 0.024 | 2.50e-16 |
|  |  | *dnmr* | 0.109 | 0.206 | 5.98e-01 |
|  |  | *infs* | 0.135 | 0.133 | 3.08e-01 |
|  |  | *dnmr*:*infs* | 0.230 | 0.453 | 6.12e-01 |
| *risk* ~ 1 + *Age* + *dnmr* + *dnmr*:*Age* + *infs*:*Age* | 24 | (Intercept) | -16.224 | 1.584 | 1.27e-24 |
|  |  | *Age* | 0.193 | 0.025 | 3.95e-15 |
|  |  | *dnmr* | 0.033 | 5.793 | 9.95e-01 |
|  |  | *Age*:*dnmr* | 0.002 | 0.090 | 9.83e-01 |
|  |  | *Age*:*infs* | 0.002 | 0.002 | 2.13e-01 |
| *risk* ~ 1 + *Age* + *dnmr* + *infs* + *dnmr*:*Age* | 25 | (Intercept) | -16.254 | 1.584 | 1.04e-24 |
|  |  | *Age* | 0.194 | 0.025 | 3.32e-15 |
|  |  | *dnmr* | 0.033 | 5.793 | 9.96e-01 |
|  |  | *infs* | 0.154 | 0.127 | 2.25e-01 |
|  |  | *Age*:*dnmr* | 0.002 | 0.090 | 9.83e-01 |
| *risk* ~ 1 + *Age* + *dnmr* + *dnmr*:*Age* + *infs*:*dnmr* | 26 | (Intercept) | -16.249 | 1.586 | 1.24e-24 |
|  |  | *Age* | 0.194 | 0.025 | 3.28e-15 |
|  |  | *dnmr* | -0.003 | 5.788 | 1.00e+00 |
|  |  | *Age*:*dnmr* | 0.001 | 0.090 | 9.88e-01 |
|  |  | *dnmr*:*infs* | 0.365 | 0.434 | 3.99e-01 |
| *risk* ~ 1 + *Age* + *dnmr* + *infs* + *infs*:*Age* + *infs*:*dnmr* | 27 | (Intercept) | -15.572 | 1.690 | 3.12e-20 |
|  |  | *Age* | 0.183 | 0.026 | 3.03e-12 |
|  |  | *dnmr* | 0.108 | 0.206 | 6.01e-01 |
|  |  | *infs* | -3.550 | 3.941 | 3.68e-01 |
|  |  | *Age*:*infs* | 0.057 | 0.061 | 3.49e-01 |
|  |  | *dnmr*:*infs* | 0.238 | 0.454 | 6.01e-01 |
| *risk* ~ 1 + *Age* + *infs* + *dnmr*:*Age* + *infs*:*Age* + *infs*:*dnmr* | 28 | (Intercept) | -15.564 | 1.690 | 3.22e-20 |
|  |  | *Age* | 0.183 | 0.026 | 3.12e-12 |
|  |  | *infs* | -3.548 | 3.941 | 3.68e-01 |
|  |  | *Age*:*dnmr* | 0.002 | 0.003 | 6.02e-01 |
|  |  | *Age*:*infs* | 0.057 | 0.061 | 3.49e-01 |
|  |  | *infs*:*dnmr* | 0.237 | 0.454 | 6.01e-01 |
| *risk* ~ 1 + *Age* + *dnmr* + *infs* + *dnmr*:*Age* + *infs*:*Age* | 29 | (Intercept) | -15.572 | 1.744 | 4.31e-19 |
|  |  | *Age* | 0.183 | 0.027 | 1.40e-11 |
|  |  | *dnmr* | 0.041 | 5.795 | 9.94e-01 |
|  |  | *infs* | -3.495 | 3.939 | 3.75e-01 |
|  |  | *Age*:*dnmr* | 0.002 | 0.090 | 9.85e-01 |
|  |  | *Age*:*infs* | 0.057 | 0.061 | 3.53e-01 |
| *risk* ~ 1 + *Age* + *dnmr* + *dnmr*:*Age* + *infs*:*Age* + *infs*:*dnmr* | 30 | (Intercept) | -16.227 | 1.584 | 1.27e-24 |
|  |  | *Age* | 0.194 | 0.025 | 3.88e-15 |
|  |  | *dnmr* | 0.006 | 5.788 | 9.99e-01 |
|  |  | *Age*:*dnmr* | 0.002 | 0.090 | 9.86e-01 |
|  |  | *Age*:*infs* | 0.002 | 0.002 | 2.93e-01 |
|  |  | *dnmr*:*infs* | 0.226 | 0.453 | 6.18e-01 |
| *risk* ~ 1 + *Age* + *dnmr* + *infs* + *dnmr*:*Age* + *infs*:*dnmr* | 31 | (Intercept) | -16.253 | 1.584 | 1.06e-24 |
|  |  | *Age* | 0.194 | 0.025 | 3.32e-15 |
|  |  | *dnmr* | 0.001 | 5.788 | 1.00e+00 |
|  |  | *infs* | 0.135 | 0.133 | 3.08e-01 |
|  |  | *Age*:*dnmr* | 0.002 | 0.090 | 9.85e-01 |
|  |  | *dnmr*:*infs* | 0.230 | 0.453 | 6.12e-01 |
| *risk* ~ 1 + *Age* + *dnmr* + *infs* + *dnmr*:*Age* + *infs*:*Age* + *infs*:*dnmr* | 32 | (Intercept) | -15.574 | 1.740 | 3.52e-19 |
|  |  | *Age* | 0.183 | 0.027 | 1.23e-11 |
|  |  | *dnmr* | 0.140 | 5.795 | 9.81e-01 |
|  |  | *infs* | -3.550 | 3.943 | 3.68e-01 |
|  |  | *Age*:*dnmr* | -0.001 | 0.090 | 9.95e-01 |
|  |  | *Age*:*infs* | 0.057 | 0.061 | 3.49e-01 |
|  |  | *dnmr*:*infs* | 0.238 | 0.455 | 6.01e-01 |
| *risk* ~ 1 + *dnmr* + *infs* + *dnmr*:*Age* + *infs*:*Age* | 34 | (Intercept) | -3.854 | 0.054 | 0.00e+00 |
|  |  | *dnmr* | -9.461 | 5.602 | 9.13e-02 |
|  |  | *infs* | -14.481 | 3.583 | 5.30e-05 |
|  |  | *dnmr*:*Age* | 0.150 | 0.087 | 8.51e-02 |
|  |  | *infs*:*Age* | 0.229 | 0.055 | 3.82e-05 |
| *risk* ~ 1 + *infs* + *infs*:*Age* + *infs*:*dnmr* | 35 | (Intercept) | -3.846 | 0.052 | 0.00e+00 |
|  |  | *infs* | -15.275 | 3.560 | 1.78e-05 |
|  |  | *infs*:*Age* | 0.241 | 0.055 | 1.27e-05 |
|  |  | *infs*:*dnmr* | 0.345 | 0.405 | 3.93e-01 |
| *risk* ~ 1 + *infs* + *dnmr*:*Age* + *infs*:*Age* | 36 | (Intercept) | -3.856 | 0.054 | 0.00e+00 |
|  |  | *infs* | -15.207 | 3.558 | 1.92e-05 |
|  |  | *dnmr*:*Age* | 0.002 | 0.003 | 4.20e-01 |
|  |  | *infs*:*Age* | 0.240 | 0.055 | 1.33e-05 |
| *risk* ~ 1 + *dnmr* + *infs* + *infs*:*Age* | 37 | (Intercept) | -3.856 | 0.054 | 0.00e+00 |
|  |  | *dnmr* | 0.137 | 0.183 | 4.55e-01 |
|  |  | *infs* | -15.216 | 3.558 | 1.89e-05 |
|  |  | *infs*:*Age* | 0.240 | 0.055 | 1.31e-05 |
| *risk* ~ 1 + *dnmr* + *infs* + *dnmr*:*Age* + *infs*:*Age* + *infs*:*dnmr* | 38 | (Intercept) | -3.852 | 0.054 | 0.00e+00 |
|  |  | *dnmr* | -9.239 | 5.672 | 1.03e-01 |
|  |  | *infs* | -14.463 | 3.589 | 5.57e-05 |
|  |  | *dnmr*:*Age* | 0.146 | 0.089 | 9.84e-02 |
|  |  | *infs*:*Age* | 0.228 | 0.056 | 4.10e-05 |
|  |  | *dnmr*:*infs* | 0.123 | 0.463 | 7.90e-01 |
| *risk* ~ 1 + *infs* + *dnmr*:*Age* + *infs*:*Age* + *infs*:*dnmr* | 39 | (Intercept) | -3.853 | 0.054 | 0.00e+00 |
|  |  | *infs* | -15.260 | 3.560 | 1.82e-05 |
|  |  | *dnmr*:*Age* | 0.002 | 0.003 | 6.24e-01 |
|  |  | *infs*:*Age* | 0.240 | 0.055 | 1.29e-05 |
|  |  | *infs*:*dnmr* | 0.244 | 0.455 | 5.92e-01 |
| *risk* ~ 1 + *dnmr* + *infs* + *infs*:*Age* + *infs*:*dnmr* | 40 | (Intercept) | -3.852 | 0.054 | 0.00e+00 |
|  |  | *dnmr* | 0.088 | 0.206 | 6.68e-01 |
|  |  | *infs* | -15.269 | 3.560 | 1.80e-05 |
|  |  | *infs*:*Age* | 0.241 | 0.055 | 1.27e-05 |
|  |  | *dnmr*:*infs* | 0.257 | 0.454 | 5.71e-01 |
| *risk* ~ 1 + *dnmr* + *dnmr*:*Age* + *infs*:*Age* | 42 | (Intercept) | -3.858 | 0.054 | 0.00e+00 |
|  |  | *dnmr* | -12.332 | 5.572 | 2.69e-02 |
|  |  | *dnmr*:*Age* | 0.195 | 0.087 | 2.44e-02 |
|  |  | *Age*:*infs* | 0.003 | 0.002 | 1.69e-01 |
| *risk* ~ 1 + *dnmr* + *infs* + *dnmr*:*Age* | 43 | (Intercept) | -3.855 | 0.054 | 0.00e+00 |
|  |  | *dnmr* | -12.366 | 5.572 | 2.65e-02 |
|  |  | *infs* | 0.154 | 0.127 | 2.25e-01 |
|  |  | *dnmr*:*Age* | 0.196 | 0.087 | 2.40e-02 |
| *risk* ~ 1 + *dnmr* + *dnmr*:*Age* + *infs*:*dnmr* | 44 | (Intercept) | -3.831 | 0.049 | 0.00e+00 |
|  |  | *dnmr* | -12.422 | 5.566 | 2.56e-02 |
|  |  | *dnmr*:*Age* | 0.196 | 0.087 | 2.39e-02 |
|  |  | *dnmr*:*infs* | 0.365 | 0.434 | 3.99e-01 |
| *risk* ~ 1 + *infs*:*Age* | 46 | (Intercept) | -3.850 | 0.052 | 0.00e+00 |
|  |  | *infs*:*Age* | 0.003 | 0.002 | 1.65e-01 |
| *risk* ~ 1 + *dnmr* + *dnmr*:*Age* + *infs*:*Age* + *infs*:*dnmr* | 47 | (Intercept) | -3.856 | 0.054 | 0.00e+00 |
|  |  | *dnmr* | -12.361 | 5.567 | 2.64e-02 |
|  |  | *dnmr*:*Age* | 0.195 | 0.087 | 2.43e-02 |
|  |  | *Age*:*infs* | 0.002 | 0.002 | 2.36e-01 |
|  |  | *dnmr*:*infs* | 0.207 | 0.453 | 6.47e-01 |
| *risk* ~ 1 + *infs* | 48 | (Intercept) | -3.846 | 0.052 | 0.00e+00 |
|  |  | *infs* | 0.154 | 0.127 | 2.23e-01 |
| *risk* ~ 1 + *dnmr* + *infs* + *dnmr*:*Age* + *infs*:*dnmr* | 49 | (Intercept) | -3.852 | 0.054 | 0.00e+00 |
|  |  | *dnmr* | -12.400 | 5.566 | 2.59e-02 |
|  |  | *infs* | 0.135 | 0.133 | 3.07e-01 |
|  |  | *dnmr*:*Age* | 0.196 | 0.087 | 2.39e-02 |
|  |  | *dnmr*:*infs* | 0.230 | 0.453 | 6.12e-01 |
| *risk* ~ 1 + *infs*:*dnmr* | 50 | (Intercept) | -3.827 | 0.048 | 0.00e+00 |
|  |  | *infs*:*dnmr* | 0.426 | 0.387 | 2.72e-01 |
| *risk* ~ 1 + *dnmr*:*Age* | 51 | (Intercept) | -3.832 | 0.050 | 0.00e+00 |
|  |  | *dnmr*:*Age* | 0.002 | 0.003 | 4.20e-01 |
| *risk* ~ 1 + *dnmr* | 52 | (Intercept) | -3.831 | 0.049 | 0.00e+00 |
|  |  | *dnmr* | 0.133 | 0.183 | 4.66e-01 |
| *risk* ~ 1 + *dnmr*:*Age* + *infs*:*Age* | 53 | (Intercept) | -3.860 | 0.054 | 0.00e+00 |
|  |  | *dnmr*:*Age* | 0.002 | 0.003 | 4.25e-01 |
|  |  | *Age*:*infs* | 0.003 | 0.002 | 1.66e-01 |
| *risk* ~ 1 + *infs*:*Age* + *infs*:*dnmr* | 54 | (Intercept) | -3.850 | 0.052 | 0.00e+00 |
|  |  | *infs*:*Age* | 0.002 | 0.002 | 2.46e-01 |
|  |  | *infs*:*dnmr* | 0.297 | 0.402 | 4.60e-01 |
| *risk* ~ 1 + *dnmr* + *infs*:*Age* | 55 | (Intercept) | -3.859 | 0.054 | 0.00e+00 |
|  |  | *dnmr* | 0.132 | 0.183 | 4.71e-01 |
|  |  | *infs*:*Age* | 0.003 | 0.002 | 1.66e-01 |
| *risk* ~ 1 + *infs* + *dnmr*:*Age* | 56 | (Intercept) | -3.856 | 0.054 | 0.00e+00 |
|  |  | *infs* | 0.154 | 0.127 | 2.25e-01 |
|  |  | *dnmr*:*Age* | 0.002 | 0.003 | 4.25e-01 |
| *risk* ~ 1 + *infs* + *infs*:*dnmr* | 57 | (Intercept) | -3.846 | 0.052 | 0.00e+00 |
|  |  | *infs* | 0.129 | 0.132 | 3.26e-01 |
|  |  | *infs*:*dnmr* | 0.316 | 0.403 | 4.33e-01 |
| *risk* ~ 1 + *dnmr* + *infs* | 58 | (Intercept) | -3.855 | 0.054 | 0.00e+00 |
|  |  | *dnmr* | 0.132 | 0.183 | 4.71e-01 |
|  |  | *infs* | 0.154 | 0.127 | 2.25e-01 |
| *risk* ~ 1 + *dnmr*:*Age* + *infs*:*dnmr* | 59 | (Intercept) | -3.832 | 0.050 | 0.00e+00 |
|  |  | *dnmr*:*Age* | 0.001 | 0.003 | 6.81e-01 |
|  |  | *dnmr*:*infs* | 0.347 | 0.432 | 4.21e-01 |
| *risk* ~ 1 + *dnmr* + *infs*:*dnmr* | 60 | (Intercept) | -3.831 | 0.049 | 0.00e+00 |
|  |  | *dnmr* | 0.067 | 0.204 | 7.44e-01 |
|  |  | *dnmr*:*infs* | 0.363 | 0.432 | 4.02e-01 |
| *risk* ~ 1 + *dnmr*:*Age* + *infs*:*Age* + *infs*:*dnmr* | 61 | (Intercept) | -3.857 | 0.054 | 0.00e+00 |
|  |  | *dnmr*:*Age* | 0.002 | 0.003 | 5.94e-01 |
|  |  | *Age*:*infs* | 0.003 | 0.002 | 2.27e-01 |
|  |  | *dnmr*:*infs* | 0.189 | 0.451 | 6.76e-01 |
| *risk* ~ 1 + *dnmr* + *infs*:*Age* + *infs*:*dnmr* | 62 | (Intercept) | -3.856 | 0.054 | 0.00e+00 |
|  |  | *dnmr* | 0.092 | 0.206 | 6.55e-01 |
|  |  | *infs*:*Age* | 0.002 | 0.002 | 2.31e-01 |
|  |  | *dnmr*:*infs* | 0.205 | 0.452 | 6.50e-01 |
| *risk* ~ 1 + *infs* + *dnmr*:*Age* + *infs*:*dnmr* | 63 | (Intercept) | -3.853 | 0.054 | 0.00e+00 |
|  |  | *infs* | 0.137 | 0.133 | 3.03e-01 |
|  |  | *dnmr*:*Age* | 0.002 | 0.003 | 6.07e-01 |
|  |  | *infs*:*dnmr* | 0.211 | 0.452 | 6.41e-01 |
| *risk* ~ 1 + *dnmr* + *infs* + *infs*:*dnmr* | 64 | (Intercept) | -3.852 | 0.054 | 0.00e+00 |
|  |  | *dnmr* | 0.088 | 0.206 | 6.68e-01 |
|  |  | *infs* | 0.135 | 0.133 | 3.07e-01 |
|  |  | *dnmr*:*infs* | 0.227 | 0.452 | 6.15e-01 |

**Note:** here, we presented the regressions coefficients obtained from the regression analysis for the subset (43 models with Signf=0; Supplementary Table 11) of all possible logistic regression (64 models; Supplementary Table 11) for females/males, aged 60-75 years, *APOE4* carriers. Response variable *risk* is risk of AD+. Independent variables* *dnmr*=1 (*dnmr*<50), *dnmr*=0 (*dnmr*>=50), *infs*=1 (for subjects with prior infection history during January 1, 2006 and January 1, 2016), *infs*=0 (for subjects without prior infection history during January 1, 2006 and January 1, 2016), and *age* at the baseline date January 1, 2006 as the *Age* variable. In Supplementary Table 11, a logistic regression set, having 64 models with linear terms and their pairwise interactions and corresponding to females/ males *age*d 60-75 were analyzed and presented in ascending order by AIC value. Signf=1 means that all regression coefficient were significant (P-value<0.05) in a specific model, Signf=0 means the opposite. Here and in the Supplementary Table 11, for regression model a short notation for logistic regression used. For instance, *risk* ~ 1 + *Age* + *dnmr* + *infs* + *dnmr***Age* + *infs***dnmr* denotes a standard logistic regression equation ln(*risk*/(1-*risk*)) = Intercept + b_1_**Age* + b_2_**dnmr* + b_3_**infs* + b_12_**Age***dnmr* + b_31_**infs***dnmr* where ln(x) natural logarithm, Intercept is a constant called the bias term (or intercept term), b_1_, b_2_, b_3_, b_12_, b_31_ are the regression coefficients corresponding to the *Age*, *dnmr*, *infs*, *Age***dnmr*, *infs***dnmr* terms in the regression model. The 'Model’ column shows the models presented in the short notation. The columns ‘Num’ gives the number of the model in the list arranged from smallest to largest AIC value (Supplementary Table 11). The numbers in the ‘Estimate‘ column correspond to the coefficients for the regression terms shown in the 'Model/Term’ column. The ‘Std.err’ and ‘P-Value’ presents respectively the standard error and the P-value. For all terms containing variable *Age* a unit of their measurement is 1/year; all other terms are the dimensionless quantities. Scientific notation ‘e’ means that the base number is multiplied by 10 raised to the given power.

**Supplementary Table 2.5** Regression coefficients (all having P-value<0.05) for the logistic regression models, females/males *age*d 60-75 years, *APOE4* non-carriers.

| Model | Num | Model/Term | Estimate | Std.err | P-Value |
| --- | --- | --- | --- | --- | --- |
|  |  |  |  |  |  |
| *risk* ~ 1 + *Age* + *infs* + *infs*:*dnmr* | 2 | (Intercept) | -16.112 | 1.592 | 4.59e-24 |
|  |  | *Age* | 0.172 | 0.025 | 3.67e-12 |
|  |  | *infs* | 0.725 | 0.116 | 3.89e-10 |
|  |  | *infs*:*dnmr* | 0.800 | 0.280 | 4.35e-03 |
| *risk* ~ 1 + *Age* + *infs*:*Age* + *infs*:*dnmr* | 3 | (Intercept) | -15.888 | 1.593 | 2.00e-23 |
|  |  | *Age* | 0.169 | 0.025 | 1.03e-11 |
|  |  | *Age*:*infs* | 0.011 | 0.002 | 6.00e-10 |
|  |  | *infs*:*dnmr* | 0.805 | 0.281 | 4.13e-03 |
| *risk* ~ 1 + *Age* + *infs* | 14 | (Intercept) | -16.124 | 1.592 | 4.25e-24 |
|  |  | *Age* | 0.172 | 0.025 | 3.47e-12 |
|  |  | *infs* | 0.798 | 0.110 | 4.96e-13 |
| *risk* ~ 1 + *Age* + *infs*:*Age* | 17 | (Intercept) | -15.878 | 1.593 | 2.15e-23 |
|  |  | *Age* | 0.168 | 0.025 | 1.08e-11 |
|  |  | *Age*:*infs* | 0.012 | 0.002 | 7.95e-13 |
| *risk* ~ 1 + *Age* + *infs*:*dnmr* | 25 | (Intercept) | -16.046 | 1.600 | 1.13e-23 |
|  |  | *Age* | 0.173 | 0.025 | 3.19e-12 |
|  |  | *infs*:*dnmr* | 1.376 | 0.267 | 2.62e-07 |
| *risk* ~ 1 + *infs* + *infs*:*Age* + *infs*:*dnmr* | 29 | (Intercept) | -5.112 | 0.060 | 0.00e+00 |
|  |  | *infs* | -6.552 | 2.813 | 1.99e-02 |
|  |  | *infs*:*Age* | 0.114 | 0.044 | 9.33e-03 |
|  |  | *infs*:*dnmr* | 0.801 | 0.280 | 4.22e-03 |
| *risk* ~ 1 + *infs*:*Age* + *infs*:*dnmr* | 32 | (Intercept) | -5.115 | 0.060 | 0.00e+00 |
|  |  | *infs*:*Age* | 0.012 | 0.002 | 1.57e-10 |
|  |  | *infs*:*dnmr* | 0.797 | 0.280 | 4.39e-03 |
| *risk* ~ 1 + *infs* + *infs*:*Age* | 34 | (Intercept) | -5.112 | 0.060 | 0.00e+00 |
|  |  | *infs* | -6.518 | 2.814 | 2.05e-02 |
|  |  | *infs*:*Age* | 0.115 | 0.044 | 8.97e-03 |
| *risk* ~ 1 + *infs* + *infs*:*dnmr* | 35 | (Intercept) | -5.112 | 0.060 | 0.00e+00 |
|  |  | *infs* | 0.727 | 0.116 | 3.45e-10 |
|  |  | *infs*:*dnmr* | 0.806 | 0.280 | 4.00e-03 |
| *risk* ~ 1 + *Age* | 43 | (Intercept) | -16.053 | 1.601 | 1.19e-23 |
|  |  | *Age* | 0.174 | 0.025 | 2.86e-12 |
| *risk* ~ 1 + *infs*:*Age* | 47 | (Intercept) | -5.115 | 0.060 | 0.00e+00 |
|  |  | *infs*:*Age* | 0.013 | 0.002 | 1.76e-13 |
| *risk* ~ 1 + *infs* | 52 | (Intercept) | -5.112 | 0.060 | 0.00e+00 |
|  |  | *infs* | 0.800 | 0.110 | 4.15e-13 |
| *risk* ~ 1 + *infs*:*dnmr* | 57 | (Intercept) | -4.963 | 0.051 | 0.00e+00 |
|  |  | *infs*:*dnmr* | 1.383 | 0.267 | 2.15e-07 |
| *risk* ~ 1 | 61 | (Intercept) | -4.935 | 0.050 | 0.00e+00 |

**Note:** here, we presented the regressions coefficients obtained from the regression analysis for the subset (21 models with Signf=1; Supplementary Table 11) of all possible logistic regression (64 models; Supplementary Table 11) for females/males, aged 60-75 years, *APOE4* non-carriers. Response variable *risk* is risk of AD+. Independent variables* *dnmr*=1 (*dnmr*<50), *dnmr*=0 (*dnmr*>=50), *infs*=1 (for subjects with prior infection history during January 1, 2006 and January 1, 2016), *infs*=0 (for subjects without prior infection history during January 1, 2006 and January 1, 2016), and *age* at the baseline date January 1, 2006 as the *Age* variable. In Supplementary Table 11, a logistic regression set, having 64 models with linear terms and their pairwise interactions and corresponding to females/ males *age*d 60-75 were analyzed and presented in ascending order by AIC value. Signf=1 means that all regression coefficient were significant (P-value<0.05) in a specific model, Signf=0 means the opposite. Here and in the Supplementary Table 11, for regression model a short notation for logistic regression used. For instance, *risk* ~ 1 + *Age* + *dnmr* + *infs* + *dnmr***Age* + *infs***dnmr* denotes a standard logistic regression equation ln(*risk*/(1-*risk*)) = Intercept + b_1_**Age* + b_2_**dnmr* + b_3_**infs* + b_12_**Age***dnmr* + b_31_**infs***dnmr* where ln(x) natural logarithm, Intercept is a constant called the bias term (or intercept term), b_1_, b_2_, b_3_, b_12_, b_31_ are the regression coefficients corresponding to the *Age*, *dnmr*, *infs*, *Age***dnmr*, *infs***dnmr* terms in the regression model. The 'Model’ column shows the models presented in the short notation. The columns ‘Num’ gives the number of the model in the list arranged from smallest to largest AIC value (Supplementary Table 11). The numbers in the ‘Estimate‘ column correspond to the coefficients for the regression terms shown in the 'Model/Term’ column. The ‘Std.err’ and ‘P-Value’ presents respectively the standard error and the P-value. For all terms containing variable *Age* a unit of their measurement is 1/year; all other terms are the dimensionless quantities. Scientific notation ‘e’ means that the base number is multiplied by 10 raised to the given power.

**Supplementary Table 2.6** Regression coefficients (not all having P-value<0.05) for the logistic regression models, females/males *age*d 60-75 years, *APOE4* non-carriers.

| Model | Num | Model/Term | Estimate | Std.err | P-Value |
| --- | --- | --- | --- | --- | --- |
|  |  |  |  |  |  |
| *risk* ~ 1 + *Age* + *infs* + *infs*:*Age* + *infs*:*dnmr* | 1 | (Intercept) | -17.831 | 1.929 | 2.34e-20 |
|  |  | *Age* | 0.199 | 0.030 | 3.17e-11 |
|  |  | *infs* | 6.167 | 3.410 | 7.06e-02 |
|  |  | *Age*:*infs* | -0.085 | 0.053 | 1.11e-01 |
|  |  | *infs*:*dnmr* | 0.801 | 0.280 | 4.22e-03 |
| *risk* ~ 1 + *Age* + *infs* + *dnmr*:*Age* + *infs*:*Age* + *infs*:*dnmr* | 4 | (Intercept) | -17.829 | 1.929 | 2.36e-20 |
|  |  | *Age* | 0.199 | 0.030 | 3.08e-11 |
|  |  | *infs* | 6.143 | 3.411 | 7.17e-02 |
|  |  | *Age*:*dnmr* | -0.003 | 0.004 | 5.24e-01 |
|  |  | *Age*:*infs* | -0.084 | 0.053 | 1.12e-01 |
|  |  | *infs*:*dnmr* | 0.971 | 0.386 | 1.20e-02 |
| *risk* ~ 1 + *Age* + *dnmr* + *infs* + *infs*:*Age* + *infs*:*dnmr* | 5 | (Intercept) | -17.820 | 1.929 | 2.46e-20 |
|  |  | *Age* | 0.199 | 0.030 | 3.18e-11 |
|  |  | *dnmr* | -0.158 | 0.266 | 5.53e-01 |
|  |  | *infs* | 6.156 | 3.410 | 7.10e-02 |
|  |  | *Age*:*infs* | -0.085 | 0.053 | 1.11e-01 |
|  |  | *dnmr*:*infs* | 0.960 | 0.386 | 1.30e-02 |
| *risk* ~ 1 + *Age* + *infs* + *dnmr*:*Age* + *infs*:*dnmr* | 6 | (Intercept) | -16.114 | 1.592 | 4.51e-24 |
|  |  | *Age* | 0.172 | 0.025 | 3.48e-12 |
|  |  | *infs* | 0.715 | 0.117 | 8.93e-10 |
|  |  | *Age*:*dnmr* | -0.003 | 0.004 | 5.18e-01 |
|  |  | *infs*:*dnmr* | 0.973 | 0.388 | 1.21e-02 |
| *risk* ~ 1 + *Age* + *dnmr* + *infs* + *infs*:*dnmr* | 7 | (Intercept) | -16.102 | 1.592 | 4.90e-24 |
|  |  | *Age* | 0.172 | 0.025 | 3.68e-12 |
|  |  | *dnmr* | -0.158 | 0.266 | 5.52e-01 |
|  |  | *infs* | 0.716 | 0.117 | 8.59e-10 |
|  |  | *dnmr*:*infs* | 0.958 | 0.387 | 1.32e-02 |
| *risk* ~ 1 + *Age* + *dnmr* + *infs* + *dnmr*:*Age* + *infs*:*Age* + *infs*:*dnmr* | 8 | (Intercept) | -18.169 | 1.957 | 1.62e-20 |
|  |  | *Age* | 0.204 | 0.030 | 1.79e-11 |
|  |  | *dnmr* | 6.360 | 6.001 | 2.89e-01 |
|  |  | *infs* | 5.673 | 3.445 | 9.96e-02 |
|  |  | *Age*:*dnmr* | -0.102 | 0.094 | 2.78e-01 |
|  |  | *Age*:*infs* | -0.077 | 0.054 | 1.50e-01 |
|  |  | *dnmr*:*infs* | 0.933 | 0.388 | 1.61e-02 |
| *risk* ~ 1 + *Age* + *dnmr* + *infs* + *dnmr*:*Age* + *infs*:*dnmr* | 9 | (Intercept) | -16.692 | 1.660 | 8.43e-24 |
|  |  | *Age* | 0.181 | 0.026 | 2.10e-12 |
|  |  | *dnmr* | 7.489 | 5.944 | 2.08e-01 |
|  |  | *infs* | 0.716 | 0.117 | 8.80e-10 |
|  |  | *Age*:*dnmr* | -0.119 | 0.093 | 1.99e-01 |
|  |  | *dnmr*:*infs* | 0.965 | 0.386 | 1.25e-02 |
| *risk* ~ 1 + *Age* + *dnmr*:*Age* + *infs*:*Age* + *infs*:*dnmr* | 10 | (Intercept) | -15.893 | 1.593 | 1.93e-23 |
|  |  | *Age* | 0.169 | 0.025 | 9.66e-12 |
|  |  | *Age*:*dnmr* | -0.003 | 0.004 | 5.12e-01 |
|  |  | *Age*:*infs* | 0.011 | 0.002 | 1.36e-09 |
|  |  | *dnmr*:*infs* | 0.980 | 0.388 | 1.15e-02 |
| *risk* ~ 1 + *Age* + *dnmr* + *infs*:*Age* + *infs*:*dnmr* | 11 | (Intercept) | -15.881 | 1.593 | 2.10e-23 |
|  |  | *Age* | 0.169 | 0.025 | 1.02e-11 |
|  |  | *dnmr* | -0.160 | 0.266 | 5.47e-01 |
|  |  | *Age*:*infs* | 0.011 | 0.002 | 1.31e-09 |
|  |  | *dnmr*:*infs* | 0.965 | 0.387 | 1.26e-02 |
| *risk* ~ 1 + *Age* + *dnmr* + *dnmr*:*Age* + *infs*:*Age* + *infs*:*dnmr* | 12 | (Intercept) | -16.483 | 1.660 | 3.13e-23 |
|  |  | *Age* | 0.178 | 0.026 | 5.36e-12 |
|  |  | *dnmr* | 7.654 | 5.944 | 1.98e-01 |
|  |  | *Age*:*dnmr* | -0.122 | 0.093 | 1.90e-01 |
|  |  | *Age*:*infs* | 0.011 | 0.002 | 1.29e-09 |
|  |  | *dnmr*:*infs* | 0.977 | 0.386 | 1.14e-02 |
| *risk* ~ 1 + *Age* + *infs* + *infs*:*Age* | 13 | (Intercept) | -17.831 | 1.929 | 2.34e-20 |
|  |  | *Age* | 0.199 | 0.030 | 3.17e-11 |
|  |  | *infs* | 6.201 | 3.411 | 6.90e-02 |
|  |  | *Age*:*infs* | -0.084 | 0.053 | 1.13e-01 |
| *risk* ~ 1 + *Age* + *dnmr* + *infs* + *infs*:*Age* | 15 | (Intercept) | -17.847 | 1.929 | 2.18e-20 |
|  |  | *Age* | 0.199 | 0.030 | 3.15e-11 |
|  |  | *dnmr* | 0.209 | 0.191 | 2.73e-01 |
|  |  | *infs* | 6.212 | 3.410 | 6.85e-02 |
|  |  | *Age*:*infs* | -0.084 | 0.053 | 1.13e-01 |
| *risk* ~ 1 + *Age* + *infs* + *dnmr*:*Age* + *infs*:*Age* | 16 | (Intercept) | -17.832 | 1.929 | 2.34e-20 |
|  |  | *Age* | 0.199 | 0.030 | 3.33e-11 |
|  |  | *infs* | 6.212 | 3.410 | 6.85e-02 |
|  |  | *Age*:*dnmr* | 0.003 | 0.003 | 2.93e-01 |
|  |  | *Age*:*infs* | -0.084 | 0.053 | 1.13e-01 |
| *risk* ~ 1 + *Age* + *dnmr* + *infs* + *dnmr*:*Age* + *infs*:*Age* | 18 | (Intercept) | -18.399 | 1.980 | 1.52e-20 |
|  |  | *Age* | 0.207 | 0.031 | 1.50e-11 |
|  |  | *dnmr* | 7.622 | 5.983 | 2.03e-01 |
|  |  | *infs* | 6.183 | 3.408 | 6.96e-02 |
|  |  | *Age*:*dnmr* | -0.116 | 0.094 | 2.17e-01 |
|  |  | *Age*:*infs* | -0.084 | 0.053 | 1.14e-01 |
| *risk* ~ 1 + *Age* + *dnmr* + *infs* | 19 | (Intercept) | -16.137 | 1.593 | 3.94e-24 |
|  |  | *Age* | 0.172 | 0.025 | 3.50e-12 |
|  |  | *dnmr* | 0.208 | 0.191 | 2.76e-01 |
|  |  | *infs* | 0.798 | 0.110 | 4.99e-13 |
| *risk* ~ 1 + *Age* + *infs* + *dnmr*:*Age* | 20 | (Intercept) | -16.122 | 1.592 | 4.34e-24 |
|  |  | *Age* | 0.172 | 0.025 | 3.75e-12 |
|  |  | *infs* | 0.798 | 0.110 | 4.99e-13 |
|  |  | *Age*:*dnmr* | 0.003 | 0.003 | 2.95e-01 |
| *risk* ~ 1 + *Age* + *dnmr* + *infs* + *dnmr*:*Age* | 21 | (Intercept) | -16.704 | 1.659 | 7.36e-24 |
|  |  | *Age* | 0.181 | 0.026 | 2.14e-12 |
|  |  | *dnmr* | 7.702 | 6.004 | 2.00e-01 |
|  |  | *infs* | 0.798 | 0.110 | 4.90e-13 |
|  |  | *Age*:*dnmr* | -0.117 | 0.094 | 2.13e-01 |
| *risk* ~ 1 + *Age* + *dnmr* + *infs*:*Age* | 22 | (Intercept) | -15.890 | 1.593 | 2.00e-23 |
|  |  | *Age* | 0.168 | 0.025 | 1.09e-11 |
|  |  | *dnmr* | 0.208 | 0.191 | 2.76e-01 |
|  |  | *Age*:*infs* | 0.012 | 0.002 | 8.02e-13 |
| *risk* ~ 1 + *Age* + *dnmr*:*Age* + *infs*:*Age* | 23 | (Intercept) | -15.875 | 1.593 | 2.19e-23 |
|  |  | *Age* | 0.168 | 0.025 | 1.17e-11 |
|  |  | *Age*:*dnmr* | 0.003 | 0.003 | 2.96e-01 |
|  |  | *Age*:*infs* | 0.012 | 0.002 | 8.02e-13 |
| *risk* ~ 1 + *Age* + *dnmr* + *dnmr*:*Age* + *infs*:*Age* | 24 | (Intercept) | -16.458 | 1.659 | 3.45e-23 |
|  |  | *Age* | 0.177 | 0.026 | 6.43e-12 |
|  |  | *dnmr* | 7.710 | 6.006 | 1.99e-01 |
|  |  | *Age*:*dnmr* | -0.117 | 0.094 | 2.13e-01 |
|  |  | *Age*:*infs* | 0.012 | 0.002 | 7.86e-13 |
| *risk* ~ 1 + *Age* + *dnmr*:*Age* + *infs*:*dnmr* | 26 | (Intercept) | -16.053 | 1.600 | 1.06e-23 |
|  |  | *Age* | 0.174 | 0.025 | 2.88e-12 |
|  |  | *Age*:*dnmr* | -0.005 | 0.004 | 2.19e-01 |
|  |  | *dnmr*:*infs* | 1.688 | 0.370 | 4.99e-06 |
| *risk* ~ 1 + *Age* + *dnmr* + *infs*:*dnmr* | 27 | (Intercept) | -16.028 | 1.600 | 1.24e-23 |
|  |  | *Age* | 0.173 | 0.025 | 3.22e-12 |
|  |  | *dnmr* | -0.312 | 0.264 | 2.37e-01 |
|  |  | *dnmr*:*infs* | 1.674 | 0.369 | 5.58e-06 |
| *risk* ~ 1 + *Age* + *dnmr* + *dnmr*:*Age* + *infs*:*dnmr* | 28 | (Intercept) | -16.631 | 1.667 | 1.98e-23 |
|  |  | *Age* | 0.183 | 0.026 | 1.80e-12 |
|  |  | *dnmr* | 7.428 | 5.946 | 2.12e-01 |
|  |  | *Age*:*dnmr* | -0.121 | 0.093 | 1.94e-01 |
|  |  | *dnmr*:*infs* | 1.681 | 0.368 | 5.01e-06 |
| *risk* ~ 1 + *dnmr* + *infs* + *infs*:*Age* + *infs*:*dnmr* | 30 | (Intercept) | -5.103 | 0.061 | 0.00e+00 |
|  |  | *dnmr* | -0.159 | 0.266 | 5.50e-01 |
|  |  | *infs* | -6.561 | 2.813 | 1.97e-02 |
|  |  | *infs*:*Age* | 0.114 | 0.044 | 9.33e-03 |
|  |  | *dnmr*:*infs* | 0.961 | 0.386 | 1.29e-02 |
| *risk* ~ 1 + *infs* + *dnmr*:*Age* + *infs*:*Age* + *infs*:*dnmr* | 31 | (Intercept) | -5.103 | 0.061 | 0.00e+00 |
|  |  | *infs* | -6.581 | 2.814 | 1.93e-02 |
|  |  | *dnmr*:*Age* | -0.002 | 0.004 | 5.50e-01 |
|  |  | *infs*:*Age* | 0.114 | 0.044 | 9.14e-03 |
|  |  | *infs*:*dnmr* | 0.961 | 0.388 | 1.31e-02 |
| *risk* ~ 1 + *dnmr* + *infs* + *dnmr*:*Age* + *infs*:*Age* + *infs*:*dnmr* | 33 | (Intercept) | -5.103 | 0.061 | 0.00e+00 |
|  |  | *dnmr* | -0.230 | 5.937 | 9.69e-01 |
|  |  | *infs* | -6.552 | 2.914 | 2.46e-02 |
|  |  | *dnmr*:*Age* | 0.001 | 0.093 | 9.90e-01 |
|  |  | *infs*:*Age* | 0.114 | 0.045 | 1.22e-02 |
|  |  | *dnmr*:*infs* | 0.960 | 0.389 | 1.36e-02 |
| *risk* ~ 1 + *dnmr* + *infs*:*Age* + *infs*:*dnmr* | 36 | (Intercept) | -5.106 | 0.062 | 0.00e+00 |
|  |  | *dnmr* | -0.156 | 0.266 | 5.58e-01 |
|  |  | *infs*:*Age* | 0.011 | 0.002 | 3.55e-10 |
|  |  | *dnmr*:*infs* | 0.953 | 0.386 | 1.36e-02 |
| *risk* ~ 1 + *dnmr*:*Age* + *infs*:*Age* + *infs*:*dnmr* | 37 | (Intercept) | -5.107 | 0.062 | 0.00e+00 |
|  |  | *dnmr*:*Age* | -0.002 | 0.004 | 5.76e-01 |
|  |  | *Age*:*infs* | 0.011 | 0.002 | 3.46e-10 |
|  |  | *dnmr*:*infs* | 0.945 | 0.385 | 1.42e-02 |
| *risk* ~ 1 + *infs* + *dnmr*:*Age* + *infs*:*Age* | 38 | (Intercept) | -5.127 | 0.062 | 0.00e+00 |
|  |  | *infs* | -6.493 | 2.813 | 2.10e-02 |
|  |  | *dnmr*:*Age* | 0.003 | 0.003 | 2.71e-01 |
|  |  | *infs*:*Age* | 0.114 | 0.044 | 9.21e-03 |
| *risk* ~ 1 + *dnmr* + *infs* + *infs*:*Age* | 39 | (Intercept) | -5.127 | 0.062 | 0.00e+00 |
|  |  | *dnmr* | 0.208 | 0.191 | 2.76e-01 |
|  |  | *infs* | -6.509 | 2.813 | 2.07e-02 |
|  |  | *infs*:*Age* | 0.115 | 0.044 | 9.05e-03 |
| *risk* ~ 1 + *dnmr* + *infs* + *infs*:*dnmr* | 40 | (Intercept) | -5.103 | 0.061 | 0.00e+00 |
|  |  | *dnmr* | -0.159 | 0.266 | 5.50e-01 |
|  |  | *infs* | 0.718 | 0.117 | 7.67e-10 |
|  |  | *dnmr*:*infs* | 0.965 | 0.386 | 1.25e-02 |
| *risk* ~ 1 + *infs* + *dnmr*:*Age* + *infs*:*dnmr* | 41 | (Intercept) | -5.104 | 0.062 | 0.00e+00 |
|  |  | *infs* | 0.718 | 0.117 | 7.49e-10 |
|  |  | *dnmr*:*Age* | -0.002 | 0.004 | 5.70e-01 |
|  |  | *infs*:*dnmr* | 0.956 | 0.385 | 1.31e-02 |
| *risk* ~ 1 + *dnmr* + *dnmr*:*Age* + *infs*:*Age* + *infs*:*dnmr* | 42 | (Intercept) | -5.106 | 0.062 | 0.00e+00 |
|  |  | *dnmr* | -3.709 | 5.709 | 5.16e-01 |
|  |  | *dnmr*:*Age* | 0.056 | 0.089 | 5.33e-01 |
|  |  | *Age*:*infs* | 0.011 | 0.002 | 3.74e-10 |
|  |  | *dnmr*:*infs* | 0.950 | 0.386 | 1.40e-02 |
| *risk* ~ 1 + *dnmr* + *infs* + *dnmr*:*Age* + *infs*:*Age* | 44 | (Intercept) | -5.126 | 0.062 | 0.00e+00 |
|  |  | *dnmr* | -1.522 | 5.846 | 7.95e-01 |
|  |  | *infs* | -6.376 | 2.849 | 2.52e-02 |
|  |  | *dnmr*:*Age* | 0.027 | 0.092 | 7.67e-01 |
|  |  | *infs*:*Age* | 0.112 | 0.044 | 1.14e-02 |
| *risk* ~ 1 + *dnmr* + *infs* + *dnmr*:*Age* + *infs*:*dnmr* | 45 | (Intercept) | -5.103 | 0.061 | 0.00e+00 |
|  |  | *dnmr* | -4.100 | 5.708 | 4.73e-01 |
|  |  | *infs* | 0.718 | 0.117 | 7.67e-10 |
|  |  | *dnmr*:*Age* | 0.062 | 0.089 | 4.89e-01 |
|  |  | *dnmr*:*infs* | 0.963 | 0.386 | 1.27e-02 |
| *risk* ~ 1 + *Age* + *dnmr* | 46 | (Intercept) | -16.067 | 1.602 | 1.10e-23 |
|  |  | *Age* | 0.174 | 0.025 | 2.87e-12 |
|  |  | *dnmr* | 0.209 | 0.191 | 2.73e-01 |
| *risk* ~ 1 + *Age* + *dnmr*:*Age* | 48 | (Intercept) | -16.052 | 1.601 | 1.21e-23 |
|  |  | *Age* | 0.174 | 0.025 | 3.07e-12 |
|  |  | *Age*:*dnmr* | 0.003 | 0.003 | 2.92e-01 |
| *risk* ~ 1 + *Age* + *dnmr* + *dnmr*:*Age* | 49 | (Intercept) | -16.631 | 1.667 | 1.98e-23 |
|  |  | *Age* | 0.183 | 0.026 | 1.80e-12 |
|  |  | *dnmr* | 7.692 | 6.058 | 2.04e-01 |
|  |  | *Age*:*dnmr* | -0.117 | 0.095 | 2.18e-01 |
| *risk* ~ 1 + *dnmr*:*Age* + *infs*:*Age* | 50 | (Intercept) | -5.130 | 0.062 | 0.00e+00 |
|  |  | *dnmr*:*Age* | 0.003 | 0.003 | 2.63e-01 |
|  |  | *Age*:*infs* | 0.013 | 0.002 | 1.76e-13 |
| *risk* ~ 1 + *dnmr* + *infs*:*Age* | 51 | (Intercept) | -5.130 | 0.062 | 0.00e+00 |
|  |  | *dnmr* | 0.209 | 0.191 | 2.73e-01 |
|  |  | *infs*:*Age* | 0.013 | 0.002 | 1.76e-13 |
| *risk* ~ 1 + *infs* + *dnmr*:*Age* | 53 | (Intercept) | -5.127 | 0.062 | 0.00e+00 |
|  |  | *infs* | 0.800 | 0.110 | 4.15e-13 |
|  |  | *dnmr*:*Age* | 0.003 | 0.003 | 2.62e-01 |
| *risk* ~ 1 + *dnmr* + *infs* | 54 | (Intercept) | -5.127 | 0.062 | 0.00e+00 |
|  |  | *dnmr* | 0.209 | 0.191 | 2.72e-01 |
|  |  | *infs* | 0.800 | 0.110 | 4.15e-13 |
| *risk* ~ 1 + *dnmr* + *dnmr*:*Age* + *infs*:*Age* | 55 | (Intercept) | -5.129 | 0.062 | 0.00e+00 |
|  |  | *dnmr* | -3.609 | 5.771 | 5.32e-01 |
|  |  | *dnmr*:*Age* | 0.060 | 0.090 | 5.07e-01 |
|  |  | *Age*:*infs* | 0.013 | 0.002 | 1.85e-13 |
| *risk* ~ 1 + *dnmr* + *infs* + *dnmr*:*Age* | 56 | (Intercept) | -5.127 | 0.062 | 0.00e+00 |
|  |  | *dnmr* | -3.876 | 5.770 | 5.02e-01 |
|  |  | *infs* | 0.800 | 0.110 | 4.20e-13 |
|  |  | *dnmr*:*Age* | 0.064 | 0.090 | 4.78e-01 |
| *risk* ~ 1 + *dnmr* + *infs*:*dnmr* | 58 | (Intercept) | -4.949 | 0.052 | 0.00e+00 |
|  |  | *dnmr* | -0.313 | 0.264 | 2.35e-01 |
|  |  | *dnmr*:*infs* | 1.683 | 0.368 | 4.84e-06 |
| *risk* ~ 1 + *dnmr*:*Age* + *infs*:*dnmr* | 59 | (Intercept) | -4.949 | 0.052 | 0.00e+00 |
|  |  | *dnmr*:*Age* | -0.005 | 0.004 | 2.46e-01 |
|  |  | *dnmr*:*infs* | 1.674 | 0.367 | 5.12e-06 |
| *risk* ~ 1 + *dnmr* + *dnmr*:*Age* + *infs*:*dnmr* | 60 | (Intercept) | -4.949 | 0.052 | 0.00e+00 |
|  |  | *dnmr* | -4.254 | 5.706 | 4.56e-01 |
|  |  | *dnmr*:*Age* | 0.062 | 0.089 | 4.88e-01 |
|  |  | *dnmr*:*infs* | 1.681 | 0.368 | 4.97e-06 |
| *risk* ~ 1 + *dnmr*:*Age* | 62 | (Intercept) | -4.949 | 0.052 | 0.00e+00 |
|  |  | *dnmr*:*Age* | 0.003 | 0.003 | 2.62e-01 |
| *risk* ~ 1 + *dnmr* | 63 | (Intercept) | -4.949 | 0.052 | 0.00e+00 |
|  |  | *dnmr* | 0.209 | 0.191 | 2.73e-01 |
| *risk* ~ 1 + *dnmr* + *dnmr*:*Age* | 64 | (Intercept) | -4.949 | 0.052 | 0.00e+00 |
|  |  | *dnmr* | -3.990 | 5.824 | 4.93e-01 |
|  |  | *dnmr*:*Age* | 0.066 | 0.091 | 4.70e-01 |

**Note:** here, we presented the regressions coefficients obtained from the regression analysis for the subset (43 models with Signf=0; Supplementary Table 11) of all possible logistic regression (64 models; Supplementary Table 11) for females/males, aged 60-75 years, *APOE4* non-carriers. Response variable *risk* is risk of AD+. Independent variables* *dnmr*=1 (*dnmr*<50), *dnmr*=0 (*dnmr*>=50), *infs*=1 (for subjects with prior infection history during January 1, 2006 and January 1, 2016), *infs*=0 (for subjects without prior infection history during January 1, 2006 and January 1, 2016), and *age* at the baseline date January 1, 2006 as the *Age* variable. In Supplementary Table 11, a logistic regression set, having 64 models with linear terms and their pairwise interactions and corresponding to females/ males *age*d 60-75 were analyzed and presented in ascending order by AIC value. Signf=1 means that all regression coefficient were significant (P-value<0.05) in a specific model, Signf=0 means the opposite. Here and in the Supplementary Table 11, for regression model a short notation for logistic regression used. For instance, *risk* ~ 1 + *Age* + *dnmr* + *infs* + *dnmr***Age* + *infs***dnmr* denotes a standard logistic regression equation ln(*risk*/(1-*risk*)) = Intercept + b_1_**Age* + b_2_**dnmr* + b_3_**infs* + b_12_**Age***dnmr* + b_31_**infs***dnmr* where ln(x) natural logarithm, Intercept is a constant called the bias term (or intercept term), b_1_, b_2_, b_3_, b_12_, b_31_ are the regression coefficients corresponding to the *Age*, *dnmr*, *infs*, *Age***dnmr*, *infs***dnmr* terms in the regression model. The 'Model’ column shows the models presented in the short notation. The columns ‘Num’ gives the number of the model in the list arranged from smallest to largest AIC value (Supplementary Table 11). The numbers in the ‘Estimate‘ column correspond to the coefficients for the regression terms shown in the 'Model/Term’ column. The ‘Std.err’ and ‘P-Value’ presents respectively the standard error and the P-value. For all terms containing variable *Age* a unit of their measurement is 1/year; all other terms are the dimensionless quantities. Scientific notation ‘e’ means that the base number is multiplied by 10 raised to the given power.
