# Supplementary material for: Traffic-related air pollution significantly aggravates the detrimental effect of infections on the risk of Alzheimer’s disease and other dementias, especially in non-carriers of APOE4
Source: Front Dement. 2026 Jan 12;4:1668381. doi: 10.3389/frdem.2025.1668381 (PMC12833968; doi:10.3389/frdem.2025.1668381)
Supplement: Supplementary file 3 [file Supplementary_file_3.docx]

Supplementary Material 3

# Bias correction for handling rare events in logistic regression

Estimation and inference from generalized linear models use implicit and explicit bias reduction

methods (Kosmidis, 2014), and other penalized maximum likelihood methods. Currently supported

methods include the mean bias-reducing adjusted scores approach in (Firth, 1993) and (Kosmidis

& Firth, 2009), the median bias-reduction adjusted scores approach in (Kenne Pagui et al., 2017),

the correction of the asymptotic bias in (Cordeiro & McCullagh, 1991), the mixed bias-reduction

adjusted scores approach in (Kosmidis et al., 2020), maximum penalized likelihood with powers of

the Jeffreys prior as penalty, and maximum likelihood.

**1.1 The brglm2 R package**

The brglm2 R package is highly effective for handling rare events in logistic regression because it provides methods that produce finite and less biased estimates even in cases of complete or quasi-complete separation, where traditional maximum likelihood (ML) estimation fails or produces infinite estimates.

The options for score adjustment type are methods for fitting generalized linear models and include "AS_median" for median bias reduction, "AS_mean" for mean bias reduction, "AS_mixed" for mixed bias reduction, and others like "MPL_Jeffreys", "ML" (maximum likelihood), and "correction" (asymptotic bias correction). These adjustments improve statistical properties and provide finite estimates, particularly in cases where maximum likelihood estimates can be infinite, such as with complete or quasi-complete separation in logistic regression.

- "AS_median": Median bias reduction, which requests the median-bias reducing adjusted scores.
- "AS_mean": Mean bias reduction, which requests the mean-bias reducing adjusted scores.
- "AS_mixed": Mixed bias reduction, which results in mean bias reduction for regression parameters and median bias reduction for the dispersion parameter (if any).
- "MPL_Jeffreys": Maximum penalized likelihood with Jeffreys prior, a method using a penalized likelihood with powers of the Jeffreys prior as a penalty.
- "ML": Maximum likelihood, the standard estimation method.
- "correction": Asymptotic bias correction, another type of bias correction.

**NOTE**

"AS_mean" implements the mean bias-reducing adjusted scores approach, which is Firth's original method. "MPL_Jeffreys" implements maximum penalized likelihood with the Jeffreys prior as a penalty, which is equivalent to the "AS_mean" approach for binomial GLMs (logistic regression). By using one of these type specifications in brglm2 R package, one ensures that the model is fit using Firth's method.

**1.2 Results**

The results of the bias correction of the models in Table 3 are presented in the tables: Supplementary Table 3.1 - Supplementary Table 3.5.

Supplementary Table 3.1 Regression coefficients for the best model among all models considered in this study (with score adjustment type "AS_mean")

| Model/Term | Estimate | Std.Error | P-Value |
| --- | --- | --- | --- |
| **model, females/males, 60-75** |  |  |  |
| (Intercept) | -15.486 | 0.982 | 5.14E-56 |
| *Age* | 0.171 (1/year) | 0.015 | 5.89E-29 |
| infs | 0.430 | 0.077 | 1.94E-08 |
| *infs***dnmr* | 0.575 | 0.209 | 5.89E-03 |
| **The above model with the relevant covariates** |  |  |  |
| (Intercept) | -15.230 | 1.027 | 1.02E-49 |
| *Age* | 0.165 (1/year) | 0.016 | 2.97E-25 |
| *infs* | 0.439 | 0.079 | 2.75E-08 |
| *education* | -0.155 | 0.081 | 5.64E-02 |
| *smoking* | 0.017 | 0.068 | 8.07E-01 |
| *infs*dnmr* | 0.602 | 0.209 | 4.02E-03 |
| *tsi1* | -0.031 | 0.108 | 7.75E-01 |
| *tsi2* | 0.029 | 0.106 | 7.84E-01 |
| *tsi3* | 0.040 | 0.106 | 7.03E-01 |
| *tsi4* | 0.327 | 0.099 | 9.74E-04 |

**Note:** here, the regression coefficients for the two models presented in Table3, one without the covariates and anther with the covatiates, were corrected using the score adjustment type "AS_mean".

Supplementary Table 3.2 Regression coefficients after correction with score adjustment type "AS_median".

| Model/Term | Estimate | Std.Error | P-Value |
| --- | --- | --- | --- |
| **model, females/males, 60-75** |  |  |  |
| (Intercept) | -15.488 | 0.983 | 5.98E-56 |
| *Age* | 0.171 (1/year) | 0.015 | 6.38E-29 |
| infs | 0.429 | 0.077 | 2.17E-08 |
| *infs***dnmr* | 0.564 | 0.210 | 7.20E-03 |
| **The above model with the relevant covariates** |  |  |  |
| (Intercept) | -15.231 | 1.028 | 1.15E-49 |
| *Age* | 0.165 (1/year) | 0.016 | 3.16E-25 |
| *infs* | 0.438 | 0.079 | 3.05E-08 |
| *infs*dnmr* | 0.591 | 0.210 | 4.94E-03 |
| *education* | -0.156 | 0.081 | 5.46E-02 |
| *smoking* | 0.017 | 0.068 | 8.02E-01 |
| *tsi1* | -0.031 | 0.108 | 7.75E-01 |
| *tsi2* | 0.029 | 0.106 | 7.84E-01 |
| *tsi3* | 0.040 | 0.106 | 7.02E-01 |
| *tsi4* | 0.328 | 0.099 | 9.61E-04 |

**Note:** here, the regression coefficients for the two models presented in Table3, one without the covariates and anther with the covatiates, were corrected using the score adjustment type "AS_median".

Supplementary Table 3.3 Regression coefficients after correction with score adjustment type "AS_mixed".

| Model/Term | Estimate | Std.Error | P-Value |
| --- | --- | --- | --- |
| **model, females/males, 60-75** |  |  |  |
| (Intercept) | -15.486 | 0.982 | 5.14E-56 |
| *Age* | 0.171 (1/year) | 0.015 | 5.89E-29 |
| infs | 0.430 | 0.077 | 1.94E-08 |
| *infs***dnmr* | 0.575 | 0.209 | 5.89E-03 |
| **The above model with the relevant covariates** |  |  |  |
| (Intercept) | -15.230 | 1.027 | 1.02E-49 |
| *Age* | 0.165 (1/year) | 0.016 | 2.97E-25 |
| *infs* | 0.439 | 0.079 | 2.75E-08 |
| *infs*dnmr* | 0.602 | 0.209 | 4.02E-03 |
| *education* | -0.155 | 0.081 | 5.64E-02 |
| *smoking* | 0.017 | 0.068 | 8.07E-01 |
| *tsi1* | -0.031 | 0.108 | 7.75E-01 |
| *tsi2* | 0.029 | 0.106 | 7.84E-01 |
| *tsi3* | 0.040 | 0.106 | 7.03E-01 |
| *tsi4* | 0.327 | 0.099 | 9.74E-04 |

**Note:** here, the regression coefficients for the two models presented in Table3, one without the covariates and anther with the covatiates, were corrected using the score adjustment type "AS_mixed".

Supplementary Table 3.4 Regression coefficients after correction with score adjustment type "correction".

| Model/Term | Estimate | Std.Error | P-Value |
| --- | --- | --- | --- |
| **model, females/males, 60-75** |  |  |  |
| (Intercept) | -15.486 | 0.982 | 5.14E-56 |
| *Age* | 0.171 (1/year) | 0.015 | 5.89E-29 |
| infs | 0.430 | 0.077 | 1.94E-08 |
| *infs***dnmr* | 0.575 | 0.209 | 5.87E-03 |
| **The above model with the relevant covariates** |  |  |  |
| (Intercept) | -15.230 | 1.027 | 1.02E-49 |
| *Age* | 0.165 (1/year) | 0.016 | 2.97E-25 |
| *infs* | 0.439 | 0.079 | 2.75E-08 |
| *infs*dnmr* | 0.602 | 0.209 | 4.00E-03 |
| *education* | -0.155 | 0.081 | 5.64E-02 |
| *smoking* | 0.017 | 0.068 | 8.07E-01 |
| *tsi1* | -0.031 | 0.108 | 7.75E-01 |
| *tsi2* | 0.029 | 0.106 | 7.84E-01 |
| *tsi3* | 0.040 | 0.106 | 7.03E-01 |
| *tsi4* | 0.327 | 0.099 | 9.73E-04 |

**Note:** here, the regression coefficients for the two models presented in Table3, one without the covariates and anther with the covatiates, were corrected using the score adjustment type "correction".

Supplementary Table 3.5 Regression coefficients after correction with score adjustment type"MPL_Jeffreys".

| Model/Term | Estimate | Std.Error | P-Value |
| --- | --- | --- | --- |
| **model, females/males, 60-75** |  |  |  |
| (Intercept) | -15.486 | 0.982 | 5.14E-56 |
| *Age* | 0.171 (1/year) | 0.015 | 5.89E-29 |
| infs | 0.430 | 0.077 | 1.94E-08 |
| *infs***dnmr* | 0.575 | 0.209 | 5.89E-03 |
| **The above model with the relevant covariates** |  |  |  |
| (Intercept) | -15.230 | 1.027 | 1.02E-49 |
| *Age* | 0.165 | 0.016 | 2.97E-25 |
| *infs* | 0.439 | 0.079 | 2.75E-08 |
| *infs*dnmr* | 0.602 | 0.209 | 4.02E-03 |
| *education* | -0.155 | 0.081 | 5.64E-02 |
| *smoking* | 0.017 (1/year) | 0.068 | 8.07E-01 |
| *tsi1* | -0.031 | 0.108 | 7.75E-01 |
| *tsi2* | 0.029 | 0.106 | 7.84E-01 |
| *tsi3* | 0.040 | 0.106 | 7.03E-01 |
| *tsi4* | 0.327 | 0.099 | 9.74E-04 |

**Note:** here, the regression coefficients for the two models presented in Table3, one without the covariates and anther with the covatiates, were corrected using the score adjustment type "MPL_Jeffreys".

The results of the analysis showed that the coefficients for the logistic regression changed about 5% when applying the bias correction methods. It supported sustainbility of our logistic regression model.

1. **Estimate logistic regression coefficients using SMOTE**

Synthetic Minority Over-sampling Technique (SMOTE) is a oversampling technique which synthesizes a new minority instance between a pair of one minority instance and one of its K nearest neighbor (Chawla et al., 2002).

Several R packages implement SMOTE for handling imbalanced datasets in classification problems. The smotefamily package (Class Imbalance Problem Based on SMOTE, 2025) offers a collection of oversampling techniques, including the standard SMOTE algorithm. It is designed to create synthetic minority instances to balance class distributions.

To estimate logistic regression coefficients multiple times using SMOTE in R, we employed a resampling approach, where SMOTE is applied to repeatedly oversample to balance the data and fit the model, storing the coefficients from each iteration.

**2.1 Rare events in logistic regression**

Balancing imbalanced datasets for logistic regression using SMOTE from the smotefamily package in R, while aiming to preserve the original probability of the rare event, involves careful application of SMOTE and potential post-processing. The smotefamily package generates synthetic data for the minority class, increasing its representation. However, directly applying SMOTE might alter the overall proportion of the rare event in the entire dataset, which could affect the estimated probabilities if not handled appropriately. To maintain the original probability of the rare event, we applied a bias adjustment to the intercept in our analysis.

**2.2 Results**

The SMOTE was applied with various parameter K, which refers to the number of nearest neighbors used to generate new synthetic samples for the minority class, and with dup_size=0, which is the setting that aims to balance the classes by generating enough synthetic minority instances to match the number of majority instances. The number of iterations was equal to 100.

The results are presented in Supplementary Tables 3.6-3.9 below. For example, for the model (without covariates) in Supplementary 3.6, the means and standard deviation of the coefficients over all iterations of SMOTE are as follows

(Intercept) : Mean = -15.456442, Sd = 0.001677

*Age*  : Mean = 0.170003, Sd = 0.000026

*infs*  : Mean = 0.459403, Sd = 0.000382

infs*dnmr : Mean = 0.486897, Sd = 0.004114

Each Supplementary Table 3.6-3.9 presents the result of applying SMOTE with the parameter K=5, K=3, K=2, K=1 respectively.

Usually, SMOTE can be used to improve the performance and reliability (or sustainability in the sense of consistent performance) of models, including logistic regression, when dealing with imbalanced datasets.

Note that in our case our goal is to evaluate (applying SMOTE) how sensitive and sustainable our model and data were to relatively small changes in initial conditions. Looking at Supplementary Tables 3.6-3.9 one by one, one can notice that, in general, the coefficients get closer to the coefficients in Table 3. Such a behavior is due to the parameter K, which changes respectively as K=5, K=3, K=2, K=1 when going over Supplementary Tables 3.6-3.9. Basically, the smaller K, the “nearer” the dataset (that SMOTE generates) to the initial dataset (without “perturbations”) and the coefficients estimates are closer to the coefficients corresponding to the initial dataset.

Note that for the model without covariates and for SMOTE iterations with K=1 the coefficient for the term *infs***dnmr* equals to 0.553, which is very close to 0.558, the coefficient for the term *infs***dnmr* in Table 3. Though, for the model with covariates and for SMOTE iterations with K=1 the coefficient for the term *infs***dnmr* equals to 0.361, which is not close to 0.586, the coefficient for the term *infs***dnmr* in Table 3. The reason for that is that for the model without covariates SMOTE worked in the 3-dimensional features space while for the model with covariates SMOTE worked in the 9-dimensional features space. In the 9-dimensional features space, it is much hard to find nearby neighbors than in the 3-dimensional features space – this is so called *curse of dimensionality.*

Notwithstanding, our SMOTE analysis still showed an upward trend, meaning the coefficients, in general, consistently increased in performance. So, our model and data were sensitive and sustainable to relatively small changes in initial conditions. Thus, the results allowed us to successfully check the absence of potential tangible irregularities in the model and data.

Therefore, the SMOTE results supported a good level of sensitivity and sustainability in our analysis.

Supplementary Table 3.6 Means and standard deviations for the regression coefficients over 100 SMOTE iterations for K=5.

| Model/Term | Mean | Sd |
| --- | --- | --- |
| **model, females/males, 60-75** |  |  |
| (Intercept) | -15.456442 | 0.001677 |
| *Age* | 0.170003 (1/year) | 0.000026 |
| infs | 0.459403 | 0.000382 |
| *infs***dnmr* | 0.486897 | 0.004114 |
| **The above model with the relevant covariates** |  |  |
| (Intercept) | -15.460413 | 0.010622 |
| *Age* | 0.169158 (1/year) | 0.000159 |
| *infs* | 0.371578 | 0.002220 |
| *infs*dnmr* | 0.308861 | 0.010550 |
| *education* | -0.255678 | 0.002045 |
| *smoking* | 0.036795 | 0.001366 |
| *tsi1* | -0.053543 | 0.002125 |
| *tsi2* | 0.044099 | 0.002081 |
| *tsi3* | 0.027790 | 0.002019 |
| *tsi4* | 0.322641 | 0.002157 |

**Note:**  Column ‘Mean’ contains the means, column ‘Sd’ contains the standard deviations.

Supplementary Table 3.7 Means and standard deviations for the regression coefficients over 100 SMOTE iterations for K=3.

| Model/Term | Mean | Sd |
| --- | --- | --- |
| **model, females/males, 60-75** |  |  |
| (Intercept) | -15.459447 | 0.001064 |
| *Age* | 0.170047 (1/year) | 0.000017 |
| infs | 0.456872 | 0.000284 |
| *infs***dnmr* | 0.516163 | 0.002958 |
| **The above model with the relevant covariates** |  |  |
| (Intercept) | -15.529541 | 0.008187 |
| *Age* | 0.170060 (1/year) | 0.000125 |
| *infs* | 0.397814 | 0.001750 |
| *infs*dnmr* | 0.331961 | 0.009726 |
| *education* | -0.219714 | 0.001377 |
| *smoking* | 0.019530 | 0.000869 |
| *tsi1* | -0.041204 | 0.001467 |
| *tsi2* | 0.053282 | 0.001299 |
| *tsi3* | 0.046005 | 0.001206 |
| *tsi4* | 0.328448 | 0.001269 |

**Note:**  Column ‘Mean’ contains the means, column ‘Sd’ contains the standard deviations.

Supplementary Table 3.8 Means and standard deviations for the regression coefficients over 100 SMOTE iterations for K=2.

| Model/Term | Mean | Sd |
| --- | --- | --- |
| **model, females/males, 60-75** |  |  |
| (Intercept) | -15.456260 | 0.000785 |
| *Age* | 0.169996 (1/year) | 0.000012 |
| infs | 0.456047 | 0.000256 |
| *infs***dnmr* | 0.525988 | 0.002635 |
| **The above model with the relevant covariates** |  |  |
| (Intercept) | -15.504432 | 0.006518 |
| *Age* | 0.169431 (1/year) | 0.000099 |
| *infs* | 0.415664 | 0.001286 |
| *infs*dnmr* | 0.364446 | 0.007804 |
| *education* | -0.189363 | 0.000791 |
| *smoking* | 0.018321 | 0.000818 |
| *tsi1* | -0.034346 | 0.001128 |
| *tsi2* | 0.059123 | 0.000915 |
| *tsi3* | 0.053788 | 0.000802 |
| *tsi4* | 0.333389 | 0.001126 |

**Note:**  Column ‘Mean’ contains the means, column ‘Sd’ contains the standard deviations.

Supplementary Table 3.9 Means and standard deviations for the regression coefficients over 100 SMOTE iterations for K=1.

| Model/Term | Mean | Sd |
| --- | --- | --- |
| **model, females/males, 60-75** |  |  |
| (Intercept) | -15.465673 | 0.000318 |
| *Age* | 0.170143 (1/year) | 0.000005 |
| infs | 0.453092 | 0.000007 |
| *infs***dnmr* | 0.553403 | 0.000128 |
| **The above model with the relevant covariates** |  |  |
| (Intercept) | -15.554280 | 0.004178 |
| *Age* | 0.170022 | 0.000065 |
| *infs* | 0.436240 | 0.000798 |
| *infs*dnmr* | 0.361180 | 0.005356 |
| *education* | -0.168883 | 0.000428 |
| *smoking* | 0.016903 | 0.000372 |
| *tsi1* | -0.030026 | 0.000440 |
| *tsi2* | 0.059552 | 0.000441 |
| *tsi3* | 0.060090 | 0.000387 |
| *tsi4* | 0.341890 | 0.000627 |

**Note:**  Column ‘Mean’ contains the means, column ‘Sd’ contains the standard deviations.

References

Bias Reduction in Generalized Linear Models (2025). *R package brglm2*. Available online at: https://cran.r-project.org/web/packages/brglm2/brglm2.pdf (Accessed November 6, 2025).

Chawla, N. V., Bowyer, K. W., Hall, L. O., and Kegelmeyer, W. P. (2002). Smote: Synthetic minority over-sampling technique. *Journal of Artificial Intelligence Research*, 16, 321-357. https://doi.org/: https://doi.org/10.1613/jair.953

Class Imbalance Problem Based on SMOTE (2025). *R package smotefamily*. Available online at: https://cran.r-project.org/web/packages/smotefamily/smotefamily.pdf (Accessed November 6, 2025).

Cordeiro, G. M., McCullagh, P. (1991). Bias correction in generalized linear models. Journal of the Royal Statistical Society. Series B (Methodological), 53, 629-643.https://doi.org/:10.1111/j.2517-6161.1991.tb01852.x

Firth, D. (1993). Bias reduction of maximum likelihood estimates, Biometrika, 80, 27-38. https://doi.org/:10.2307/2336755

Kenne Pagui, E. C, Salvan, A., Sartori, N. (2017). Median bias reduction of maximum likelihood estimates. Biometrika, 104, 923–938. https://doi.org/:10.1093/biomet/asx046

Kosmidis, I. (2014). Bias in parametric estimation: reduction and useful side-effects. WIRE Computational Statistics, 6, 185-196. https://doi.org/:10.1002/wics.1296

Kosmidis, I., Firth, D. (2009). Bias reduction in exponential family nonlinear models. Biometrika, 96, 793-804. https://doi.org/:10.1093/biomet/asp055

Kosmidis, I., Kenne Pagui, E. C., Sartori, N. (2020). Mean and median bias reduction in generalized linear models. Statistics and Computing, 30, 43-59. https://doi.org/:10.1007/s11222-019-09860-6
