# Supplementary material for: Traffic-related air pollution significantly aggravates the detrimental effect of infections on the risk of Alzheimer’s disease and other dementias, especially in non-carriers of APOE4
Source: Front Dement. 2026 Jan 12;4:1668381. doi: 10.3389/frdem.2025.1668381 (PMC12833968; doi:10.3389/frdem.2025.1668381)
Supplement: Supplementary file 4 [file Supplementary_file_4.docx]

Supplementary Material

# **Visual inspection for checking linearity**

Visual inspection uses smoothed plots of log-odds. This method involves creating a plot of the log-odds of the outcome against the continuous predictor (in our case Age), with a smoother to visualize the relationship. Here, the LOESS (Locally Estimated Scatterplot Smoothing) smoother, which is a non-parametric statistical method, was used.

Supplementary Figure 4.1 Plot of Log-Odds of the AD risk by Age.


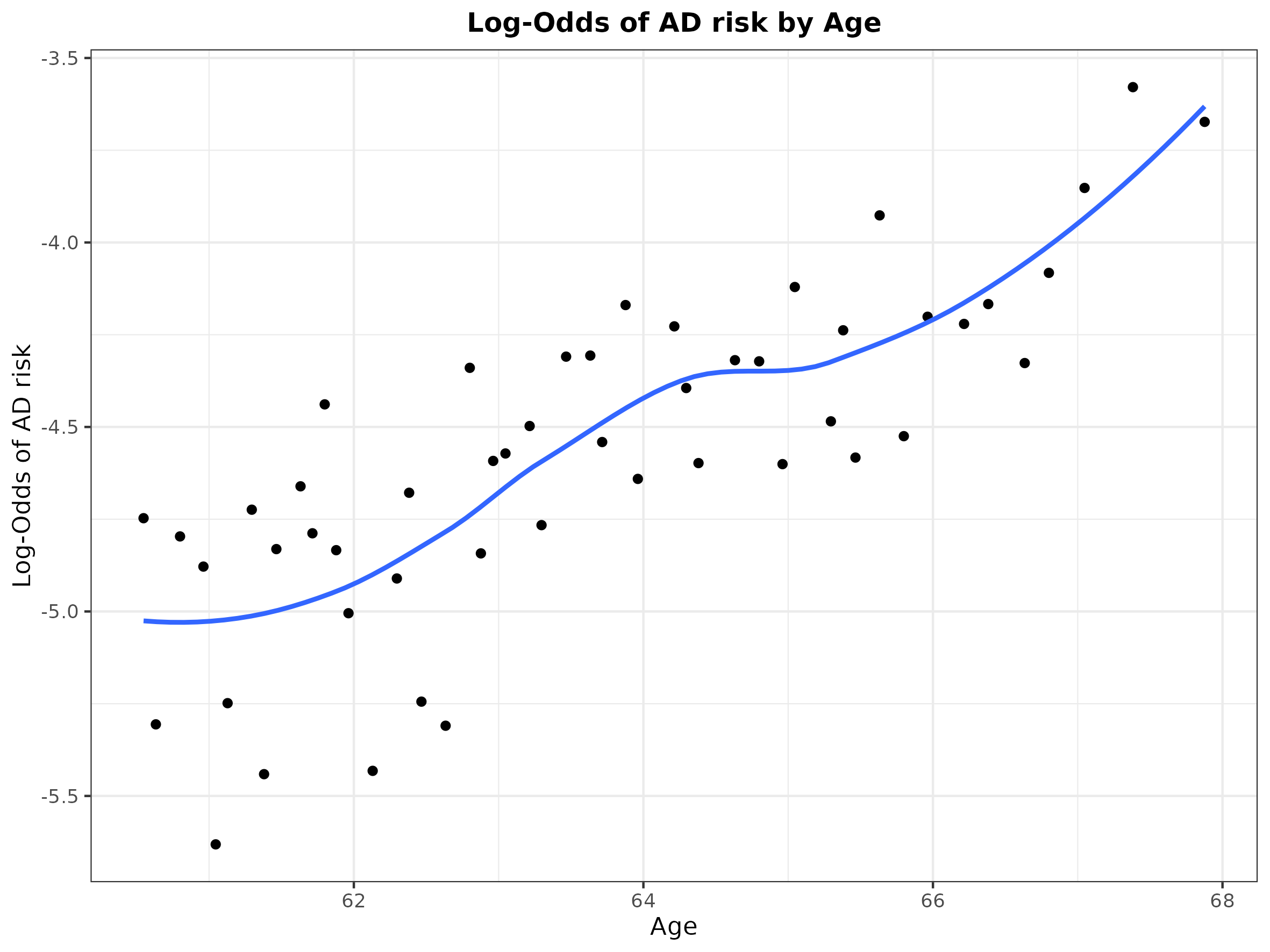


**Note:** the LOESS smoother is shown in blue color. This plot corresponds to the model for females and males aged 60-75 in Table 3. The model did not include covariates: *education, smoking, tsi1, tsi2, tsi3, and tsi4*

Supplementary Figure 4.2 Plot of Log-Odds of the AD risk by Age .


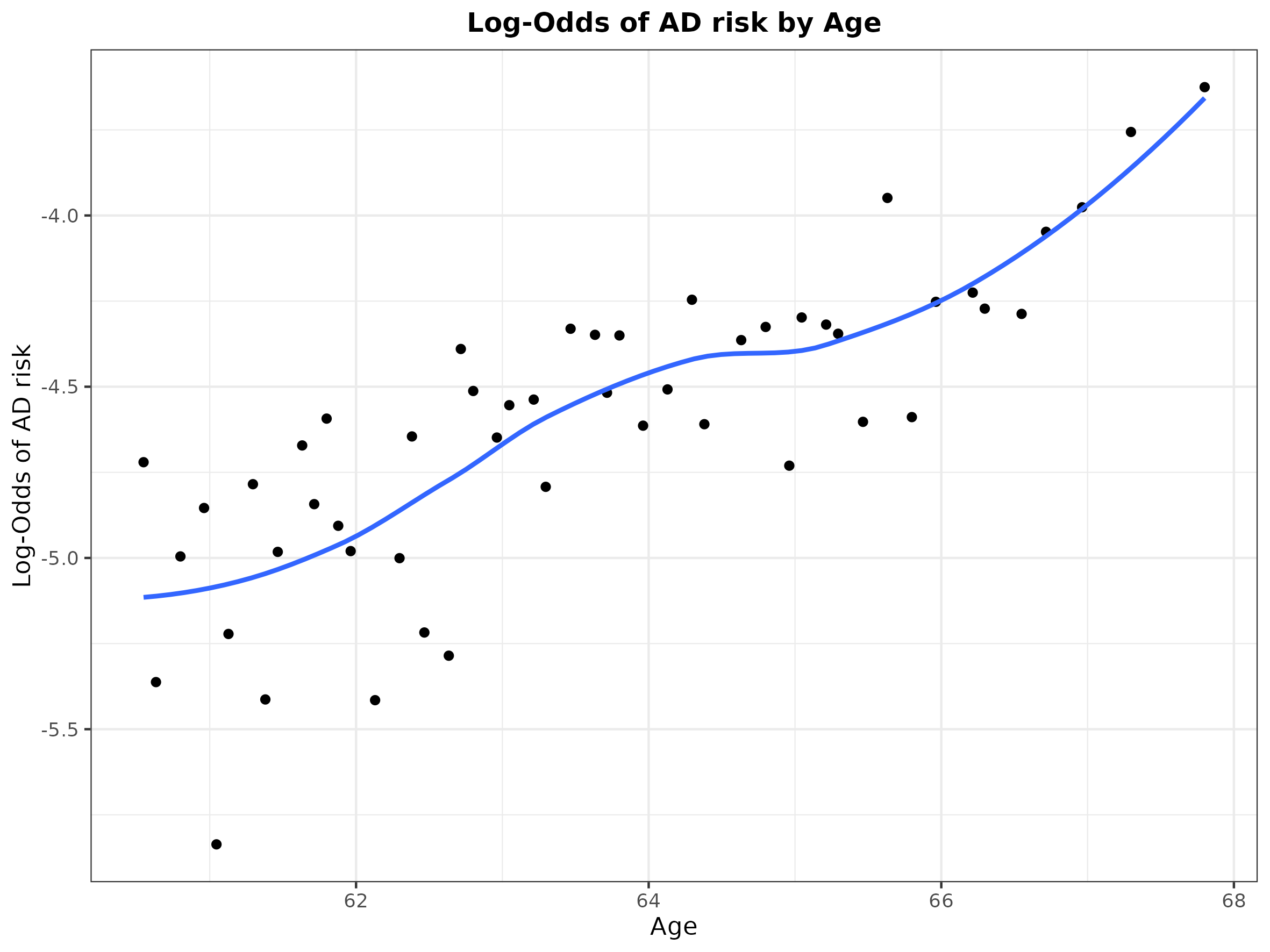


**Note:** the LOESS smoother is shown in blue color. This plot corresponds to the model for females and males aged 60-75 in Table 3. The model included covariates: *education, smoking, tsi1, tsi2, tsi3, and tsi4*

If the LOESS smoother (or a similar non-parametric smoother) shows a roughly linear trend, the assumption of linearity in the logit is likely met. Deviations from linearity suggest a non-linear relationship.

In our case, the plots support linearity of Log-Odds of the AD risk by Age.

Note that binary independent variables in a linear regression are inherently linear.

# **Marginal Model Plots**

Marginal Model Plots (MMPs) are a diagnostic tool used to assess the fit of a regression model, including logistic regression models. They compare the smoothed observed response against the smoothed fitted values for each predictor in the model. This helps in visually identifying potential issues such as nonlinearity and omitted variables. Here, the LOESS (Locally Estimated Scatterplot Smoothing) smoother, which is a non-parametric statistical method, was used.

The following two figures Supplementary Figure 4.1 and Supplementary Figure 4.2 correspond to the model for females and males aged 60-75. The model did not include covariates: *education, smoking, tsi1, tsi2, tsi3, and tsi4*

**Supplementary Figure 4.3** Marginal model plot for Age variable for the case without covariates.


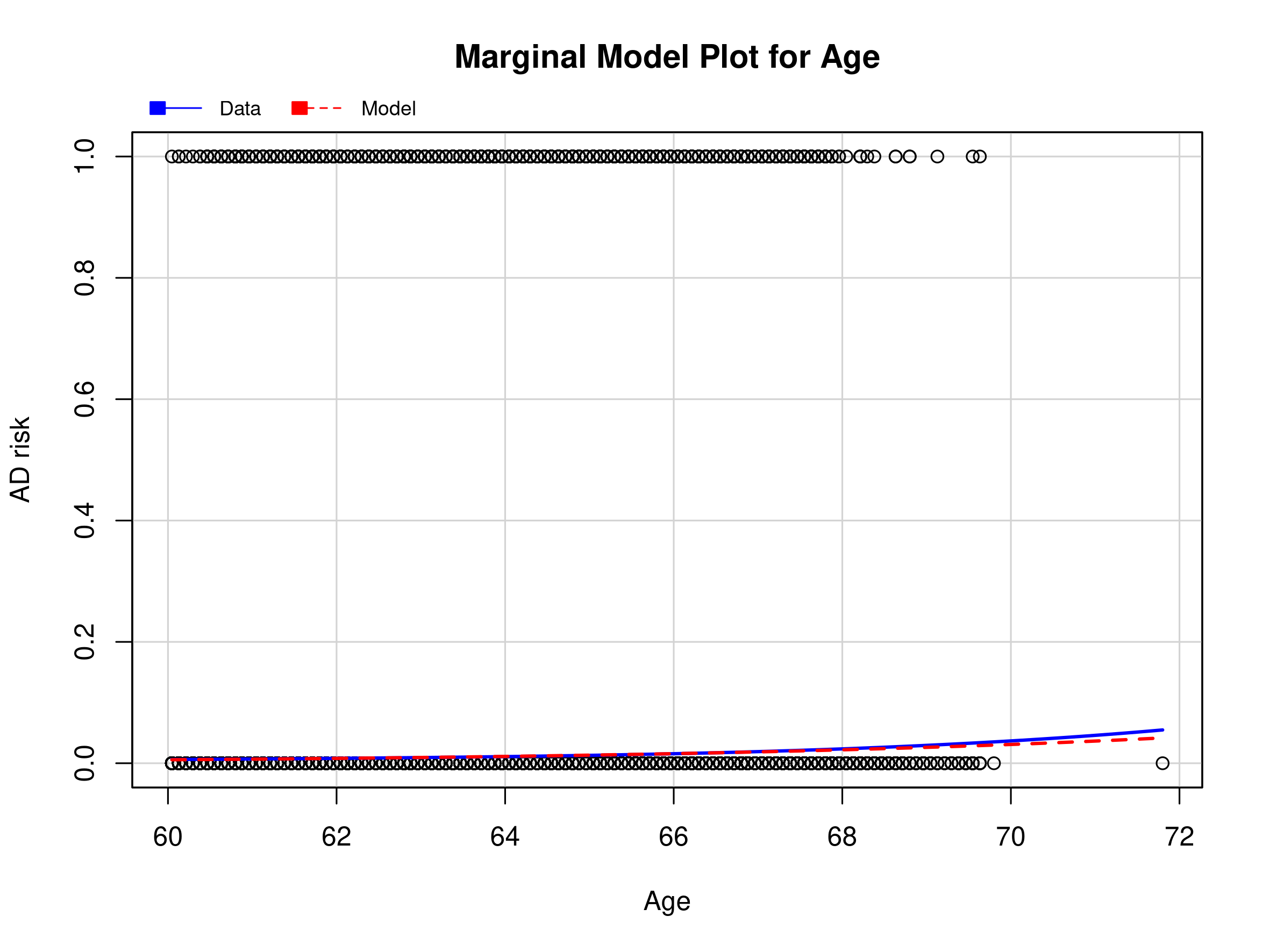
**Note:** this plot corresponds to the model for females and males aged 60-75 in Table 3. The model did not include covariates: *education, smoking, tsi1, tsi2, tsi3, and tsi4.* The points represent the observed values of the binary outcome (0 or 1) against the predictor variable. The blue smoothed line line represents the smoothed observed proportions of the outcome (e.g., the proportion of 1s) across the range of the predictor. The red smoothed line represents the smoothed fitted probabilities from the logistic regression model across the range of the predictor.

**Supplementary Figure 4.4** Marginal model plot for Age variable with covariates.

**
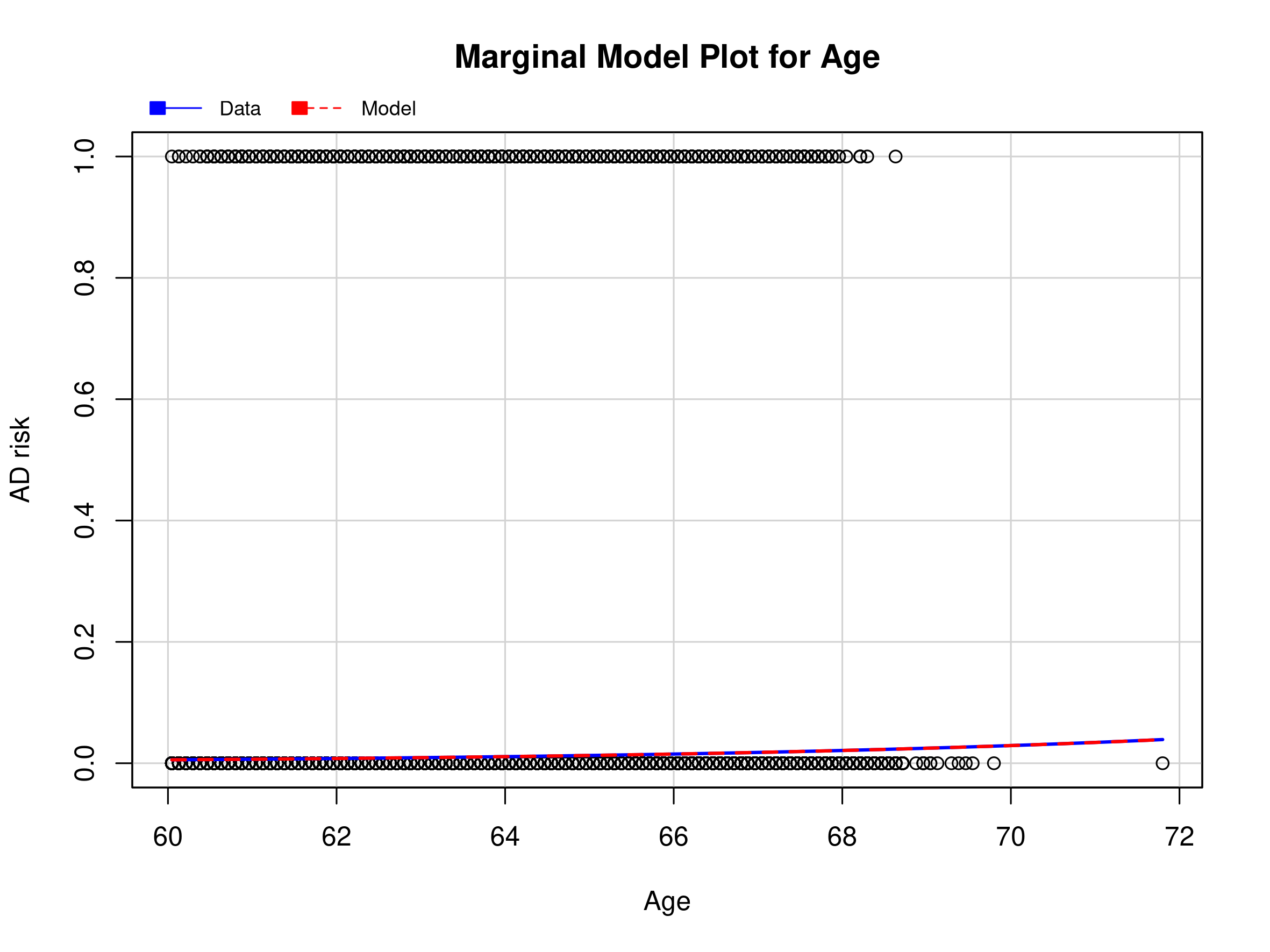
**

**Note:** this plot corresponds to the model for females and males aged 60-75 in Table 3. The model included covariates: *education, smoking, tsi1, tsi2, tsi3, and tsi4.* The points represent the observed values of the binary outcome (0 or 1) against the predictor variable. The blue smoothed line line represents the smoothed observed proportions of the outcome (e.g., the proportion of 1s) across the range of the predictor. The red smoothed line represents the smoothed fitted probabilities from the logistic regression model across the range of the predictor.

In our case, our logistic regression models are a good fit for the Age predictor being as linear because the blue (smoothed observed) and red (smoothed fitted) lines are closely aligned. So, the plot supports linearity of Log-Odds of the AD risk by Age.

# **Binned residual plot**

Binned residuals are created by grouping observations into bins based on their fitted values (e.g., predicted probabilities or counts). Within each bin, the average residual is calculated and plotted against the average fitted value. This approach creates a clearer visualization by smoothing out the noise caused by discrete responses.

For assessing the overall fit and linearity of the logistic model, the binned residuals plot is more reliable than attempting to check assumptions directly with raw residuals. The binned plot is a practical and effective diagnostic tool that adapts the concept of residual plots to the discrete, binary nature of logistic regression. It is the most reliable graphical tool for evaluating the overall fit of the logistic model itself, such as assessing the linearity assumption in the log-odds or detecting systematic patterns in the residuals.

In a binned residuals plot, the 95% confidence limits represent the expected range for the residuals if the model is a good fit. They are calculated as plus or minus two standard errors around the average residual for each bin, which is approximately (1.96*standarderror) for a 95% confidence level. If the model is correct, about 95% of the binned residuals should fall within these bounds.

In all plots below, the horizontal line at zero in a binned residual plot is reference line for a perfect prediction. It indicates where the predicted value from the model exactly matches the observed value (i.e., the residual, or error, is zero).

The following two figures Supplementary Figure 4.5 and Supplementary Figure 4.6 correspond to the model for females and males aged 60-75 in Table 3. The model did not include covariates: *education, smoking, tsi1, tsi2, tsi3, and tsi4*

**Supplementary Figure 4.5** Binned residuals plot for the model without covariates for the range for the expected probabilities xlim=0.004-0.010.


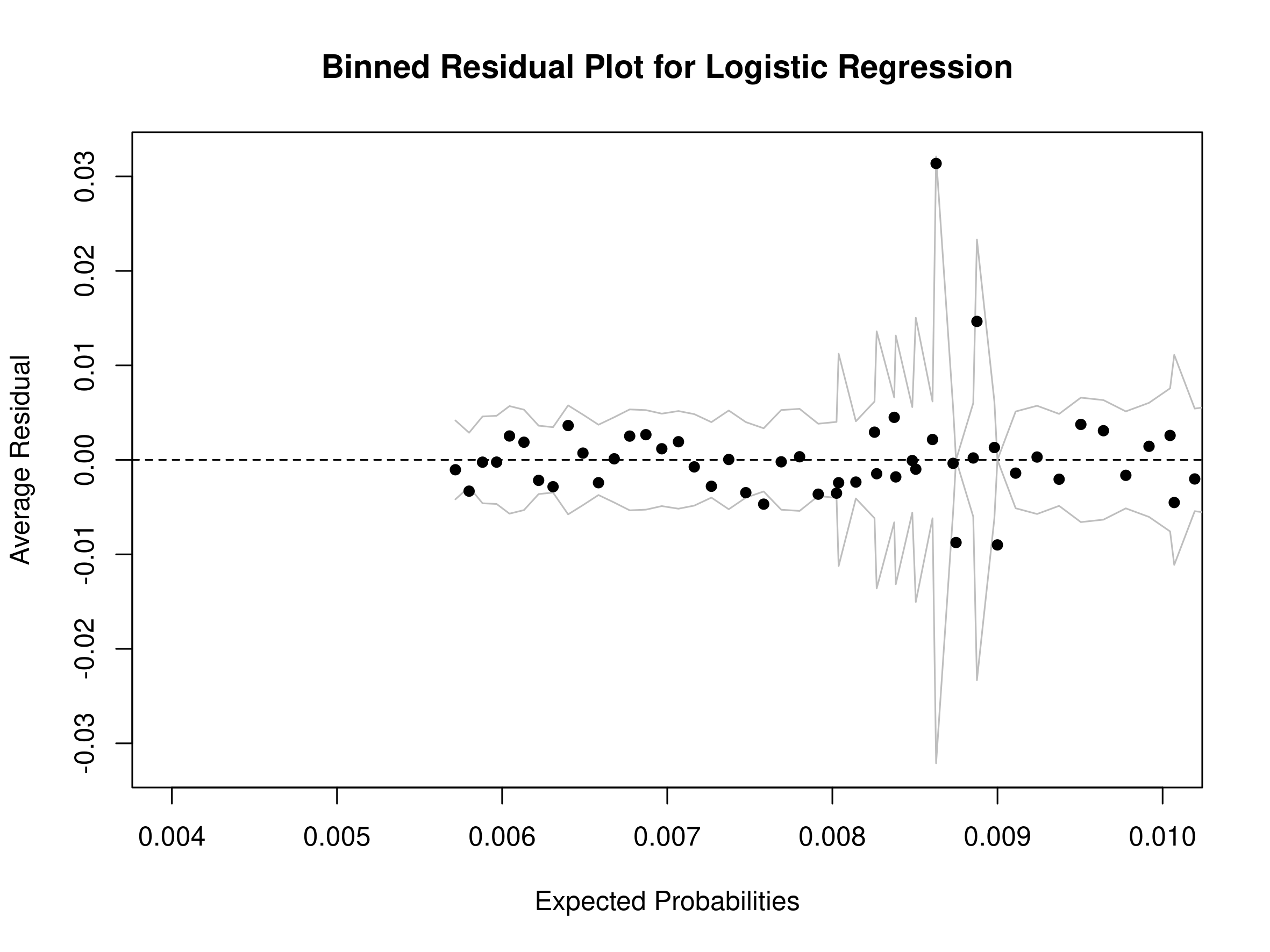


**Note:** this plot corresponds to the model for females and males aged 60-75 in Table 3. The model did not include covariates: *education, smoking, tsi1, tsi2, tsi3, and tsi4.*

**Supplementary Figure 4.6** Binned residuals plot for the model with covariates for the range for the expected probabilities xlim=0.010-0.030

**
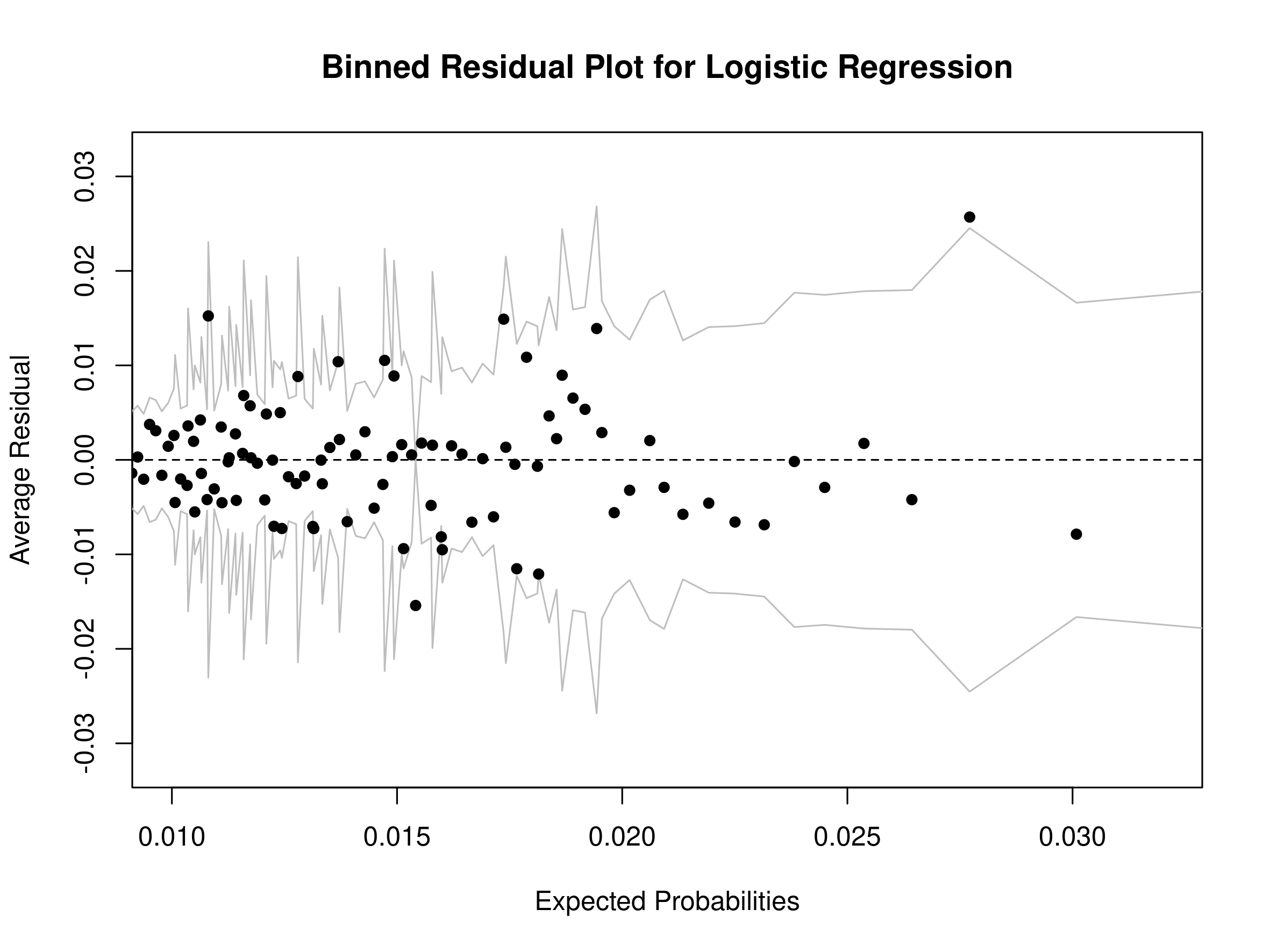
**

**Note:** this plot corresponds to the model for females and males aged 60-75 in Table 3. The model included covariates: *education, smoking, tsi1, tsi2, tsi3, and tsi4.*

The following two figures Supplementary Figure 4.7 and Supplementary Figure 4.8 correspond to the model for females and males aged 60-75 in Table 3. The model included covariates: *education, smoking, tsi1, tsi2, tsi3, and tsi4*

**Supplementary Figure 4.7** Binned residuals plot for the model with covariates for the range for the expected probabilities xlim=0.004-0.010


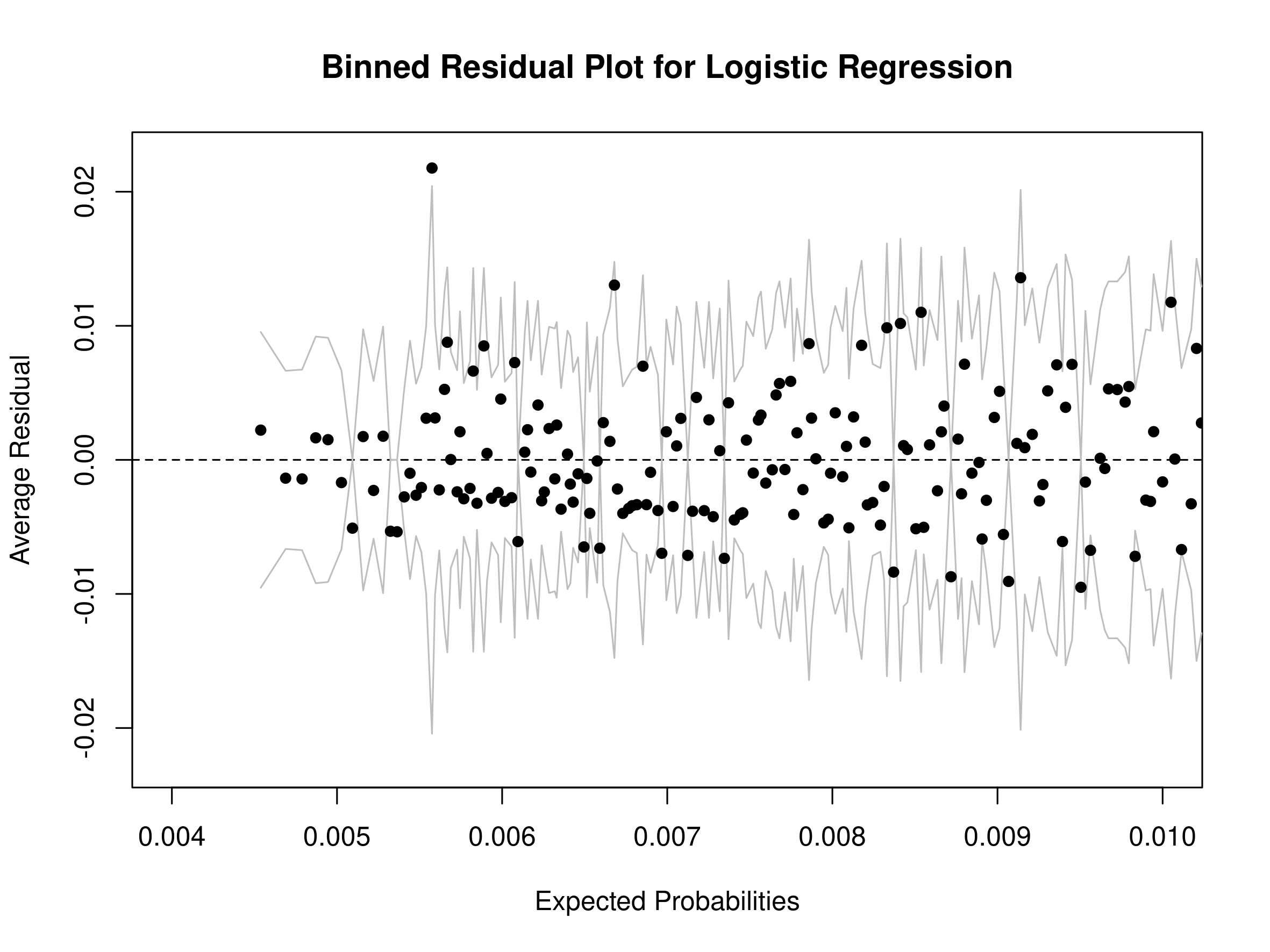


**Note:** this plot corresponds to the model for females and males aged 60-75 in Table 3. The model included covariates: *education, smoking, tsi1, tsi2, tsi3, and tsi4.*

**Supplementary Figure 4.8** Binned residuals plot for the model with covariates for the range for the expected probabilities xlim=0.010-0.032


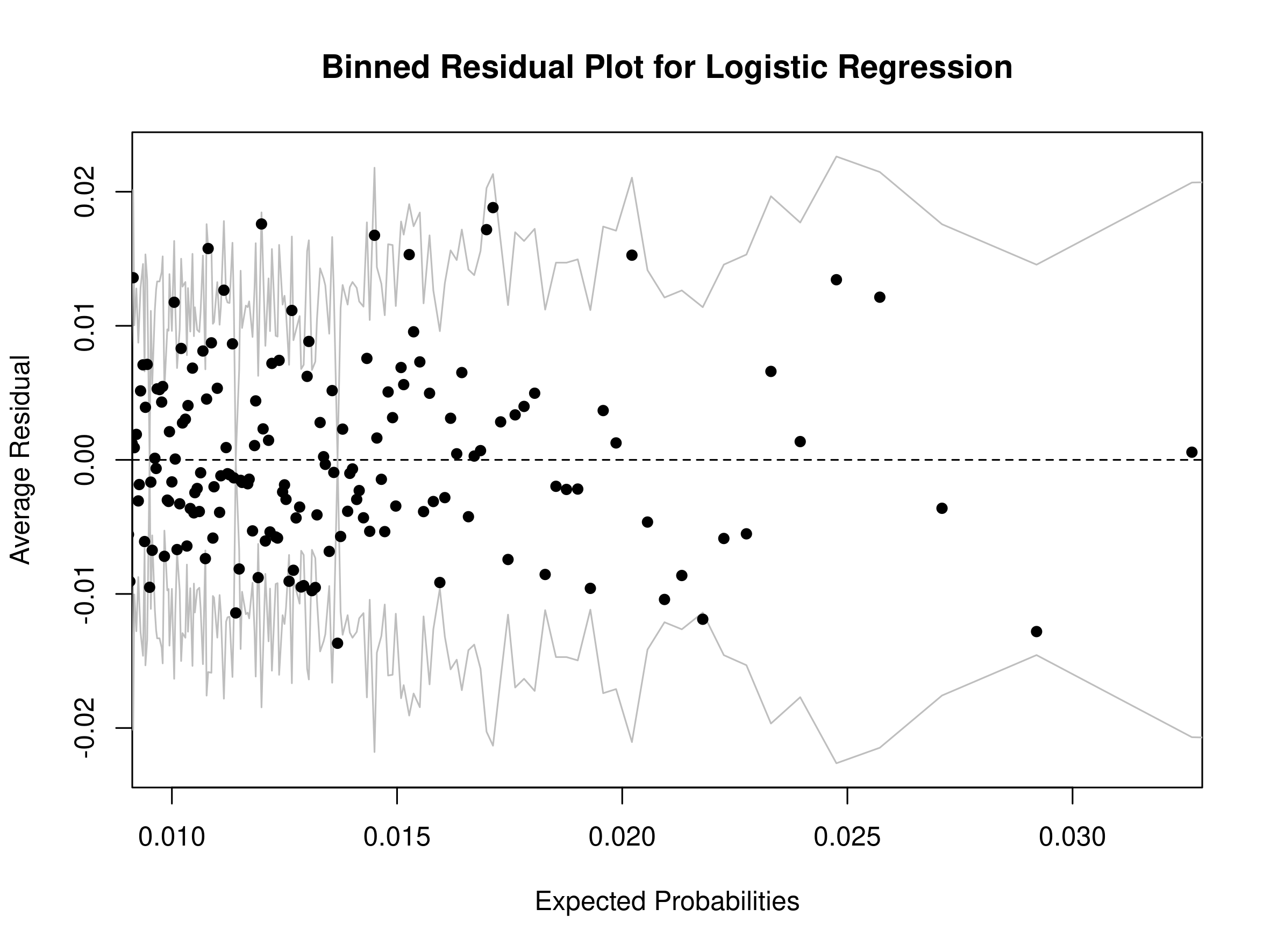


**Note:** this plot corresponds to the model for females and males aged 60-75 in Table 3. The model included covariates: *education, smoking, tsi1, tsi2, tsi3, and tsi4.*

In the binned residual plots presented above, about 95% of the points fall within the 95% confidence intervals and no obvious systematic patterns at the full scale of expected probabilities were observed. In some ranges of the data, the model over- or under-predicts. For example, in Supplementary Figure 4.6 there are points (for the expected probabilities around 0.025) that are located above the horizontal line at zero, which means these prediction might be overestimated.

On the whole, the binned residuals plots supported the linearity assumption in the log-odds and a good level of the models fit.

In the binned residual plots presented above, about 95% of the points fall within the 95% confidence intervals and no obvious systematic patterns at the full scale of expected probabilities were observed. In some ranges of the data, the model over- or under-predicts. For example, in Supplementary Figure 4.8 there are points (for the expected probabilities around 0.025) that are located above the horizontal line at zero, which means these prediction might be overestimated.

On the whole, the binned residuals plots supported the linearity assumption in the log-odds and a good level of the models fit.
